# Global Morbidity and Mortality of Leptospirosis: A Systematic Review

**DOI:** 10.1371/journal.pntd.0003898

**Published:** 2015-09-17

**Authors:** Federico Costa, José E. Hagan, Juan Calcagno, Michael Kane, Paul Torgerson, Martha S. Martinez-Silveira, Claudia Stein, Bernadette Abela-Ridder, Albert I. Ko

**Affiliations:** 1 Oswaldo Cruz Foundation, Brazilian Ministry of Health, Salvador, Bahia, Brazil; 2 Institute of Collective Health, Federal University of Bahia, UFBA, Salvador, Brazil; 3 Department of Epidemiology of Microbial Diseases, Yale School of Public Health, New Haven, Connecticut, United States of America; 4 Center for Analytical Sciences, Yale School of Public Health, New Haven, Connecticut, United States of America; 5 Section of Epidemiology, Vetsuisse Faculty, University of Zürich, Zürich, Switzerland; 6 Division of Information, Evidence, Research and Innovation, World Health Organization, Regional Office for Europe, UN City, Copenhagen Ø, Denmark; 7 Department for the Control of Neglected Tropical Diseases, World Health Organization, Genève, Switzerland; University of Tennessee, UNITED STATES

## Abstract

**Background:**

Leptospirosis, a spirochaetal zoonosis, occurs in diverse epidemiological settings and affects vulnerable populations, such as rural subsistence farmers and urban slum dwellers. Although leptospirosis is a life-threatening disease and recognized as an important cause of pulmonary haemorrhage syndrome, the lack of global estimates for morbidity and mortality has contributed to its neglected disease status.

**Methodology/Principal Findings:**

We conducted a systematic review of published morbidity and mortality studies and databases to extract information on disease incidence and case fatality ratios. Linear regression and Monte Carlo modelling were used to obtain age and gender-adjusted estimates of disease morbidity for countries and Global Burden of Disease (GBD) and WHO regions. We estimated mortality using models that incorporated age and gender-adjusted disease morbidity and case fatality ratios. The review identified 80 studies on disease incidence from 34 countries that met quality criteria. In certain regions, such as Africa, few quality assured studies were identified. The regression model, which incorporated country-specific variables of population structure, life expectancy at birth, distance from the equator, tropical island, and urbanization, accounted for a significant proportion (R^2^ = 0.60) of the variation in observed disease incidence. We estimate that there were annually 1.03 million cases (95% CI 434,000–1,750,000) and 58,900 deaths (95% CI 23,800–95,900) due to leptospirosis worldwide. A large proportion of cases (48%, 95% CI 40–61%) and deaths (42%, 95% CI 34–53%) were estimated to occur in adult males with age of 20–49 years. Highest estimates of disease morbidity and mortality were observed in GBD regions of South and Southeast Asia, Oceania, Caribbean, Andean, Central, and Tropical Latin America, and East Sub-Saharan Africa.

**Conclusions/Significance:**

Leptospirosis is among the leading zoonotic causes of morbidity worldwide and accounts for numbers of deaths, which approach or exceed those for other causes of haemorrhagic fever. Highest morbidity and mortality were estimated to occur in resource-poor countries, which include regions where the burden of leptospirosis has been underappreciated.

## Author Summary

Leptospirosis is a zoonotic bacterial disease that affects vulnerable populations such as rural subsistence farmers and urban slum dwellers. Although leptospirosis causes life-threatening clinical manifestations, such as pulmonary hemorrhage syndrome, and has a worldwide distribution, the key barrier to addressing this neglected disease has been insufficient data on its disease burden. We searched published literature and grey literature studies on leptospirosis and using information collected on disease incidence and case fatality, estimated leptospirosis incidence and mortality at country, regional and global level. Overall leptospirosis was estimated to cause 1.03 million cases and 58,900 deaths each year. These estimates place leptospirosis as a leading zoonotic cause of morbidity and mortality. In addition, morbidity and mortality was greatest in the poorest regions of the world and in areas where surveillance is not routinely performed.

## Introduction

Leptospirosis is a zoonotic bacterial disease that occurs in diverse epidemiological settings but imparts its greatest burden on resource-poor populations [1–6]. The disease has a broad geographical distribution due to the large spectrum of mammalian hosts that harbour and excrete the spirochete agent from their renal tubules [1,3,7]. Leptospirosis affects risk groups that are exposed to animal reservoirs or contaminated environments, such as abattoir and sewage workers, military personnel, and individuals partaking in water sports and recreation [8–12]. However, leptospirosis has a broader health impact as a disease of impoverished subsistence farmers [13–15], cash croppers, and pastoralists [16] from tropical regions.

Furthermore, leptospirosis has emerged as a health threat in new settings due the influence of globalization and climate. Disasters and extreme weather events are now recognized to precipitate epidemics [6]. The emergence of leptospirosis in Thailand [17] and Sri Lanka [18] highlight the potential for the disease to rapidly spread and cause large unexplained nationwide outbreaks. Finally, the expansion of urban slums worldwide has created conditions for rat-borne transmission [19–24]. Urban epidemics are reported in cities throughout the developing world [6,19,25] and will likely intensify as the world’s slum population doubles to two billion by 2030 [26].

The major burden attributed to leptospirosis has been its severe life-threatening manifestations. Leptospirosis has emerged as an important cause of pulmonary haemorrhage syndrome [27–30] and acute kidney injury due to Weil’s disease [31] in many regions where transmission is endemic. Case fatality for pulmonary haemorrhage syndrome and Weil’s disease is more than 10% and 70% respectively [14]. In addition, leptospirosis is increasingly recognized as an important cause of undifferentiated fever [16,32–38]. The majority of leptospirosis patients are not recognized or misdiagnosed as malaria [16], dengue [39–41], and other causes of an acute febrile illness. The lack of an adequate diagnostic test [42,43] has further contributed to under-reporting of cases [44,45], as well as deaths [39]. Underestimation of the morbidity and mortality due to leptospirosis is therefore common [44] and has directly contributed to its neglected disease status.

The lack of reliable estimates of the leptospirosis burden has hampered efforts to formulate the investment case to address key barriers, such as improved diagnostics, and identify effective prevention and control measures. Leptospirosis is amenable to *One Health* approaches to intervention [46], since it is an animal health problem and a cause of economic loss in the same impoverished settings where the human disease burden is high. However, current estimates of cases and deaths rely on national surveillance data compiled from selected countries [47]. Pappas et al performed a review of reports and published literature, which identified regions with high endemicity [7]. Attempts have not been made to systematically estimate the global and regional disease burden, as has been done for other neglected diseases in the Global Burden of Disease (GBD) Study 2010 [48]. The World Health Organization (WHO) convened the Leptospirosis Epidemiology Reference Group (LERG) to guide this task [44]. Herein, we present the findings of a study that aimed to perform a systematic literature review of the data on leptospirosis morbidity and mortality, estimate the annual burden of cases and deaths, and identify GBD and WHO regions with the highest burden to inform local decision making and policy.

## Methods

Methods are presented in detail in the accompanying supplementary document (S1 Protocol). The systematic literature review and quality assurance processes were developed during two consultative meetings of the LERG [49,50]. The findings of the systematic review reported under PRISMA guidelines [51] (S1 Protocol). This independent panel of experts reviewed and provided advice on the methods and interpretation of results for the study.

### Data selection and extraction

**Fig 1:**
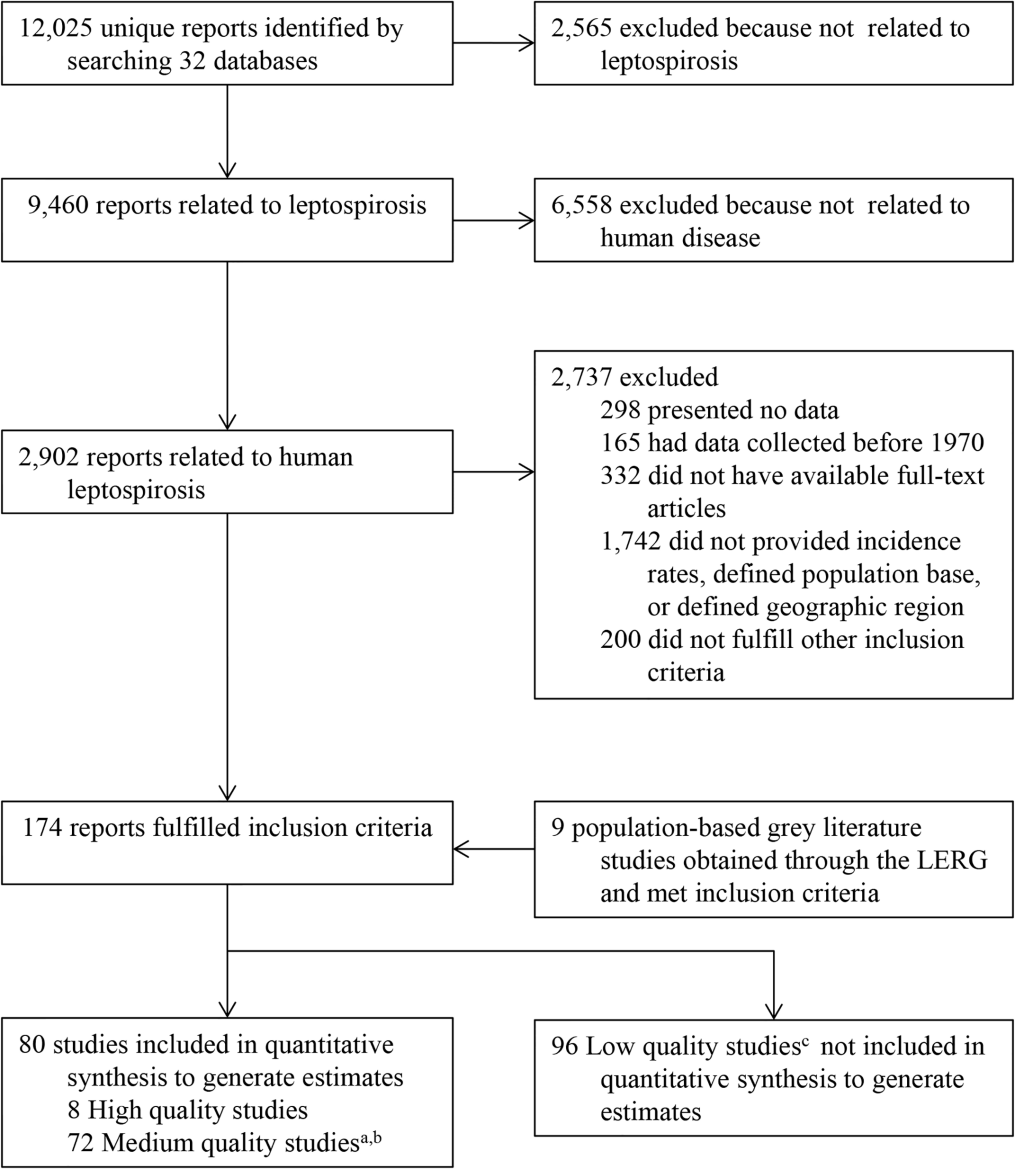
Flow diagram for selection of studies. ^a^65 published and 7 grey literature studies. ^b^Two published reports (S1 Protocol references [39],[56]) were each separated into two studies as they contained separate data from urban and rural populations. ^c^94 published and 2 grey literature studies.

The systematic review covered published reports and grey literature on leptospirosis morbidity and mortality from January 1970 to October 2008. We performed a systematic review of published literature by screening 32 electronic databases (Fig 1), for search terms (S1 Protocol p.2), without language limitations, according to Preferred Reporting Items for Systematic Reviews and Meta-Analyses (PRISMA) guidelines [51]. We defined all variables for which data were extracted (S2 Protocol). In addition, the LERG requested public health officials and researchers to provide supplementary information from published studies as well as grey literature. Studies that fulfilled the selection criteria (S1 Protocol pp. 3–5) were evaluated for methodology and study design and assigned to four quality assurance categories by two independent raters (S1 Protocol p.5, S1 and S2 Tables). For studies that met the study quality criteria (S3 Table), we applied LERG-approved definitions (S1 Protocol p. 5) for confirmed leptospirosis cases and deaths and extracted information on crude disease incidence and case fatality ratio. The systematic review also identified case series of leptospirosis patients among the quality assured incidence studies, and extracted information on age and gender-stratified proportions of cases and deaths (S1 Protocol pp. 4–5; S5 Table). Since standard serologic confirmation of leptospirosis requires evaluation of paired acute and convalescent-phase sera [52], we reviewed laboratory confirmation procedures and extracted data on proportions of suspected cases that had incomplete diagnostic evaluation (S1 Protocol p. 5) and ratios of clinically-suspected to laboratory-confirmed cases and deaths (S1 Protocol p. 5, S7 Table).

### Statistical analysis and reporting

**Fig 2:**
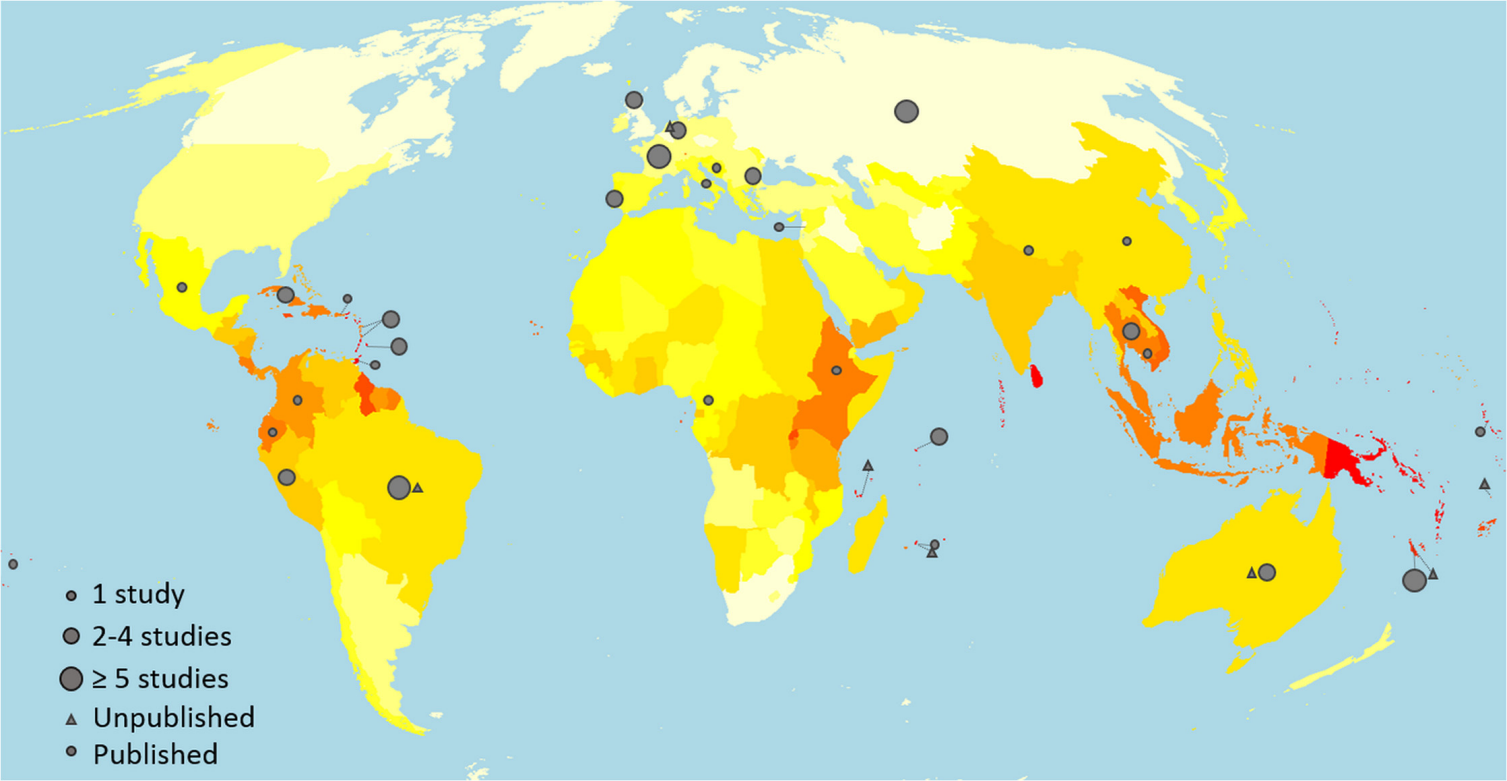
Estimated annual morbidity of leptospirosis by country or territory. Annual disease incidence is represented as an exponential colour gradient from white (0–3), yellow (7–10), orange (20–25) to red (over 100), in cases per 100,000 population. Circles and triangles indicate the countries of origin for published and grey literature quality-assured studies, respectively.

**Fig 3:**
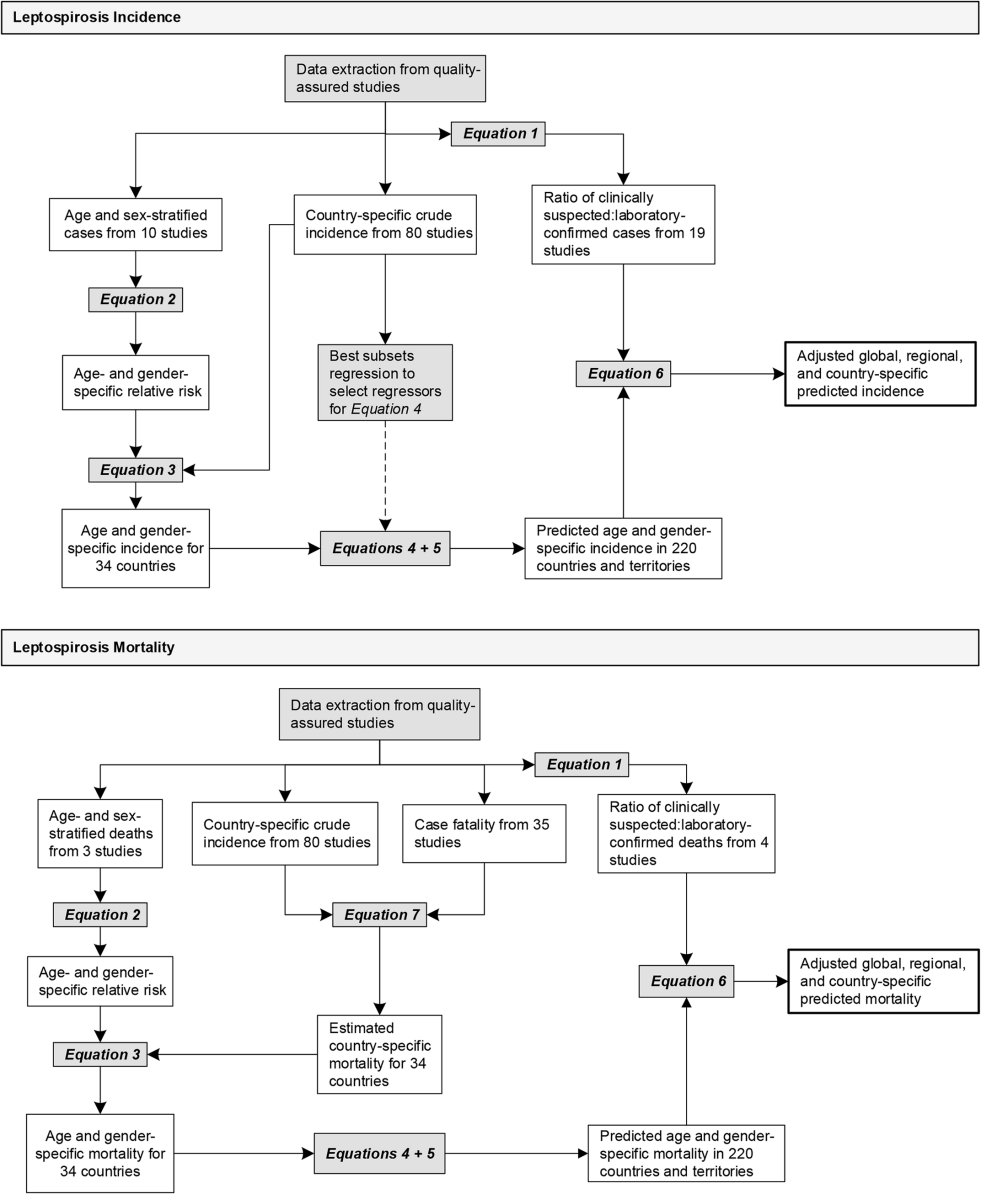
Approach used to estimate global leptospirosis morbidity and mortality. We extracted information on leptospirosis disease incidence, case fatality, age and gender distribution of cases and deaths, and the ratios of clinically suspected to laboratory confirmed cases and deaths from studies that met quality assurance criteria. Data outputs are depicted in open boxes. Processes and analyses are depicted in shaded boxes. Equations are detailed in S10 Table.

We evaluated quality-assured studies for sources of heterogeneity due to study design, epidemiological setting, time, and geographic region. When multiple data sources were available for a country, mean estimates of crude country-specific morbidity were calculated, weighted by the size of the study population. Since information on mortality and case fatality was sparse (S4 Table and Fig 2), we calculated the mean case fatality ratio using all reported data, weighted by study population, and used this estimate together with crude country-specific morbidity to calculate crude country-specific mortality (Fig 3, S10 Table, equation 7 and S1 Protocol p. 6). The majority of studies did not report age and gender-specific incidences. We therefore used the crude country-specific morbidity and mortality estimates, together with data on age and gender-specific risk for disease and death identified from case series reports (S10 Table, equation 2; and S5 and S6 Tables, Fig 3 and S1 Protocol p. 5), to obtain estimates for age and gender-specific morbidity and mortality for countries and territories that had quality-assured data (S10 Table, equation 3, Fig 3 and S1 Protocol pp. 5–6).

Because data were not available for every sub-region, a multivariable regression model was developed to estimate leptospirosis incidence and mortality for each country and territory. We estimated the age and gender-specific morbidity and mortality and their 95% confidence intervals for each of 222 of the world’s countries and territories, (S12 Table) based on a model that was developed with data on age and gender-specific incidences from quality-assured studies (S1 Protocol pp. 6–9). After a range of multivariable regression approaches and candidate variables were evaluated to select a country-level prediction model for age and gender-specific morbidity, we used a linear regression model approach to predict the log-transformation of leptospirosis morbidity based on country-specific indicator variables (S10 Table, equations 4 and 5, S9 Table and Fig 3). This model aimed to derive estimates based on the relationship between the mean reported leptospirosis incidence and country-level characteristics such climate, sociodemographic indicators and health indicators. Variables were screened based on plausibility, availability for all countries and territories, and univariable relationship with leptospirosis incidence. The final variables in the prediction model were selected to produce the highest adjusted R^2^ in order to yield the smallest prediction error: 1) whether the country is a tropical island, 2) percent urbanization of the population, 3) Distance from the equator in degrees latitude, and 4) the mean years of life expectancy at birth. Since crude mortality was calculated directly from disease incidence estimates, we used the same variables to model age and gender-specific mortality at the country level.

We used a Monte Carlo model, which incorporated age and gender-specific incidence estimates and 95% CI for each country and territory as inputs, to obtain country, region, and global estimates and 95% CI of leptospirosis morbidity, mortality, cases and deaths (S10 Table equation 6, Fig 3 and S1 Protocol pp. 7–8). These estimates were used to create probability distributions of age and gender-specific incidence and mortality from which random samples were drawn. The ratios of clinically-suspected to laboratory-confirmed cases and deaths and their 95% CI, obtained from case series reports (Fig 3 and S10 Table equation 1), were used to create normal distributions for the estimated under-reporting ratio for cases and deaths. A random draw from these normal distributions was multiplied by each random sample from estimated incidence and mortality distributions in order to obtain estimates adjusted for incomplete diagnostic testing (S1 Protocol p.7). Population estimates for 2010 were obtained from United Nations Population Division [53]. Morbidity and mortality estimates were calculated for both GBD [48] and WHO [54] geographical regions, described on S1 Protocol p. 2, so that these figures can be compared with information on other neglected diseases. Estimates were rounded to three significant figures, with a precision limit of 100 cases or deaths. Modelling was performed using the R statistical language [55], and Monte Carlo simulation was performed using the Poptools plug-in for Microsoft Excel 2007 [56]. Maps were created to illustrate estimated morbidity using the *rworldmap* package for R [57]. Country-specific estimates of leptospirosis mortality and morbidity were shared with each country in compliance with WHO guidelines.

## Results

The search strategy and quality assessment and data extraction process yielded eight high-quality and 72 medium-quality studies, including seven grey literature studies (S3 Table and Fig 1). Inter-evaluator agreement for the quality assessment was high (Kappa 0.93, 95% CI 0.80–1.00). The majority of studies reported data that were published after 1989 (66%) and obtained from five regions, Western Europe (n = 15; 19%), Caribbean (n = 14; 18%), South-East Asia (n = 10; 13%), Tropical Latin America (n = 10; 13%), and Oceania (n = 8; 10%). Among studies, 96% used hospital-based surveillance to identify leptospirosis cases, while 4% performed case ascertainment in community-based outpatient facilities.

Reported disease incidence ranged from 0.10 to 975.00 annual cases per 100,000 population (S3 and S8 Tables). We did not identify significant temporal trends in morbidity or mortality (considering 10-year periods), but found differences in reported morbidity and mortality based on study design and population (Table 1). Studies that used active surveillance to identify leptospirosis cases reported significantly higher morbidity than passive surveillance studies (12.09 vs. 2.13 per 100 000 population, p<0.01). Morbidity was also significantly higher in studies of rural populations and tropical regions compared to urban settings.

**Table 1:** Reported leptospirosis morbidity, mortality, and case fatality, according to study characteristic.

Characteristic	Morbidity^a^ (N = 80)		Mortality^a^ (N = 35)		Case fatality (N = 35)	
	N (%)	Median (IQR)	N (%)	Median (IQR)	N (%)	Median (IQR)
Decade						
1970–1979	9 (11)	6.25 (1.80–11.75)	4 (11)	1.34 (0.38–2.75)	4 (11)	7.31 (5.62–10.17)
1980–1989	19 (24)	12.41 (0.84–47.69)	8 (23)	1.27 (0.15–2.07)	8 (25)	5.81 (3.68–9.60)
1990–1999	26 (33)	3.11 (1.03–10.18)	13 (37)	0.15 (0.09–0.40)	13 (36)	8.00 (4.80–14.29)
2000–2009	26 (33)	4.95 (0.90–32.56)	10 (29)	0.15 (0–1.07)	10 (28)	0.64 (0.09–5.87)
Surveillance						
Active	28 (35)	12.09^b^ (4.66–57.91)	16 (45)	0.77^c^ (0.31–1.79)	16 (45)	4.99 (1.37–8.01)
Passive	52 (65)	2.13 (0.60–1.71)	19 (55)	0.15 (0.06–0.49)	19 (55)	6.59 (1.96–13.10)
Climate						
Tropical	50 (63)	12.91^b^ (6.26–52.15)	26 (74)	0.66^b^ (0.02–1.71)	26 (74)	4.99 (0.99–8.03)
Temperate	30 (38)	0.65 (0.37–1.88)	9(26)	0.06 (0.23–0.09)	9(26)	10.70 (6.59–11.90)
Setting						
Rural	7 (9)	39.85^d^ (20.27–287.99)	4 (11)	0.18 (0–0.68)	4 (11)	0.24 (0–1.56)
Urban	14 (18)	9.59 (3.00–28.20)	4 (11)	0.66 (0.51–0.73)	4 (11)	3.58 (1.88–5.71)
Mixed	59 (74)	3.02 (0.60–12.91)	27 (78)	0.34 (0.09–1.69)	27 (78)	8.00 (3.26–11.77)

NA, not applicable.

^a^Annual leptospirosis morbidity and mortality rates are shown as cases or deaths, respectively, per 100 000 population, and evaluated based on numbers of determined by reported laboratory-confirmed cases and deaths, respectively.

^b^ p<0.01

^c^ p<0.05

^d^ p<0.05 (comparing incidence from studies of urban or rural populations).

**Fig 4:**
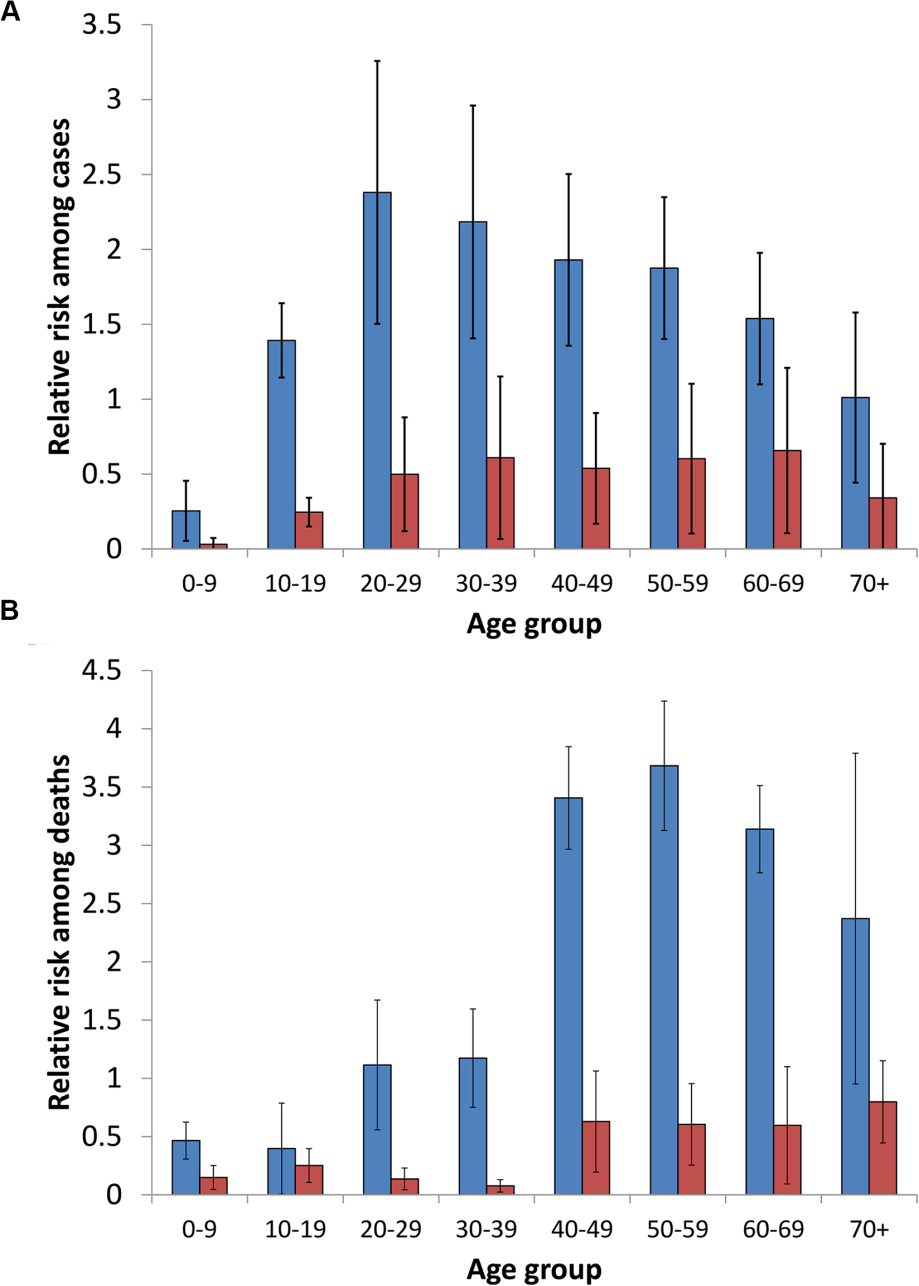
Mean relative risk for membership in age and gender groups among leptospirosis cases (A) and deaths (B). Mean and standard deviation of the relative risks are presented for males (blue bars) and females (red bars).

Among the 35 studies that reported information on case fatality ratios (S3 Table); the mean case fatality ratio was 6.85% (95% CI 5.66–8.03). Ten studies reported age- and gender-stratified data for leptospirosis cases (listed in S5 Table). Adults and males had a greater risk for leptospirosis than children and females (S6 Table and Fig 4A), with highest risk (RR, 2.4, 95% CI 0.7–4.1) occurring among adult males with 20–29 years of age. Among three studies with age- and gender-stratified data for deaths from leptospirosis (S5 Table), the age-specific risk for death was different from that for disease (S6 Table and Fig 4B), and the highest risk for death occurred in an older age group of males with 50–59 years of age (RR, 3.7, 95% CI 2.6–4.8). Among 10 studies that reported information on the completeness of laboratory confirmation procedures, paired samples were obtained from a mean of 53% of cases (range, 20–88%). A total of 19 and four studies reported data on both clinically-suspected and laboratory-confirmed cases and deaths, respectively, due to leptospirosis (S7 Table). Among these studies, the mean ratio of clinically-suspected to laboratory-confirmed cases and deaths was 3.1 (95% CI 1.2–5.1) and 2.2 (95% CI, 0.9–3.3), respectively.

The model selection process screened 147 candidate variables for entry in a multivariable regression model of leptospirosis morbidity (S1 Protocol pp. 6 and 7). Eight variables met statistical, plausibility, and availability screening criteria and were evaluated in multivariable regression models. A linear regression model yielded the best fit multivariable prediction model (Tables 2, S9 and S10, equation 4). This model predicted the natural logarithm of leptospirosis morbidity based on four country-specific variables related to geography and climate (distance from the equator, location on a tropical island), indicators of the population’s overall socioeconomic and health status (life expectancy at birth), and urbanization. The adjusted R^2^ of the prediction model was 0.600. This model was used to estimate age and gender-specific morbidity and mortality for 222 countries.

**Table 2:** Variables incorporated in the final multivariable linear regression prediction model for leptospirosis morbidity in 34 countries.

Variable^a^	R Squared
Distance from the equator	0.105
Percent urbanization of population	0.243
Life expectancy at birth	0.405
Tropical island	0.478
Complete model	0.600^b^

^a^ Definitions and sources of variables are described in S1 Protocol pp. 6–7.

^b^ Adjusted R^2^ is the adjusted proportion of the variance explained by the complete model, with adjustment for the number of variables in the model.

A Monte Carlo model incorporated age and gender-specific incidence and mortality at the country level to obtain country-specific, regional, and global estimates for incidence and mortality that were adjusted for incomplete diagnostic testing (Table 3, S11 and S12 Tables). The annual morbidity and mortality due to leptospirosis worldwide was estimated to be 14.77 cases per 100,000 population (95% CI 4.38–25.03) and 0.84 deaths per 100,000 population (95% CI 0.34–1.37), respectively. Highest disease incidences were estimated in GBD regions of Oceania (150.68 cases per 100,000, 95% CI 40.32–272.29), South-East Asia (55.54, 95% CI 20.32–99.53), Caribbean (50.68, 95% CI 14.93–87.58), and East Sub-Saharan Africa (25.65, 95% CI 9.29–43.31) (Fig 2 and Table 3). Small tropical islands had high estimated incidence of leptospirosis; however, in several cases there was also significant uncertainty associated with those predictions. Morbidity and mortality by WHO sub-region (S11 Table) by country, (S12 Table) and stratified by age and gender (S13 Table) are detailed in the S1 Protocol.

**Table 3:** Estimated annual leptospirosis morbidity and mortality according to GBD region.

GBD region	Morbidity^a^	Cases	Mortality^a^	Deaths
	Estimate (95% confidence interval of the prediction)			
All GBD regions	14.77 (4.38–25.03)	1,030,000 (434,000–1 750,000)	0.84 (0.34–1.37)	58,900 (23,800–95,900)
High Income Asia Pacific	6.95 (2.51–11.87)	14,800 (5,300–25,100)	0.31 (0.13–0.52)	700 (300–1,100)
Central Asia	5.53 (2.07–9.51)	4,400 (1,600–7,300)	0.29 (0.12–0.49)	200 (100–400)
East Asia	10.28 (3.58–18.28)	142,000 (49,400–252,000)	0.50 (0.19–0.88)	6,900 (2,600–12,200)
South Asia	17.97 (6.20–32.25)	289,000 (99,800–519,000)	1.02 (0.41–1.71)	16,500 (6,500–27,600)
South–East Asia	55.54 (20.32–99.53)	266,000 (97,500–477,000)	2.96 (1.65–4.93)	14,200 (5,600–24,000)
Australasia	9.13 (2.79–16.36)	2,400 (700–4,200)	0.40 (0.16–0.69)	100 (0–200)
Caribbean	50.68 (14.93–87.58)	22,300 (6,700–34,700)	2.90 (1.14–4.72)	1,300 (500–1,900)
Central Europe	4.02 (1.21–6.85)	4,800 (1,500–8,200)	0.21 (0.08–0.33)	200 (100–400)
Eastern Europe	1.43 (0.51–2.69)	2,900 (1,100–5,500)	0.09 (0.04–0.16)	200 (100–300)
Western Europe	3.90 (1.45–6.49)	16,300 (6,100–27,100)	0.18 (0.07–0.29)	800 (300–1,200)
Andean Latin America	21.90 (7.92–39.82)	11,700 (4,200–21,200)	0.96 (0.37–1.68)	500 (200–900)
Central Latin America	15.77 (5.83–27.37)	36,000 (12,300–62,400)	0.68 (0.27–1.15)	1,600 (600–2,600)
Southern Latin America	3.87 (1.43–6.74)	2,400 (900–4,100)	0.18 (0.07–0.31)	100 (0–200)
Tropical Latin America	13.53 (4.47–26.56)	27,300 (9,000–53,200)	0.66 (0.23–1.28)	1,300 (500–2,600)
North Africa / Middle East	7.30 (2.58–11.79)	33,300 (11,800–53,800)	0.34 (0.14–0.56)	1,600 (600–2,500)
High Income North America	3.64 (1.02–6.50)	12,800 (3,600–22,900)	0.18 (0.07–0.31)	600 (200–1,100)
Oceania	150.68 (40.32–272.29)	16,700 (4,500–30,200)	9.61 (3.56–17.11)	1,100 (400–1,900)
Central Sub–Saharan Africa	13.49 (4.48–23.56)	13,100 (4,400–22,900)	1.33 (0.52–2.23)	1,300 (500–2,200)
East Sub–Saharan Africa	25.65 (9.29–43.31)	91,100 (33,000–154,000)	1.87 (0.79–3.12)	6,700 (2,800–11,100)
Southern Sub–Saharan Africa	3.44 (1.34–5.78)	2,400 (900–4,100)	0.33 (0.13–0.53)	200 (100–400)
West Sub–Saharan Africa	9.67 (3.62–16.16)	32,000 (12,000–53,500)	0.85 (0.35–1.36)	2,800 (1,200–4,500)

^a^Annual leptospirosis morbidity and mortality rates are shown as cases or deaths, respectively, per 100,000 population.

The model estimated that worldwide there are 1,030,000 cases (95% CI, 434,000–1,750,000) and 58,900 deaths (95% CI, 23,800–95,900) due to leptospirosis annually (Table 3). The majority of leptospirosis cases and deaths occur in tropical regions; 73% of the world’s leptospirosis cases and deaths occur in countries situated between the Tropics of Cancer and Capricorn. Highest morbidity occurred among males with 20–29 years of age (35.27 cases per 100,000, 95% CI 13.79–63.89), while highest estimated mortality occurred in older males with 50–59 years of age (2.89 deaths per 100,000, 95% CI 1.22–4.95). A significant proportion of global burden of cases and deaths due to leptospirosis occurred in the demographic group of males with 20–49 years of age (48% [95% CI 40–61%] and 42% [95% CI 34–53%], respectively).

## Discussion

We estimated that leptospirosis causes 1.03 (95% CI 0.43–1.75) million cases worldwide each year. These estimates place the disease among the leading zoonotic causes of morbidity and mortality. Furthermore, the number of estimated deaths (58,900; 95% CI 23.800–95,900) attributable to leptospirosis approaches or exceeds those for causes of haemorrhagic fever which were investigated in the Global Burden of Disease Study 2010 [48] and other studies [58]. The large majority of the estimated disease burden occurred in tropical regions and the world’s poorest countries. The systematic literature review also found that adult males were the principal risk group for leptospirosis. Based on model predictions, morbidity and mortality was estimated to be high in regions, such as South and Southeast Asia, where leptospirosis is an under-recognized public health problem.

Our approach had to address key challenges in the estimation of leptospirosis burden. First, the available data was sparsely distributed and not representative of all world regions. We therefore developed a model to estimate morbidity and mortality in regions with limited or no information and identified a final model that captured a significant amount of the variability (R^2^, 0.600) in the data from quality-assured studies. Although 95% confidence intervals for estimates were calculated to account for the variability in our assumptions, we may not have accounted for all potential uncertainties. Leptospirosis is an environmentally-transmitted disease [1–3,6]; disease risk may therefore vary significantly within a region, which in turn would contribute to spatial uncertainty. We applied criteria, accepted by an independent panel of experts (LERG), to select studies that employed appropriate methodologies with respect to case definitions, case ascertainment and case confirmation. Yet regional differences in access to health care facilities and laboratory testing, which are not explained by country-specific indicators of health and socioeconomic wealth, may have contributed to unaccounted variation. The true uncertainty may thus be greater than indicated by the confidence intervals for our estimates. Lastly, because specific countries had atypical characteristics, their model-predicted morbidity and mortality had high uncertainty which resulted in inflated estimates due to exponentiation from the log scale, which incorporates the standard error into the estimate. Estimates are therefore most reliable at the regional and global level, and caution should be taken when interpreting individual country estimates.

The second challenge related to incomplete laboratory testing of suspected cases. This is a widespread problem for leptospirosis since case confirmation relies primarily on identifying seroconversion of agglutinating antibodies between acute and convalescent-phase samples [52]. Among studies with information on laboratory confirmation procedures, complete laboratory testing for leptospirosis was not performed in almost 50% of the suspected cases. In order to address this source of under-reporting, we adjusted estimates of morbidity and mortality for the effect of incomplete diagnostic testing. Similar barriers with respect to sparse data and reliance on antiquated serologic tests are shared among many of the neglected diseases [59], and as with leptospirosis, have directly contributed to their neglected disease status. Although our modelling approach has limitations, it may have a more generalizable application in estimating the disease burden for neglected diseases.

Our estimates likely underestimate the morbidity of leptospirosis, since disease incidence data was obtained from hospital-based surveillance studies (S3 Table), the majority (65%) of which used passive case ascertainment. Similarly our estimates of mortality represent an underestimation since these were highly sensitive to estimates of morbidity. We obtained information on case fatality ratios from 35 studies, which included 20 (57%) conducted in World Bank upper income countries. The mean case fatality ratio (6.85%) that we used in modelling mortality is likely a significant underestimation of the ratios that occur in resource-poor regions. Worldwide case fatality ratio, based on estimated cases and deaths, was even lower (5.72%) due to the influence of the worldwide age and sex population structure. We opted to use a conservative assumption when faced with uncertainty, rather than attempt to model regional differences in case fatality ratio, or use ad-hoc adjustments. Our estimates of annual leptospirosis cases are higher than the approximately 500,000 cases estimated based on a survey of national surveillance data [47]. The higher estimates obtained from our study are plausible since this survey was conducted among a convenience sample of Ministries of Health.

The study’s morbidity estimates reflect the incidence of severe leptospirosis, rather than rates for clinical or symptomatic illness, since selected studies used case definitions that relied on detection of severe manifestations [60]. Severe leptospirosis is generally believed to account for a small fraction (5–15%) of all clinical infections [1,14,61]. There is a growing recognition that leptospirosis is an important cause of an acute febrile illness: leptospirosis has been shown to be the cause of 5–69% of acute undifferentiated or non-malarial fever cases in different parts of the world [16,18,32–38,62]. Leptospirosis, as in the case of dengue [58], may therefore account for a much greater burden than indicated by morbidity estimates of severe disease.

The study’s findings highlight the contribution of geography, climate, and poverty in the worldwide distribution of leptospirosis. Countries situated in the tropics had the highest estimated disease incidence and accounted for 73% of the world’s estimated cases. This pattern is attributable to environmental and social conditions which promote the abundance of reservoir animals, survival of the bacterium in soil and surface water, and risk of human exposures with these sources of infection [3,22,23]. Tropical climate also favours transmission of leptospirosis, which is often seasonal and increases during periods of heavy rainfall [6,19]. The disease is well-recognized as a health problem of impoverished rural-subsistence farmers [13,15], pastoralists [16,45,63], and urban slum dwellers [19–22]. We found that life expectancy, which serves in part as a proxy for poverty, was an independent predictor of country-specific disease incidence (Table 2). Finally, although urban slum environments are an emerging and increasingly important setting for leptospirosis transmission [19–22], in our model, country percent urbanization was inversely associated with leptospirosis incidence, reflecting in part the high burden of leptospirosis in rural settings, but also the well-recognized association between lower aggregated country-level percent urbanization and poverty.

The study identified regions within the developing world where the burden of leptospirosis may be significantly under-recognized. The annual morbidity of leptospirosis was estimated to be high in countries of South and Southeast Asia with large populations, such as India (19.7 cases [95% CI 6.8–36.8] per 100,000 population, S12 Table) and Indonesia (39.2 [12.8–78.0] per 100,000 population, S12 Table). Although transmission is endemic and large outbreaks have been reported in these countries [15,62,64], surveillance for leptospirosis has not been routinely performed.

An important limitation of the study was the scarce data on disease burden in specific geographical regions. This was particularly evident for regions within Africa, where information on morbidity and mortality rates was available from two studies. Although the burden estimates may not be reliable for this region, there is increasing evidence suggesting their plausibility. A large spectrum of sylvatic and domestic animals are reservoirs for *Leptospira* in Africa [65,66]; leptospirosis is a recognized animal health problem in the region [4]. A recent systematic review found high seroprevalence among human populations in different settings across the continent [4]. Furthermore, surveys of patient populations have found leptospirosis to be a prevalent cause of acute febrile illness [32,63]. A recent population-based study reported an annual morbidity for leptospirosis of 75–102 cases per 100,000 population in northern Tanzania [45]. Additional locally representative data will be key to validate our estimates for the African continent and other regions with sparse data, though these efforts will require resources and time and may delay decision making with respect to strengthening surveillance and implementing control measures.

Our study was also limited by the lack of studies that reported age and gender-specific incidence for morbidity and mortality. We extracted data from case series of representative patient populations in order to estimate the age and gender-specific risk for leptospirosis, which in turn was incorporated as an input in our models (S1 Protocol pp. 5–8 and Fig 3). We found that the risk of acquiring leptospirosis was higher in adults than children and higher in males than females, and highest among adult males with 20 to 29 years of age (S6 Table and Fig 4A). Male gender preference is a well-recognized phenomenon in leptospirosis and due to the gender-specific occupational and peridomicilary risk activities [22–24,67]. The age and gender-specific risk for death had a different pattern: the risk for death increases with increasing age (S6 Table and Fig 4B), a finding which has been observed in a range of epidemiological settings [14]. Since these estimates were based on small number of case series, additional studies of well-characterized patients from representative sites would therefore improve these estimates. Our systematic review did not include more recent literature from 2008; however there have been few reports on population-based mortality and morbidity rates during this interval. Leptospirosis is caused by a large number of serovar and serogroup agents which vary across regions. We could not evaluate the contribution of such agents on mortality and morbidity estimates, since less than 20% of the studies reported serologic or culture identification of these agents.

Our study provides a baseline estimate to evaluate trends, as processes of climate and land use evolve in the future [6]. By 2037, the majority of the world’s population will be inhabitants of urban centres in developing countries. A large proportion of this population will reside in slum settlements, where poor sanitation has created the conditions for annual rainfall-associated epidemics [22,68]. Extreme weather events and flood-related disasters [6] are predicted to escalate with global climate change [69,70]. As deforestation and agricultural expansion intensify in tropical regions [71], rural-based farming populations may be increasingly exposed to leptospirosis. A formal burden of disease calculation will need to be performed to provide estimates based disability-adjusted life years (DALYs). As a caveat, the health outcomes of leptospirosis have been traditionally associated with its acute disease. The disease causes sub-acute and chronic complications, such uveitis [72], and persistent complaints [73]. However, the frequency and magnitude of long-term sequelae have not been rigorously quantified. Although the disease is life-threatening, the overall DALYs attributable to leptospirosis may be relatively low. Considering the annual number of deaths worldwide, the impact of leptospirosis equals that of canine rabies (59,000 annual deaths) [74]. The burden of leptospirosis, with respect to morbidity, is higher than some other important neglected tropical diseases, including visceral leishmaniasis and severe dengue, and is similar to others, including echinococcosis and cysticercosis [75].

The study provides decision makers with an evidence base to implement effective policy and responses to leptospirosis. As identified in this study and cited in previous reviews [42,43] the lack of an adequate diagnostic test remains a foremost barrier. The demand for improved diagnostics will be greater than indicated by cases estimated in this study, since these estimates reflect the burden of severe leptospirosis and represent a lower boundary for the actual number. The distribution of the leptospirosis burden (Fig 2) overlaps significantly with that for malaria [48], dengue [48,58], and enteric fever [48]. Misdiagnosis between these diseases is common [16,39–41] and in the case of leptospirosis, leads to delayed treatment of severe complications and poor outcomes [40]. Development and roll-out of diagnostic protocols could be leveraged and implemented synergistically that aim to address the multiple causes of acute fever in resource-poor, high-burden regions.

Finally, the estimation of global burden of leptospirosis now provides the opportunity to evaluate *One Health* strategies for prevention and control. The lack of recognition of leptospirosis as an important zoonotic disease had previously hampered consideration of such approaches. Our estimates support the assertion that leptospirosis is a leading zoonotic cause of morbidity and mortality in humans. The majority of the estimated morbidity and mortality occurs in regions which have large subsistence farming and pastoral populations and where the disease is a veterinary health problem and cause of lost productivity. Additional work is needed to quantify the economic burden of leptospirosis, which incorporates an assessment of its impact on animal health. Vaccines for leptospirosis are routinely used in livestock and domestic animals, although they do not appear to be transmission-blocking [5]. Investment towards identifying interventions, such as vaccines, may therefore yield synergistic health and societal benefits for poor populations in developing countries. Moreover, more sustainable practices considering ecosystems [76,77] are needed for disease prevention. Finally, leptospirosis is a social-ecological problem, which often occurs in the context of social inequity. Therefore there is a critical need to evaluate and address the investment case for interventions that target the underlying environmental conditions and infrastructure deficiencies, such as open sewers in urban slum communities [22–24], in order to make sustainable progress against this neglected disease.

## Supporting Information

S1 Checklist PRISMA checklist. (DOC)

S1 Protocol Systematic review protocol. (DOCX)

S2 Protocol Manual of definitions. (DOCX)

S1 Table Quality assessment criteria. (DOCX)

S2 Table Quality assessment checklist. (DOCX)

S3 Table Characteristics and findings of high and medium quality leptospirosis morbidity (N = 80) and mortality (N = 35) studies, according to GBD region. (DOCX)

S4 Table Geographic distribution of high and medium quality studies, according to GBD region (A) and WHO sub-region (B). (DOCX)

S5 Table Studies that reported information on age and gender proportions of cases and deaths from leptospirosis. (DOCX)

S6 Table Relative risk of leptospirosis cases (N = 10 studies) and deaths (N = 3 studies) according to age and gender group. (DOCX)

S7 Table Studies which reported information on both clinically-suspected and laboratory-confirmed leptospirosis cases and deaths. (DOCX)

S8 Table Reported disease morbidity, mortality, and case fatality, according to GBD region (A) and WHO sub-region (B). (DOCX)

S9 Table Model parameters for leptospirosis morbidity and mortality estimation. (DOCX)

S10 Table Equations. (DOCX)

S11 Table Estimated annual leptospirosis morbidity and mortality by WHO sub-region. (DOCX)

S12 Table Estimated leptospirosis morbidity and mortality by country, grouped according to WHO sub-region. (DOCX)

S13 Table Estimated age group and gender-specific leptospirosis morbidity and mortality, according to WHO sub-region. (DOCX)
