# Supplementary material for: Global Morbidity and Mortality of Leptospirosis: A Systematic Review
Source: PLoS Negl Trop Dis. 2015 Sep 17;9(9):e0003898. doi: 10.1371/journal.pntd.0003898 (PMC4574773; doi:10.1371/journal.pntd.0003898)
Supplement: S1 Protocol — (DOCX) [file pntd.0003898.s002.docx]

**S1 Protocol**

**Abstract**

Leptospirosis is an important cause of illness in many regions of the world that affects marginalized populations such as urban slum residents and subsistence farmers. Official surveillance efforts are complicated by technically challenging laboratory diagnosis and diverse and frequently nonspecific manifestations. A systematic estimate of the burden of this disease is needed to enable rational and effective targeting of surveillance and control activities. The Leptospirosis Epidemiology Reference Group (LERG) of the World Health Organization (WHO) undertook an effort to provide a systematic and comprehensive assessment of the available literature on leptospirosis incidence and mortality, and to generate a credible estimate of the number of annual cases and deaths due to this illness. This process identified over 12 000 unique published reports and grey literature studies. Of these, 80 studies from 34 countries met inclusion and quality control criteria, and were used in a mathematical model to predict incidence and mortality in each of 222 countries and territories, summarized by Global Burden of Disease (GBD) region and WHO mortality-stratified sub-region. This appendix presents supplemental data and discuss in detail the methods used to estimate the global morbidity and mortality of leptospirosis.

**Definition of geographical sub-regions**

Countries were grouped according to 21 Global Burden of Disease regions using definitions used in the Global Burden of Disease 2005 study [[1](#_ENREF_1)] and 14 WHO sub-regions with mortality strata (from A, very low child mortality and low adult mortality, to E, high child mortality and very high adult mortality)[[2](#_ENREF_2)]. These groupings are defined based on similarities in epidemiological conditions and all-cause mortality, and allow extrapolation of disease burden information to similar countries.

**Search strategy and selection criteria for published reports and grey literature data from LERG members**

The review covered published and grey literature reports dated from January 1970 to October 2008. Articles for the systematic review were identified by searching for the terms “*Leptospira*” OR “Leptospirosis” OR "Weil disease" OR "Weil's disease" NOT “Leptospirillum” in each of the electronic database sources. Searches were customized for each electronic database according to the database’s individual subject headings and searching structure. In addition, translated words for “leptospirosis” (i.e., *leptospirose* in Portuguese) were included in searches of the regional databases. The search term used was found appropriate for the identification of reports related with the subject of interest and was used in 32 electronic databases. We considered no language restrictions for the systematic review search strategy, we included in the systematic review process and analyses reports written in English, French, Italian, Portuguese, Spanish, Danish, German, Greek, Hungarian, Dutch, Norwegian, Russian, and Slovenian. Reviewers were able to read English, French, Italian, Portuguese, and Spanish. Reports in Danish, German, Greek, Hungarian, Dutch, Norwegian, Russian, and Slovenian were identified and translated by WHO into English and then evaluated by the reviewers.

A questionnaire was sent to LERG members requesting any additional information on leptospirosis incidence from grey literature population-based studies. This questionnaire requested details on study design and characteristics that could be used for quality evaluation to determine if the data met inclusion criteria for analysis together with published data.

**Electronic databases**

The following list includes 32 electronic databases that were assessed for the systematic review:

| Database | Site |
| --- | --- |
| Global databases |  |
| Medline (MED) | http://www.ncbi.nlm.nih.gov/pubmed/ |
| Popline (POP) | http://db.jhuccp.org/ics-wpd/popweb/ |
| CAB (CAB) | [http://www.promedmail.org](http://www.promedmail.org/) |
| Biological Abstracts (BA) | <http://www.periodicos.capes.gov.br/portugues/index.jsp> |
| CINAHL (CIN) | http://www.cinahl.com/ |
| EMBASE (EMB) | Provided by WHO |
| PAIS International (PAIS) | http://www.csa.com/factsheets/pais-set-c.php |
| ProMed (PRO) | [http://www.promedmail.org](http://www.promedmail.org/) |
| ISI Web of KNOWLEDGE (ISI) | http://isiwebofknowledge.com/ |
| Cochrane (CCT) | http://www.cochrane.org/ |
| WHOLIST (WHOL) | <http://bases.bireme.br/cgi-bin/wxislind.exe/iah/online/?IsisScript=iah/iah.xis&base=WHOLIS&lang=p> |
| BIONLINE | [http://www.bioline.org.br](http://www.bioline.org.br/) |
| Regional WHO databases |  |
| African Index Medicus (AIM) | http://indexmedicus.afro.who.int/cgi-bin/wxis.exe/iah/ |
| Index Medicus for the Eastern Mediterranean Region (IMEMR) | http://www.who.int/bookorders/anglais/detart1.jsp?sesslan=1&codlan=1&codcol=46&codcch=15 |
| Western Pacific Region Index Medicus (WPRIM) | http://wprim.wpro.who.int/SearchBasic.php |
| Afro Library (AFRO) | http://afrolib.afro.who.int/ |
| Other regional databases |  |
| Latin American and Caribbean Health Science (LIL) | <http://bases.bireme.br/cgi-bin/wxislind.exe/iah/cys/?IsisScript=iah/iah.xis&base=LILACS&lang=p> |
| BIREME – Virtual health Library – (BIR) | [http://www.bireme.br](http://www.bireme.br/) |
| KoreaMed (KOR) | http://www.koreamed.org/SearchBasic.php |
| Indian Medlars Center – IndMed – (IMED) | http://indmed.nic.in/ |
| Metasearch from HELLIS Network Libraries (HEL) | <http://www.hellis.org/modules.php?op=modload&name=metasearch&file=hellismeta> |
| Health Research and Development Information Network (HRD) | http://www.herdin.ph/old/ |
| AMICUS Canadian union catalogue (AMI) | http://amicus.nlc-bnc.ca/aaweb/amilogine.htm |
| Institute of Tropical Medicine (ITM) in Antwerp Belguim | http://lib.itg.be:8000/webspirs/start.ws |
| Japan Science and Technology Information Aggregator, Electronic (JAP) | http://www.jstage.jst.go.jp/browse/ |
| African Journals on Line (AJOL) | http://www.inasp.info/index.html |
| l'Ecole Nationale de la Santé Publique (BDSP) | http://www.bdsp.tm.fr/ |
| Turkish Medline (TUR) | http://www.turkishmedline.com/ |
| La bibliothèque de Santé Tropicale (BST) | <http://www.santetropicale.com/resume/catalogue.asp> |
| Cuiden (CUI) | http://www.index-f.com/ |
| Databases that could not be used |  |
| SocioFile | http://www.nisc.com/factsheets/soci.asp |
| Econlit | http://www.econlit.org/ |
| BIOSIST | http://www.biosis.org/ |
| African Health Line | [http://www.nisc.com](http://www.nisc.com/) |
| IMSEAR | <http://library.searo.who.int/modules.php?op=modload&name=websis&file=imsear> |

**Database management**

EndNote® bibliographic software was used to store the citations identified in the search, keep track of them and detect duplicates. Electronic search results were downloaded directly into EndNote®. Reports retrieved from other sources (e.g. hand searching, personal contact) were entered in EndNote® manually after the electronic searches. Duplicates were identified, and each study was assigned a unique identification number for the review. All citations identified by the electronic search strategies were initially evaluated according to the screening form on the basis of the titles and abstracts (when available). Irrelevant records were identified and excluded. (A list of the irrelevant records is available upon request.) The full text of the remaining reports was obtained.

**Identifying duplicated references**

Reports were considered duplicated when they had the same information in the author, year of publication, name of the peer review, volume issue and page number fields. To identify duplicated reports Medline was used as reference database, and other databases were added one by one and duplicates identified at each point of the process. The order in which the databases were joined to Medline was: EMBASE, CAB Abstracts, Biological Abstracts, ISI Web of Knowledge, Latin American Caribbean Health S. I., Bireme, CINAHL, L'Ecole nationale de la santé publique, IndMed-Indian Medlars Centre PAIS International., Health Research and Development I.N., Institute of Tropical Medicine (ITM), Japan Science and Technology, I. A., Bioline, Western Pacific Region Index Medicus, WHOLIST, Popline, Cochrane, HELLIS Network Libraries, KoreaMed, Amicus Canadian union catalogue, IND MED East Med. Region, Turkish Medline, Cuiden and African Health Line. For the Pro-Med database, when two mails reported information on the same event, the mail with less information was considered duplicated. Duplicate references were recorded and separated in an Excel database. A list of the duplicate records is available upon request. Each unique study was assigned an identification number for the review.

**Screening reports on human leptospirosis**

We screened all unique reports, irrespective of language, which fulfilled the search criteria for leptospirosis among reports that were contained in designated scientific literature databases and that were published from January 1, 1970 to October 31, 2008. The screening process was duplicated by two independent reviewers, and all discrepancies were resolved by consensus. All unique references were evaluated and entered into a database. Data identified by this means, as well as grey literature reports provided by LERG members that met criteria for human leptospirosis were further classified as containing information related to disease incidence.

**Selection of leptospirosis incidence studies**

Reports were selected for quality assurance evaluation if they met all of the following inclusion criteria:

1. Belongs to one of the following categories of disease incidence studies (see definitions below):

- Nationally representative incidence study
- Community-based incidence study
- Prospective cohort study
- National, regional, or community-based surveillance study

1. Has a defined start and end date for the study described.
2. Describes information relating to endemic transmission of leptospirosis, including endemic patterns with seasonal variation, but not including outbreaks.
3. Describes the population base for which the study was performed OR the geographical region in which the study was performed and for which an estimate of the inhabitant population can likely be obtained (i.e. national census).
4. Describes information on cases and disease rates which occur after January 1, 1970. If reports include information for periods before and after this date, these reports were only included if disaggregated rates for the period after January 1, 1970 were reported or can be calculated.
5. Full text of the report (or a translated version, if applicable) has been obtained by the systematic review team.

**Definitions for disease incidence studies**

The following definitions were used to define categories of disease incidence studies:

1. Nationally representative incidence study: A study that systematically detects cases of leptospirosis that occur in a defined population or set of populations, designed to obtain nationally representative estimates of disease incidence.

- Designed to identify all cases of leptospirosis that occur at the community level during the study period.
- Uses active case detection protocols such as community-based case finding or outpatient surveillance.
- Has a defined population base as determined by investigator-initiated surveys or census.

1. Community-based incidence study: A study that systematically detects cases of leptospirosis that occur in a defined population, designed to obtain estimates of disease incidence that are representative of a specified community or set of communities.

- Designed to capture all cases of leptospirosis that occur at the community level during the study period.
- Uses active case detection protocols such as community-based case finding or outpatient surveillance.
- Has a defined population base as determined by investigator-initiated surveys or census.

1. Longitudinal cohort study: A defined population for which subjects are individually recruited and followed over time with the aim of identifying leptospirosis cases and estimating disease incidence for the cohort population.

- May include cohorts of high-risk populations (i.e., rural subsistence farmers, urban slum dwellers, occupational risk groups).
- Evaluates disease incidence in enrolled cohort subjects who are representative of the population to be studied.

1. National, regional, or community-based surveillance study: Studies that incorporate a continuous and systematic process for identifying cases of leptospirosis on the national level or for a defined regional entity or community.

- Relies on passive reporting of cases that is initiated by health care providers and/or laboratory staff, OR active case detection at health care facilities or laboratories.
- For the purpose of this systematic review on disease burden, active population-based studies were defined as surveillance studies if case finding protocols were performed exclusively at hospital health care facilities or laboratories. Studies that perform active community-based surveillance at outpatient health care facilities were defined as national or community-based disease incidence studies if they fulfil the criteria for these studies (categories 1 and 2).
- Has a defined population base as determined by investigator-initiated surveys or census or studies a defined geographical region for which census information was likely to be obtained.

**Selection of case series for data on age- and gender-susceptibility to leptospirosis**

Studies that met inclusion criteria were evaluated to determine whether they contained sufficient information to use in calculating susceptibility to leptospirosis by age and gender. Studies were used in evaluating the risk for leptospirosis cases and deaths met the following criteria:

1. Described case series of consecutive laboratory-confirmed cases or deaths due to leptospirosis.
   Included information about both gender and age with enough detail to distinguish the following 8 age groups: 0-9, 10-19, 20-29, 30-39, 40-49, 50-59, 60-69, 70+.
2. Described the age and gender for at least 50 laboratory-confirmed cases or at least 10 laboratory-confirmed deaths, for analysis of age and gender-specific susceptibility to leptospirosis and death from leptospirosis, respectively

**Leptospirosis case definitions**

Based on LERG recommendations, the following definitions were used for diagnostic criteria:

Confirmed case of leptospirosis: Clinical signs and symptoms consistent with leptospirosis and any one of the following:

1. Four-fold increase in microagglutination (MAT) titre in acute and convalescent serum samples
2. An MAT titre ≥ 1:400 in single or paired serum samples
3. Isolation of *Leptospira* spp from normally sterile site
4. Detection of *Leptospira* spp in clinical samples by histological/ histochemical/ immunostaining techniques
5. *Leptospira* DNA detected by PCR.

Probable case of leptospirosis: Clinical signs and symptoms consistent with leptospirosis and any one of the following:

1. Presence of IgM antibodies by ELISA or dipstick
2. Presence of IgM/IgA antibodies in the immunofluorescence assay
3. An MAT titre ≥1:100 in single acute-phase serum sample

Clinically-suspected but unconfirmed leptospirosis: Clinical signs and symptoms consistent with leptospirosis that do not meet criteria for laboratory-confirmed or probable case of leptospirosis.

**Quality assessment of disease incidence reports**

Reports were evaluated for methodology and study design in four categories: study population, methods for measuring incidence, sources of bias, and analytic methods used to determine rates. Checklists were prepared to evaluate and score the quality of evidence for disease incidence studies (S1 and S2 Tables). Using these checklists, reports were assigned a category of high, medium, or low quality.

The quality assessment steps were duplicated by two independent reviewers (F.C. and J.C.) for 20% of the reports that met inclusion criteria. A Kappa statistic was calculated for the quality assessment step to evaluate inter-reviewer reliability, and discrepancies in the data were resolved during review of the reports between the reviewers and principal investigator (Kappa 0·93, 95% CI, 0·80 – 1.00).

**Data extraction**

Studies that met criteria for high and medium quality (S3 and S4 Tables) were selected for data extraction and analysis. Data collected included descriptive variables relevant to the inclusion criteria and quality assessment steps, country and local region (if applicable) of the study, and outcome measures related to incidence, mortality and case fatality. We separately extracted information about morbidity, mortality, and case fatality of laboratory-confirmed and unconfirmed cases of leptospirosis according to the definitions listed above. Some reports that met quality assessment criteria for inclusion contained data from multiple study populations. Where possible, we separated studies from a single report if data were presented for epidemiologically distinct settings, such as urban and rural regions. We calculated a mean disease incidence and case fatality ratio using weights according to the study population, if a study presented data sampled over different time periods in the same geographical location. Similarly, we calculated a mean disease incidence and case fatality if a report presented data from different, but related geographical regions (e.g. Kauai and Hawaii islands).

When incidence studies contained case series describing incidence and mortality with detailed information on age and gender of cases and deaths, this information was also extracted. When reports included information about laboratory-confirmed and laboratory-unconfirmed cases from the same time period, we calculated the ratio of confirmed to unconfirmed cases and deaths. When reports included the necessary detail on completeness of diagnostic evaluation, we also extracted this information and calculated the percent of cases that had paired samples available for serological testing.

**Descriptive evaluation of available disease incidence data**

We evaluated the available reports for sources of heterogeneity due to study design, epidemiological setting, time, and geographic region. Median incidence and mortality, and mean case fatality were compared across subgroups based on study design or population characteristics. We used the Wilcoxon Rank Sum test or the Kruskal-Wallis test as appropriate to evaluate for significant differences in disease incidence, mortality, and case fatality based on study characteristic.

**Calculation of estimated incidence and mortality in each of 222 countries and worldwide**

Step 1: Calculate age- and gender-specific estimates for incidence and mortality in countries with available data.

We generated estimates for incidence and mortality that were adjusted for differences in susceptibility to leptospirosis by age and gender, and adjusts for differences in the population structure between countries. Crude incidence for each of 34 countries with quality-assured data was obtained from the systematic review, by calculating for each country the mean of reported incidences, weighted by the study population. Global mean case fatality was obtained by calculating the mean case fatality among the 35 studies reporting these data (S3 Table), weighted by the study population. All-ages crude mortality was estimated by multiplying the point estimate for crude incidence in each country by the point estimate for global mean case fatality.

To obtain age and gender-specific incidence and mortality for countries that had quality-assured studies, we first characterized the differences in susceptibility to leptospirosis disease or death based on age and gender using the available data. We obtained case proportions within 16 age and gender groups from published and grey literature population-based studies that described case series with this information (S5 Table). These data were used to calculate the mean and standard deviation (SD) for the relative risk for membership in each demographic group among all cases and deaths, weighted by the number of cases or deaths identified by each study (S6 Table, S10 Table Equation 2). In each country, we then multiplied the crude all-ages incidence and mortality by the relative risks for each demographic group to estimate the incidence and mortality in each demographic. This was then adjusted using the 2010 fractional population [[3](#_ENREF_3)] within each age and gender group (S10 Table Equation 3). In this way, we adjusted for risk of leptospirosis case or death by age and gender independent of the population structure of the countries that provided data.

Step 2: Multivariable regression model selection to predict estimated disease incidence and mortality in all countries

In step 1, the mean crude incidence was calculated for each of 34 countries or territories that had available quality-assured data. A log transformation was applied to the mean reported incidence for each country to comply with normality assumptions. These log-transformed values for mean incidence were used as inputs in the model. To construct the regression model, candidate variables were chosen based on the degree of availability of data for all countries and territories, statistically-significant relationship with incidence in univariable regression (p<0.2), and a plausible relationship with leptospirosis incidence. A total of 147 variables (table E, S2 protocol), obtained from Population Reference Bureau ([www.prb.org](http://www.prb.org)), WHO, and UN sources, were screened using these criteria, resulting in the following eight variables that were evaluated for inclusion in a multivariable prediction model:

1. Percent of the population with access to improved water sources (WHO / UNICEF Joint Monitoring Programme for Water Supply and Sanitation, [http://www.wssinfo.org/documents-links/documents/?tx_displaycontroller[type]=country_files](http://www.wssinfo.org/documents-links/documents/?tx_displaycontroller%5Btype%5D=country_files)).
2. Life expectancy at birth (WHO Global Health Observatory Database, <http://apps.who.int/ghodata/>).
3. Gross National Income per capita, PPP-adjusted [log-transformed] (World Bank, <http://data.worldbank.org/indicator/NY.GNP.PCAP.PP.CD>).
4. Percent urbanization (UN Population Division, <http://esa.un.org/wup2009/unup/index.asp?panel=1>).
5. Distance in degrees of latitude from the equator (based on the geographic centroid) (opengeocode.org, http://opengeocode.org/cude/download.php?file=/home/fashions/public_html/opengeocode.org/download/cow.txt)
6. Whether the country is located on a small island (≤65,000 km^2^ in area) with geographic centroid within the Tropics of Cancer and Capricorn.
7. Proportion of studies from each country providing national rather than regional estimates
8. Population density (UN Population Division, <http://esa.un.org/wup2009/unup/index.asp?panel=1>)

We used a best subsets regression approach in order to define the variables used in a multivariable linear regression prediction model. Models were constructed to predict the incidence of leptospirosis using every possible combination of these variables, with or without an intercept term, selecting the combination that produced the highest adjusted R^2^ for the model, i.e. the model with the best predictive power (*regsubsets* command in *leaps* package for R)[[4](#_ENREF_4)]. The subset of variables in the prediction model therefore had the smallest prediction error for the log of crude leptospirosis incidence. A variable representing use of national rather than regional estimates did not enter the final model. No other dummy variables for study quality metrics were used, as very few countries had multiple studies that used both active and passive surveillance methodologies, for example. This model was evaluated by checking the normality, homoscedasticity and independence of residuals. The model is summarized in Equation 4 in S10 Table. Because there were limited raw data available on leptospirosis mortality, with limited representativity (35 studies from 24 countries, S4 Table), we did not construct a different model based on these data, as uncertainty of estimates from such a model would be unacceptably high. Instead, we modelled mortality using the assumption that leptospirosis mortality is largely determined by leptospirosis case fatality. We therefore used the same variables obtained by this process to predict leptospirosis mortality.

Step 3: Use the regression model to estimate age and gender-specific incidence and mortality in all countries

To provide an estimate for incidence and mortality that corrects for the age and gender distribution of each country, we used the regression model to individually estimate a fractional incidence and mortality for each gender in the following age groups: 0-9, 10-19, 20-29, 30-39, 40-49, 50-59, 60-69, 70+. The logarithm of age and gender-specific estimates for incidence and mortality (output from Step 1 above) were entered into the regression (*lm* command, R core package)[[5](#_ENREF_5)] to separately determine the variable coefficients and error terms for each age and gender group, listed in S9 Table (*fit* command, R core package). Using the *predict* command (R core package), the variables and their coefficients were used to predict the log incidence and log mortality (along with respective standard errors) for all country models within each age and gender group, which were then transformed from the log scale (S10 Table Equation 5). A lognormal probability distribution was created, described by model estimates of the mean and standard errors of the incidence and mortality for each age and gender strata.

Step 4: Estimate adjustment factor for the potential impact of under-reporting of cases and death due to laboratory confirmation.

We incorporated incomplete laboratory diagnosis into the estimates. Laboratory confirmation of leptospirosis diagnosis can be technically and logistically difficult, requiring paired sera for complete serodiagnostic evaluation. As a result, relying on laboratory-confirmed cases introduces a significant risk of bias due to under-reporting. Some studies reported data for both clinically suspected and laboratory-confirmed cases and deaths (S7 Table). The mean and 95% CI for the ratio of clinically suspected to laboratory-confirmed cases and deaths were calculated, using weights according to the number of cases or deaths identified in each study (manuscript Fig 2, S10 Table Equation 1). These data were used to construct probability distributions to model the ratio of under-reporting due to incomplete laboratory testing. In this case, normal distributions were used with the mean and standard distribution calculated from the studies reported above.

Step 5: Combine age- and gender-specific predictions to estimate the incidence and mortality in each country, adjusting for bias due to laboratory confirmation.

From the predicted demographic specific incidence and mortality and their standard errors, we used Monte Carlo simulation to generate a stochastic estimate of the actual incidence and mortality. Lognormal probability distributions with mean and standard deviation equivalent to each predicted demographic mean and standard error from Step 1 were used to generate the simulated data sets. Use of the lognormal distribution resulted in the required skewed distribution with a lower bound of zero. To estimate the predicted incidence and mortality, corrected for under reporting, the distributions describing the incidence and mortality estimates were first randomly sampled 1000 times. Incidences were adjusted for under reporting by multiplying each random draw of the incidence by a random draw from a normal distribution of mean 3·1 and standard deviation 1·0. Likewise, mortality was adjusted for underreporting by multiplying each random draw for mortality by a random variable from a normal distribution of mean 2·2 and standard deviation 0·66. These two means and standard deviations for under reporting were as calculated in Step 4.

This resulted in 1000 samples estimating the incidence and mortality, corrected for under-reporting for each demographic group in each country. From these 1000 samples, the mean incidence and mortality was calculated, and the 95% uncertainty limits were calculated from the 2·5% and 97·5% percentiles. Total cases and deaths were calculated using published population data. Monte Carlo simulation was performed using the Poptools plug-in for Microsoft Excel 2007 (http://www.poptools.org). Random samples from the lognormal distribution were calculated using the dLogNormalDev function of Poptools, which uses a variation of the Mersenne Twister algorithm for randomization. By accounting for the potential extent of under-reporting due to the difficulty of laboratory confirmation, this calculation estimates the bounds for the estimated case and death burden of leptospirosis. To reflect the uncertainty of the predictions, sub-regional and global predictions are rounded to three significant digits, with a precision limit of the nearest 100 cases or deaths. Morbidity and mortality in each demographic group are also summarized by sub-region, with cases and deaths reported without rounding.

**Considerations in the modelling approach**

Regression was performed to predict incidence and mortality, using a model selection strategy whose goal was to maximize predictive power based on adjusted R squared. Alternative model selection and validation procedures, such as out-of-sample prediction validity, were not used, as the goal of the model was to provide maximum prediction power, and not necessarily to understand the relationship between these variables and the incidence or mortality of leptospirosis. A linear regression approach was used instead of a Bayesian framework, in order to use the most interpretable model and make the fewest assumptions about the distribution of the data. We used a linear regression model, although generalized linear models, such as the Poisson or the gamma distributions, are designed to model non-negative values. However, Poisson regression yielded a model that was severely over-dispersed and the gamma regression did not converge. The linear model did the best job in fitting the log of the incidence and log of the mortality in each demographic group, but it does present challenges in transforming from the log scale. By modelling log proportional incidence, the resulting proportional incidence is distributed as lognormal and those regressions with larger standard deviations on the log scale result in inflated estimates and standard deviations after exponentiation. Therefore, some specific country estimates had high uncertainty and unexpected values resulting from atypical country characteristics. These countries had inflated estimates due to exponentiation from the log scale, which incorporates the standard error into the estimate. Estimates are therefore more reliable at the regional and global level, and caution should be taken when interpreting individual country estimates.

Almost all studies met the same “medium” quality assessment; however, we did identify sources of heterogeneity due to study quality, principally use of active or passive surveillance. Significantly lower rates of disease were reported by studies using passive surveillance, which suggests that inclusion of lower quality passive surveillance studies may bias towards an underestimate of the global burden. We attempted to adjust for this variation by preferentially using data from active surveillance studies, as we consider them the most reliable study design. However, very few countries had data from both kinds of studies, and so the effect on the estimate was negligible. We also attempted to adjust for study quality in the prediction model by incorporating a dummy variable to represent the use of active or passive study design. However, when added to the model, this variable was not statistically significant. Similarly, a variable representing use of national vs. regional estimates did not enter the final model. Due to the substantial uncertainty in the data, the forced inclusion of these variables would lead to reduced precision of our morbidity and mortality predictions without adding information. As inclusion of the lower quality passive surveillance data tended to bias towards an under-estimate, we preferred to generate a conservative estimate rather than increase the uncertainty in the predictions.

We were unable to model an estimate for mortality directly from the data as only 35 mortality studies were identified, with limited geographic distribution. Therefore, we modelled mortality based on disease incidence and the mean case fatality from the worldwide data. Since information used to calculate case fatality ratio was available from few studies, and from very few world sub-regions, our model used the mean of all available case fatality data, and was not able to use different case fatalities by region based on the raw data. Leptospirosis case fatality varies as a function of several factors, such as healthcare infrastructure, all-cause mortality, and regionally predominant strain. Regional data relating to circulating leptospiral serovar are not available and cannot be modelled. We attempted to identify country-specific or region-specific indicators that could be used to model regional variation in case fatality. However, we could not find any linear relationship between likely health or infrastructure indicators and reported case fatality, suggesting that the regional variability in case fatality may not be easily modelled, which emphasizes the importance of improved surveillance and reporting efforts to reduce this source of uncertainty. We therefore used the global mean case fatality to estimate mortality. For most underdeveloped countries, this case fatality may be an under-estimate, as many deaths due to leptospirosis are unconfirmed due to the difficulties of obtaining laboratory confirmation without paired serology. Therefore, our mortality estimates should be considered conservative. Incidence and mortality for some small island territories could not be estimated because of missing data in the regressor variables or population distribution.

**Appendix: R code**

LeptoBOD.R

Copyright (C) 2012 José E. Hagan and Michael Kane

This program is free software; you can redistribute it and/or

modify it under the terms of the GNU General Public License

as published by the Free Software Foundation; either version 2

of the License, or (at your option) any later version.

This program is distributed in the hope that it will be useful,

but WITHOUT ANY WARRANTY; without even the implied warranty of

MERCHANTABILITY or FITNESS FOR A PARTICULAR PURPOSE. See the

GNU General Public License for more details.

For a copy of the GNU General Public License, write to the Free Software

Foundation, Inc., 51 Franklin Street, Fifth Floor, Boston, MA 02110-1301, USA.

# Model creation

#load data

x <- read.csv("8-2COUNTRIES.csv", as.is=TRUE, na.strings='.', sep=",")

# All Subsets Regression

library(leaps)

attach(x)

leapsnoint<-regsubsets(log(x$mean_inc)~x$tropisl+x$urban_pop_perc+x$combined_le+x$latitude+log(x$gni_ppp),data=x,nbest=1, intercept=FALSE)

leaps<-regsubsets(log(x$mean_inc)~x$tropisl+x$urban_pop_perc+x$combined_le+x$latitude+log(x$gni_ppp),data=x,nbest=1, intercept=TRUE)

# view results

Nointmodels<-summary(leapsnoint)

# plot a table of models showing variables in each model.

# models are ordered by the selection statistic.

plot(leapsnoint,scale="adjr2")

# view results

Intmodels<-summary(leaps)

# plot a table of models showing variables in each model.

# models are ordered by the selection statistic.

plot(leaps,scale="adjr2")

if (max(Nointmodels$adjr2)<max(Intmodels$adjr2)) print(c("The model with the highest adjusted R2 is:",Intmodels$which[which.max(Intmodels$adjr2),])) else print(c("The model with the highest adjusted R2 is:",Nointmodels$which[which.max(Nointmodels$adjr2),]))

#---------------------------Predictions for morbidity

#munging: standardize nomenclature of population data

library(reshape)

x <- rename(x, c(popm0_9="popm_0_9"))

x <- rename(x, c(popf0_9="popf_0_9"))

#regressors selected using best subsets regression

regressors <- c("tropisl", "urban_pop_perc", "latitude", "combined_le")

#mean incidence relative risk extracted from 10 studies with appropriately stratified data in 16 demographic groups

RRF0=0.0305655

RRF1=0.245776217

RRF2=0.498731327

RRF3=0.60902427

RRF4=0.538121844

RRF5=0.60291652

RRF6=0.657130804

RRF7=0.341145194

RRM0=0.254342231

RRM1=1.392323868

RRM2=2.380246841

RRM3=2.183009924

RRM4=1.929800279

RRM5=1.875261921

RRM6=1.538035422

RRM7=1.010509744

# Estimation of demographic incidence based on crude country incidence (x$mean_inc). Method uses incidence ratio

# to ensure that incidence distribution among demographics maintains the same proportions, reflecting

# differences in demographic susceptibility

#1) multiply mean incidence by rr to get adjusted (not normalized) incidences

x$lffc0.1=x$mean_inc*RRF0

x$lffc1.1=x$mean_inc*RRF1

x$lffc2.1=x$mean_inc*RRF2

x$lffc3.1=x$mean_inc*RRF3

x$lffc4.1=x$mean_inc*RRF4

x$lffc5.1=x$mean_inc*RRF5

x$lffc6.1=x$mean_inc*RRF6

x$lffc7.1=x$mean_inc*RRF7

x$lmfc0.1=x$mean_inc*RRM0

x$lmfc1.1=x$mean_inc*RRM1

x$lmfc2.1=x$mean_inc*RRM2

x$lmfc3.1=x$mean_inc*RRM3

x$lmfc4.1=x$mean_inc*RRM4

x$lmfc5.1=x$mean_inc*RRM5

x$lmfc6.1=x$mean_inc*RRM6

x$lmfc7.1=x$mean_inc*RRM7

#2) estimated adjusted (not normalized) cases in each demographic

x$lffc0.2=x$lffc0.1*x$popf_0_9

x$lffc1.2=x$lffc1.1*x$popf_10_19

x$lffc2.2=x$lffc2.1*x$popf_20_29

x$lffc3.2=x$lffc3.1*x$popf_30_39

x$lffc4.2=x$lffc4.1*x$popf_40_49

x$lffc5.2=x$lffc5.1*x$popf_50_59

x$lffc6.2=x$lffc6.1*x$popf_60_69

x$lffc7.2=x$lffc7.1*x$popf_70_

x$lmfc0.2=x$lmfc0.1*x$popm_0_9

x$lmfc1.2=x$lmfc1.1*x$popm_10_19

x$lmfc2.2=x$lmfc2.1*x$popm_20_29

x$lmfc3.2=x$lmfc3.1*x$popm_30_39

x$lmfc4.2=x$lmfc4.1*x$popm_40_49

x$lmfc5.2=x$lmfc5.1*x$popm_50_59

x$lmfc6.2=x$lmfc6.1*x$popm_60_69

x$lmfc7.2=x$lmfc7.1*x$popm_70_

#3) adjusted gross total incidence before normalization: sum adjusted cases/total pop

x$adj_inc=(x$lffc0.2+x$lffc1.2+x$lffc2.2+x$lffc3.2+x$lffc4.2+x$lffc5.2+x$lffc6.2+x$lffc7.2+x$lmfc0.2+x$lmfc1.2+x$lmfc2.2+x$lmfc3.2+x$lmfc4.2+x$lmfc5.2+x$lmfc6.2+x$lmfc7.2)/x$pop*1000

#4) calculate normalization factor as ratio of original incidence and adjusted incidence

x$norm_factor=x$mean_inc/x$adj_inc

#5) normalize demographic-specific incidence and take the log, to enter into the regression.

x$lffc0=log(x$lffc0.1*x$norm_factor)

x$lffc1=log(x$lffc1.1*x$norm_factor)

x$lffc2=log(x$lffc2.1*x$norm_factor)

x$lffc3=log(x$lffc3.1*x$norm_factor)

x$lffc4=log(x$lffc4.1*x$norm_factor)

x$lffc5=log(x$lffc5.1*x$norm_factor)

x$lffc6=log(x$lffc6.1*x$norm_factor)

x$lffc7=log(x$lffc7.1*x$norm_factor)

x$lmfc0=log(x$lmfc0.1*x$norm_factor)

x$lmfc1=log(x$lmfc1.1*x$norm_factor)

x$lmfc2=log(x$lmfc2.1*x$norm_factor)

x$lmfc3=log(x$lmfc3.1*x$norm_factor)

x$lmfc4=log(x$lmfc4.1*x$norm_factor)

x$lmfc5=log(x$lmfc5.1*x$norm_factor)

x$lmfc6=log(x$lmfc6.1*x$norm_factor)

x$lmfc7=log(x$lmfc7.1*x$norm_factor)

#log(mean_inc) ~ regressors.

predVars <- names(x)[grep("^l[mf]fc[0-9]$", names(x), perl=TRUE)]

popVars <- c(

names(x)[grep("^popf_*", names(x), perl=TRUE)],

names(x)[grep("^popm_*", names(x), perl=TRUE)])

demoData <- NULL

for (i in 1:length(predVars)) {

f <- as.formula(paste(predVars[i], "~", paste(regressors, collapse="+"),

"-1"))

xs <- x[,c(predVars[i], "country", "countryno", popVars[i], regressors)]

fit <- lm(f, data=xs)

print(f)

print(summary(fit))

p <- predict(fit, xs, se.fit=TRUE)

xs$rawIncPred <- as.numeric(p$fit)

xs$rawIncSe <- as.numeric(p$se.fit)

df <- xs[,-which(names(xs)==predVars[i])]

df$dpop <- xs[,popVars[i]]

df$predInc <- exp(df$rawIncPred + (df$rawIncSe^2)/2)

df$seInc <- sqrt((exp(df$rawIncSe^2)-1)*exp(2*df$rawIncPred+df$rawIncSe^2))

demoData <- rbind(demoData, df[,-c(which(names(df) == popVars[i]))])

}

demoData$totalPop <- NA

for (country in unique(demoData$country)) {

demoData$totalPop[demoData$country==country] <-

sum(demoData$dpop[demoData$country==country])

}

for (country in demoData$country) {

demoData$demo[demoData$country == country] <- predVars

}

# Let's write out the prediction and prediction errors.

outpath<-"//Output//"

write.csv(demoData, paste (outpath,"RR_DemographicIncidence",Sys.Date(),".csv", sep = "", collapse = NULL), row.names=FALSE)

# Now, let's do the predictions for the unique countries.

countryPred <- data.frame(country=unique(demoData$country),

predCases=NA, seCases=NA, predInc=NA, seInc=NA)

for (country in countryPred$country) {

xs <- demoData[demoData$country==country,]

if (NA %in% xs$predInc) {

warning(paste("NA's found in predictions for", country))

}

predInc <- sum( xs$predInc * xs$dpop, na.rm=TRUE ) / xs$totalPop[1]

seInc <- sum( xs$seInc *xs$dpop, na.rm=TRUE ) / xs$totalPop[1]

predCases <- sum(xs$predInc * xs$dpop / 100, na.rm=TRUE)

seCases <- sum(xs$seInc * xs$dpop / 100, na.rm=TRUE)

countryPred[countryPred$country==country,

c("predCases", "seCases", "predInc", "seInc")] <- c(predCases, seCases,

predInc, seInc)

}

countryPred <- na.omit(countryPred)

countryPred <- countryPred[countryPred$predInc > 0,]

write.csv(countryPred, paste (outpath,"RR_CountryIncidence",Sys.Date(),".csv", sep = "", collapse = NULL), row.names=FALSE)

#-------------------Predictions for mortality

#mean mortality relative risk from 3 studies

RRF0=0.148650264

RRF1=0.251252346

RRF2=0.136132593

RRF3=0.077009453

RRF4=0.628807628

RRF5=0.604122767

RRF6=0.596402239

RRF7=0.798050044

RRM0=0.465384175

RRM1=0.39617704

RRM2=1.114304596

RRM3=1.17315812

RRM4=3.406103026

RRM5=3.682635249

RRM6=3.138355814

RRM7=2.370967556

#Global mean case fatality

crf <- 0.06847

# Estimation of demographic incidence based on crude country incidence. Method uses incidence ratio

# to ensure that incidence distribution among demographics maintains the same proportions, reflecting

# differences in demographic susceptibility

#using proportional cases and deaths

# Estimation of demographic incidence based on crude country incidence. Method uses incidence ratio

# to ensure that incidence distribution among demographics maintains the same proportions, reflecting

# differences in susceptibility due to age and gender.

#1) multiply mean incidence by rr to get adjusted (not normalized) incidences, multiply by case fatality to get deaths

x$lffc0.1=x$mean_inc*RRF0*crf

x$lffc1.1=x$mean_inc*RRF1*crf

x$lffc2.1=x$mean_inc*RRF2*crf

x$lffc3.1=x$mean_inc*RRF3*crf

x$lffc4.1=x$mean_inc*RRF4*crf

x$lffc5.1=x$mean_inc*RRF5*crf

x$lffc6.1=x$mean_inc*RRF6*crf

x$lffc7.1=x$mean_inc*RRF7*crf

x$lmfc0.1=x$mean_inc*RRM0*crf

x$lmfc1.1=x$mean_inc*RRM1*crf

x$lmfc2.1=x$mean_inc*RRM2*crf

x$lmfc3.1=x$mean_inc*RRM3*crf

x$lmfc4.1=x$mean_inc*RRM4*crf

x$lmfc5.1=x$mean_inc*RRM5*crf

x$lmfc6.1=x$mean_inc*RRM6*crf

x$lmfc7.1=x$mean_inc*RRM7*crf

#2) estimated adjusted (not normalized) deaths in each demographic

x$lffc0.2=x$lffc0.1*x$popf_0_9

x$lffc1.2=x$lffc1.1*x$popf_10_19

x$lffc2.2=x$lffc2.1*x$popf_20_29

x$lffc3.2=x$lffc3.1*x$popf_30_39

x$lffc4.2=x$lffc4.1*x$popf_40_49

x$lffc5.2=x$lffc5.1*x$popf_50_59

x$lffc6.2=x$lffc6.1*x$popf_60_69

x$lffc7.2=x$lffc7.1*x$popf_70_

x$lmfc0.2=x$lmfc0.1*x$popm_0_9

x$lmfc1.2=x$lmfc1.1*x$popm_10_19

x$lmfc2.2=x$lmfc2.1*x$popm_20_29

x$lmfc3.2=x$lmfc3.1*x$popm_30_39

x$lmfc4.2=x$lmfc4.1*x$popm_40_49

x$lmfc5.2=x$lmfc5.1*x$popm_50_59

x$lmfc6.2=x$lmfc6.1*x$popm_60_69

x$lmfc7.2=x$lmfc7.1*x$popm_70_

#3) adjusted gross total mortality before normalization: sum adjusted cases/total pop

x$adj_mort=(x$lffc0.2+x$lffc1.2+x$lffc2.2+x$lffc3.2+x$lffc4.2+x$lffc5.2+x$lffc6.2+x$lffc7.2+x$lmfc0.2+x$lmfc1.2+x$lmfc2.2+x$lmfc3.2+x$lmfc4.2+x$lmfc5.2+x$lmfc6.2+x$lmfc7.2)/x$pop*1000

#4) calculate normalization factor as ratio of original mortality and adjusted incidence

x$norm_factor=(x$mean_inc*crf)/x$adj_mort

#5) normalize demographic-specific mortality and take the log, to enter into the regression.

x$lffc0=log(x$lffc0.1*x$norm_factor)

x$lffc1=log(x$lffc1.1*x$norm_factor)

x$lffc2=log(x$lffc2.1*x$norm_factor)

x$lffc3=log(x$lffc3.1*x$norm_factor)

x$lffc4=log(x$lffc4.1*x$norm_factor)

x$lffc5=log(x$lffc5.1*x$norm_factor)

x$lffc6=log(x$lffc6.1*x$norm_factor)

x$lffc7=log(x$lffc7.1*x$norm_factor)

x$lmfc0=log(x$lmfc0.1*x$norm_factor)

x$lmfc1=log(x$lmfc1.1*x$norm_factor)

x$lmfc2=log(x$lmfc2.1*x$norm_factor)

x$lmfc3=log(x$lmfc3.1*x$norm_factor)

x$lmfc4=log(x$lmfc4.1*x$norm_factor)

x$lmfc5=log(x$lmfc5.1*x$norm_factor)

x$lmfc6=log(x$lmfc6.1*x$norm_factor)

x$lmfc7=log(x$lmfc7.1*x$norm_factor)

#log(mean_inc) ~ regressors.

predVars <- names(x)[grep("^l[mf]fc[0-9]$", names(x), perl=TRUE)]

popVars <- c(

names(x)[grep("^popf_*", names(x), perl=TRUE)],

names(x)[grep("^popm_*", names(x), perl=TRUE)])

demoData <- NULL

for (i in 1:length(predVars)) {

f <- as.formula(paste(predVars[i], "~", paste(regressors, collapse="+"),

"-1"))

xs <- x[,c(predVars[i], "country", "countryno", popVars[i], regressors)]

fit <- lm(f, data=xs)

print(f)

print(summary(fit))

p <- predict(fit, xs, se.fit=TRUE)

xs$rawmortPred <- as.numeric(p$fit)

xs$rawmortSe <- as.numeric(p$se.fit)

df <- xs[,-which(names(xs)==predVars[i])]

df$dpop <- xs[,popVars[i]]

df$predmort <- exp(df$rawmortPred + (df$rawmortSe^2)/2)

df$semort <- sqrt((exp(df$rawmortSe^2)-1)*exp(2*df$rawmortPred+df$rawmortSe^2))

demoData <- rbind(demoData, df[,-c(which(names(df) == popVars[i]))])

}

demoData$totalPop <- NA

for (country in unique(demoData$country)) {

demoData$totalPop[demoData$country==country] <-

sum(demoData$dpop[demoData$country==country])

}

for (country in demoData$country) {

demoData$demo[demoData$country == country] <- predVars

}

# Let's write out the prediction and prediction errors.

outpath<-"//Output//"

write.csv(demoData, paste (outpath,"RR_DemographicMortality",Sys.Date(),".csv", sep = "", collapse = NULL), row.names=FALSE)

# Now, let's do the predictions for the unique countries.

countryPred <- data.frame(country=unique(demoData$country),

preddeaths=NA, sedeaths=NA, predmort=NA, semort=NA)

for (country in countryPred$country) {

xs <- demoData[demoData$country==country,]

if (NA %in% xs$pred) {

warning(paste("NA's found in predictions for", country))

}

predmort <- sum( xs$predmort * xs$dpop, na.rm=TRUE ) / xs$totalPop[1]

semort <- sum( xs$semort *xs$dpop, na.rm=TRUE ) / xs$totalPop[1]

preddeaths <- sum(xs$predmort * xs$dpop /100, na.rm=TRUE)

sedeaths <- sum(xs$semort * xs$dpop /100, na.rm=TRUE)

countryPred[countryPred$country==country,

c("preddeaths", "sedeaths", "predmort", "semort")] <- c(preddeaths, sedeaths, predmort, semort)

}

countryPred <- na.omit(countryPred)

countryPred <- countryPred[countryPred$predmort > 0,]

write.csv(countryPred, paste (outpath,"RR_CountryMortality",Sys.Date(),".csv", sep = "", collapse = NULL), row.names=FALSE)

**References**

1. Mathers CD, Ezzati M, Lopez AD. Measuring the burden of neglected tropical diseases: the global burden of disease framework. PLoS Negl Trop Dis. 2007; 1: e114. doi: 10.1371/journal.pntd.0000114 PMID: 18060077

2. World Health Organization. List of Member States by WHO region and mortality stratum. World Health Report 2003. 2003: 182

3. Division UNP. 2010 Population Estimates. World Urbanization Prospects: The 2009 Revision Population Database. 2010; [accessed 2011 Mar 21];Available from: <http://esa.un.org/wup2009/unup/index.asp?panel=1:>

4. Lumley T, Miller A (2009) Leaps: regression subset selection.

5. Team RC (2012) R: A language and environment for statistical computing. Vienna, Austria: R Foundation for Statistical Computing.
