# Supplementary material for: Global Morbidity and Mortality of Leptospirosis: A Systematic Review
Source: PLoS Negl Trop Dis. 2015 Sep 17;9(9):e0003898. doi: 10.1371/journal.pntd.0003898 (PMC4574773; doi:10.1371/journal.pntd.0003898)
Supplement: S2 Protocol — (DOCX) [file pntd.0003898.s003.docx]

## S2 Protocol: Manual of definitions

## A: Variables evaluated during initial screening of reports

| **LERG dictionary for identification of data.** | | | | |
| --- | --- | --- | --- | --- |
| **No.** | **Variable name** | **Variable** | **Description** | **Coding** |
| 1 | REFNO | Reference number | Reference number in LERG database | Nos. 1 - 12,025 |
| 2 | REFEN | Reference Number in EndNote library | Reference Number in EndNote library | 999999 To enter in EndNote |
| 3 | DB | Database source | Database source for the reference | MED: Medline  POP: Popline  CAB: CAB Abstracts  CIN: CINAHL  ECO: Econlit  EMB: Embase  BA: Biological abstracts  PAIS: PAIS International  PRO: ProMED  ISI: ISI Web of Knowledge  IMEMR: IND MED East Med. Region  WPRIM: W Pacific Region IND MED  LIL: LILACS  BIR: BIREME  KOR: Koreamed  AFR: African HealthLine  HEL: HELLIS Network Libraries  HRD: Health Research Dev Inf Net  AMI: Amicus Canadian Union Cat  ITM: Inst Trop Med Belgium  JAP: Japan Sci Techn Inf Aggregator  BDSP: L’ecole Nat Sante Pub  BST: Bibliothèque de Santé Tropicale  TUR: Turkish Medline  DIR: Dir Pub Conf Proceeds  CUI: Cuidem  WHOL: WHOLIST  CCT: Cochrane CT  BL: Bioline  IMED: IndMed-Indian Medlars Centre  AJOL: African Journals on Line  AIM: African Index Medicus  AFR: Afro Library  9999: To confirm |
| 4 | DBNO | Source ID number | Identification number in source database | 999999: Not available |
| 5 | PUB | Publication year | Year of publication | 1945-2008 |
| 6 | REF | Reference | Journal or book name, issue and page number.  Note: journals articles from database BA doesn’t have page number | 9999: Not available |
| 7 | AUT | Author | Last name and initials of all authors  Note: ISI database only provides data for the first author | 9999: Not available |
| 8 | TIT | Title | Full title | 9999: Not available |
| 9 | ABS | Abstract available | Abstract available from database source | 0: No  1: Yes |
| 10 | ABST | Abstract text | Full text of abstract  Note: ISI database doesn’t have any abstracts | 8888: Not available |
| 11 | TYP | Type of report | Journal Article, Book, Book Section, Conference Proceedings, Generic, Patent, Report, Letters, Thesis and ProMED | 9999: Undetermined  AP: Abstract of Published Item  BC: Book Chapter  BI: Bibliography  BIO: Biography  BO: Book  BS: Book Section  BU: Bulletins  CM Comments  COR: Corrections  CP: Conference Proceedings  ED: Editorials  ER: Erratum  GE: Generic  IN: Interview  JA: Journal Article  LT: Letters  MA : Meeting Abstracts  NE: News  NO: Note  PA: Patent  PM: ProMED  RE: Report  RP: Reprints  RW: Reviews  SS: Short Survey  TH: Thesis |
| 12 | LA | Language | Language on the full text article | Afr: Afrikaans  Ara: Arabic  Bos: Bosnian  Bul: Bulgarian  Chi: Chinese  Cro: Croatian  Cze: Czech  Dan: Danish  Dut: Dutch  Fin: Finnish  Fre: French  Ger: Georgian  Gre: Greek, Modern  Heb: Hebrew  Hun: Hungarian  Indon: Indonesian  Ita: Italian  Jpn: Japanese  Kor: Korean  Lit: Lithuanian  Mul: Multiple languages  Net: Netherlandish  Nor: Norwegian  Per: Persian  Pol: Polish  Por: Portuguese  Rum: Romanian  Rus: Russian  Scr: Serbo-Croatian  Ser: Serbian  Slo: Slovak  Spa: Spanish  Swe: Swedish  Thai: Thai  Tur: Turkish  Ukr: Ukrainian  Viet: Vietnamese  9999: Undetermined |
| 13 | LAREAD | Languages read | References in English, French, Italian, Spanish and Portuguese. | 1: English, French, Italian, Spanish and Portuguese.  0: Other languages  9: Undetermined |
| 14 | DUP | Duplicate reference | Duplicate reference in database  Note: When two or more reports from PRO contain the same data were considered as duplicated those reports with less information. | 0: No  1: Yes |

| **LERG dictionary for initial screening of data.** | | | | |
| --- | --- | --- | --- | --- |
| Identification variables 1 to 14 from the Appendix 3A  Screening variables 15 to 24 from the Appendix 3A | | | | |
| 15 | LEP | Leptospirosis | Reference on the subject of leptospirosis. Review of title and abstract was performed and full text when it was available. | 0: Not on leptospirosis  1: Yes  2: Unclassifiable on leptospirosis subject based on title and/or abstract  3: Unclassifiable based on title alone  8: Not applicable (for duplicated ref.) |
| 16 | HUM | Human Leptospirosis | Reports on human cases of leptospirosis or *Leptospira* infection in a defined patient or subject population. Review of title and abstract was performed and full text when it was available. | 0: No  1: Yes  8: Not applicable  9: Abstract not available to classify |
| 17 | ANI | Animal Leptospirosis | Reports on cases of leptospirosis in animals or Leptospirosis infection in animal populations, outside of the experimental setting. Review of title and abstract was performed and full text when it was available. | 0: No  1: Yes  8: Not applicable  9: Abstract not available to classify |
| 18 | OTH | Others reports | Reports referring to leptospirosis not included in 14 and 15. Review of title and abstract was performed and full text when it was available. | 0: No  1: Yes  8: Not applicable  9: Abstract not available to classify |
| 19 | REV | Review | Reviews on leptospirosis and *Leptospira* infection which present information from published reports. If review contains previously non-published data, the reference will be coded as a review and as a report on human leptospirosis (HUM). Review of title and abstract was performed and full text when it was available. | 0: No  1: Yes  8: Not applicable  9: Abstract not available to classify |
| 20 | NOD | No data | Literature reviews, letters and other reports on human leptospirosis that do not contain data. Review of title and abstract was performed and full text when it was available. | 0: No  1: Yes  8: Not applicable  9: Abstract not available to classify |
| 21 | FYR | Initial Year | First year of study on human leptospirosis or *Leptospira* infection. Review of title and abstract was performed and full text when it was available. | 8888: Not applicable  9999: Unknown |
| 22 | LYR | Final Year | Last year of study on human leptospirosis or *Leptospira* infection. Review of title and abstract was performed and full text when it was available. | 8888: Not applicable  9999: Unknown |
| 23 | INC | Incidence | Reports in which was stated incidence rates, or defined population base, or defined geographic region in title and/or abstract and/or full text. | 0: No  1: Yes  8: Not applicable  9: Unknown |
| 24 | PATNO | No. of study patients | 1. Number of patients with leptospirosis, whether suspected or confirmed, who had symptomatic disease  2. Cases were identified within a defined study population.  3. If both suspected and confirmed cases are reported, the number of suspected cases will be entered. | 8888: Not applicable  9999: Unknown |

## B: Evaluation of disease incidence reports.

| **LERG dictionary. Inclusion criteria for incidence reports.** | | | | |
| --- | --- | --- | --- | --- |
| STEP 2A | | | | |
| Identification variables 1 to 14 from the Appendix 1A  Screening variables 15 to 24 from the Appendix 1B  Inclusion criteria for incidence study 25 to 32 from the Appendix 1C.1 | | | | |
| 25 | NRIS | National representative incidence reports | A study that systematically ascertains cases of leptospirosis that occur in a defined population or set of populations, designed to obtain nationally representative estimates of disease incidence.  • Designed to identify all cases of leptospirosis which occur at the community level during the study period  • Uses active case ascertainment protocols such as community-based case finding or outpatient surveillance  • Has a defined population base as determined by investigator-initiated surveys or census  • Case ascertainment performed as a systematic or continuous process during the study period. | 0: No  1: Yes  8: Not applicable  9: Unknown |
| 26 | CBIS | Community based incidence study | A study that systematically ascertains cases of leptospirosis that occur in a defined population, designed to obtain estimates of disease incidence that are representative of a specified community or set of communities.  • Designed to capture all cases of leptospirosis which occur at the community level during the study period  • Uses active case ascertainment protocols such as community-based case finding or outpatient surveillance  • Has a defined population base as determined by investigator-initiated surveys or census  • Case ascertainment performed as a systematic or continuous process during the study period. | 0: No  1: Yes  8: Not applicable  9: Unknown |
| 27 | LCS | Large cohort studies | A defined population for which subjects are individually recruited and followed over time with the aim of identifying leptospirosis cases and estimating disease incidence for the cohort population.  • May include cohorts of high risk populations (i.e., rural subsistence farmers, urban slum dwellers, occupational risk groups).  • Evaluates disease incidence in enrolled cohort subjects who are representative of the population to be studied.  • Case ascertainment performed as a systematic or continuous process during the study period. | 0: No  1: Yes  8: Not applicable  9: Unknown |
| 28 | NRCSS | National, regional, or community-based surveillance study | • Reports which incorporate a continuous and systematic process for identifying cases of leptospirosis  • Studies performed on the national level or for a defined regional entity or community.  • The study has a defined population base as determined by investigator-initiated surveys or census or reports a defined geographical region for which census information is likely to be obtained.  • Reports that relies on active case ascertainment at hospital facilities or laboratories.  • Reports that relies on passive reporting of cases that is initiated by healthcare providers and/or laboratory staff.  Note: For the purpose of this systematic review on disease burden, active population-based studies will be defined as surveillance reports if case finding protocols were performed exclusively at hospital healthcare facilities or laboratories. Reports which perform active community-based surveillance at outpatient healthcare facilities will be defined as national or community-based disease incidence reports if they fulfill the criteria for these reports (variables NRIS and CBIS). | 0: No  1: Yes  8: Not applicable  9: Unknown |
| 29 | POP | Population base | Describe the population base for which the study was performed (i.e. national census). | 0: No  1: Yes  8: Not applicable  9: Unknown |
| 30 | GEOP | Geographical setting | Area within the country was the study was performed and for which an estimate of the inhabiting population was not described but is likely to be obtained or by national census. | 8:Not applicable  9: Unknown |
| 31 | 1970DI | Incidence rates after 1/1/1970. | 1. Describe information on cases and disease incidence rates which occur after 1/1/1970.  2. If reports have information for periods prior to and after this date, these reports will be classified as “1” if disaggregated rates for the period after 1/1/1970 were reported or can be calculated. | 0: No  1: Yes  8: Not applicable  9: Unknown |
| 32 | GRAL | Disease incidence inclusion criteria general evaluation | Describes the final assessment evaluation of each disease incidence report. | 0: Do not fullfils the inclusion criteria for incidence studies.  1: Fullifls the inclusion criteria for incidences studies |
| 33 | PDF | Full text | Reports in which full text is available | 0: No  1: Yes  8: Not applicable |

| **Quality assessment for disease incidence reports.** | | | | | |
| --- | --- | --- | --- | --- | --- |
| **No.** | **Variable name** | **Variable** | **Description** | | **Coding** |
| 1 | REFNO | Reference number | Reference number in LERG database | Nos. 1 - 11,462 | |
| 2 | PUB | Publication year | Year of publication | 1945-2008 | |
| 3 | REF | Reference | Journal or book name, issue and page number.  Note: journal articles from database BA doesn’t have a page number | 9999: Not available | |
| 4 | AUT | Author | Last name and initials of all authors  Note: ISI database only provides data for the first author | 9999: Not available | |
| 5 | TIT | Title | Full title | 9999: Not available | |
| 6 | ABST | Abstract text | Full text of abstract  Note: ISI database doesn’t have abstract | 8888: Not available | |
| SPECIFIC VARIABLES FOR QUALITY ASSESSMENT | | | | | |
| 7 | POP | Population base | Study population was representative of the population base for the study setting. Reports were classified as being population-based if there was evidence in the method section that 1) a population base was identified and 2) case ascertainment was performed for the entire population base, either by a network of health care or reference laboratory sites. | 0: No  1: Yes  8: Not applicable  9: Unknown | |
| 8 | GEOP | Geographic region | 1. Defined geographical region in which the study was performed.  2. An estimate of the inhabiting population was not described but an estimate is likely to be obtained.  3. Case ascertainment was performed for the entire geographical region. | 0: No  1: Yes  8: Not applicable  9: Unknown | |
| 9 | RELIP | Population base reliably and recently estimated | 1. The population base was determined by the investigator-initiated survey or census.  2. The population base was performed in the last 10 years (considering the date when the study was performed) | 0: No  1: Yes, was reliable estimated  2: Yes, was recently estimated  3: Yes, was recently and reliable estimated  8: Not applicable  9: Unknown | |
| 10 | LCC | LERG laboratory case confirmation | Were used standard diagnostic methods and criteria according to LERG recommendations  1) 4 fold increase in MAT titre in acute and convalescent serum samples, 2) single MAT ≥ 1:400, 3) seroconversion, 4) isolation of *Leptospira spp* from normally sterile site, 5) detection of *Leptospira spp* in clinical samples by histological/ histochemical/ immunostaining techniques, and 6) *Leptospira* DNA detected by PCR. | 0: No  1: Yes  8: Not applicable  9: Unknown | |
| 11 | ACT | Active case ascertainment | Active case ascertainment whether community or hospital/provider-based | 0: No  1: Yes  8: Not applicable  9: Unknown | |
| 12 | IRAT | Incidence rates | Incidence rates were calculated by year or can be extrapolated from the data? | 0: No  1: Yes  8: Not applicable  9: Unknown | |
| 13 | DUR | Study period | Study duration ≥ 1 year. | 0: No  1: Yes  8: Not applicable  9: Unknown | |
| 14 | LPC | LERG laboratory probable case | Probable case:  1) presence of IgM antibodies by ELISA or dipstick  2) presence of IgM/IgA antibodies in the immunofluorescence assay.  3) MAT titre ≥1:100 in single acute-phase serum sample. | 0: No  1: Yes  8: Not applicable  9: Unknown | |
| 15 | LAB | Laboratory confirmation | Laboratory confirmation was performed. | 0: No  1: Yes  8: Not applicable  9: Unknown | |
| 16 | ASC | Case ascertainment | Case ascertainment community or hospital based | 1: Community based  2: Hospital based  8: Not applicable  9: Unknown | |
| 17 | CH | Changes in case ascertainment | Changes in case ascertainment during the study period | 0: No  1: Yes  8: Not applicable  9: Unknown | |
| 18 | BIAS | Paired sample | Proportion of subjects for whom paired and single samples were evaluated during laboratory confirmation | 8888: Not applicable  9999: Unknown | |
| 19 | DOTS | Drop Outs | Proportion of drop outs during the follow up.  Note: only applicable for cohort studies (including RCTs) | 0: No  1: Yes  8: Not applicable  9: Unknown | |
| 20 | AGE | Age rate | Age specific attack rates calculated or can be extrapolated | 0: No  1: Yes  8: Not applicable  9: Unknown | |
| 21 | SEX | Sex rate | Sex specific attack rates calculated or can be extrapolated | 0: No  1: Yes  8: Not applicable  9: Unknown | |
| 22 | IRATME | Median incidence rates | Incidence rates were calculated for a period of time (more than one year) and cannot be stratified by year. | 0: No  1: Yes  8: Not applicable  9: Unknown | |
| 23 | GRAL | Incidence studies general quality assessment evaluation | Describes the final assessment of quality criteria evaluation of each disease incidence report. | 0: Low quality of evidence  1: Medium quality of evidence  2: High quality of evidence | |

**C: Evaluation of disease sequelae reports.**

| **LERG dictionary. Inclusion criteria for sequelae reports.** | | | | | | | | |
| --- | --- | --- | --- | --- | --- | --- | --- | --- |
| STEP 2B | | | | | | | | |
| Identification variables 1 to 14 from the Appendix 1A  Screening variables 15 to 24 from the Appendix 1B  Inclusion criteria for incidence study 25 to 30 from the Appendix 1C.2 | | | | | | | | |
| 25 | LFUS | Longitudinal follow-up studies | | A study in which a population of suspected and/or confirmed cases of leptospirosis are:  • Individually and systematically recruited at the time of identification with the illness.  • Cases are followed over time.  • Cases are followed over time with the aim of identifying sequelae and case-fatality for this patient cohort. | | | | 0: No  1: Yes  8: Not applicable  9: Unknown |
| 26 | RS | Retrospective studies | | A study in which:  • A population of suspected and/or confirmed cases of leptospirosis is identified.  • Case are retrospectively identified according to patient registers or notification records.  • Review of historical medical records is performed with the aim of ascertaining cases of leptospirosis.  • Cases are followed over time so as disease sequelae and case-fatality for this patient cohort can be calculated. | | | | 0: No  1: Yes  8: Not applicable  9: Unknown |
| 27 | NRSS | National or regional surveillance studies | | A study in which:  • Suspected and/or confirmed cases of leptospirosis are identified  • Cases are identified during implementation of active or passive surveillance.  • A defined population and geographic region is defined.  • Information on sequelae and case fatality are obtained from information collected as part of surveillance protocol. | | | | 0: No  1: Yes  8: Not applicable  9: Unknown |
| 28 | REPR | Representativeness | | • Suspected cases of leptospirosis are representative of all cases likely to be identified at health care facilities  • Cases are representative for the population and geographical region which the study is conducted. | | | | 0: No  1: Yes  8: Not applicable  9: Unknown |
| 29 | 1970DS | Sequelae rates after 1/1/1970. | | 1. Describe information on cases and disease sequelae rates which occur after 1/1/1970.  2. If reports have information for periods prior to and after this date, these reports will be classified as “1” if disaggregated rates for the period after 1/1/1970 were reported or can be calculated. | | | | 0: No  1: Yes  8: Not applicable  9: Unknown |
| 30 | GRAL | Disease sequelae inclusion criteria general evaluation | | Describes the final assessment evaluation inclusion criteria for each disease sequelae report. | | | | 0: Do not fullfils the inclusion criteria for sequelae studies.  1: Fullifls the inclusion criteria for sequelae studies |
| 31 | PDF | Full text | | Reports in which full text is available | | | | 0: No  1: Yes  8: Not applicable |
| **Quality assessment for disease sequelae reports.** | | | | | | | | |
| **No.** | **Variable name** | | **Variable** | | **Description** | | **Coding** | |
| SPECIFIC VARIABLES FOR REPORT IDENTIFICATION | | | | | | | | |
| 1 | REFNO | | Reference number | | Reference number in LERG database | Nos. 1 - 11,462 | | |
| 2 | PUB | | Publication year | | Year of publication | 1945-2008 | | |
| 3 | REF | | Reference | | Journal or book name, issue and page number.  Note: journals articles from database BA hasn’t page number | 9999: Not available | | |
| 4 | AUT | | Author | | Last name and initials of all authors  Note: ISI database only provides data to first author | 9999: Not available | | |
| 5 | TIT | | Title | | Full title | 9999: Not available | | |
| 6 | ABST | | Abstract text | | Full text of abstract  Note: ISI database hasn’t any abstracts | 8888: Not available | | |
| SPECIFIC VARIABLES FOR QUALITY ASSESSMENT | | | | | | | | |
| 7 | LAB | | Laboratory confirmation | | Laboratory confirmation was performed. | 0: No  1: Yes  8: Not applicable  9: Unknown | | |
| 8 | LCC | | LERG laboratory case confirmation | | Confirmed case:  1) 4 fold increase in MAT titre in acute and convalescent serum samples, 2) single MAT ≥ 1:800, 3) seroconversion, 4) isolation of *Leptospira spp* from normally sterile site, 5) detection of *Leptospira spp* in clinical samples by histological/ histochemical/ immunostaining techniques, and 6) *Leptospira* DNA detected by PCR. | 0: No  1: Yes  8: Not applicable  9: Unknown | | |
| 9 | LPC | | LERG laboratory probable case | | Probable case: 1) presence of IgM antibodies by ELISA or dipstick, and 2) presence of IgM/IgA antibodies in the immunofluorescence assay. | 0: No  1: Yes  8: Not applicable  9: Unknown | | |
| 10 | REP | | Representativeness | | Cases representative of the patient population in the study setting (i.e. consecutive cases enrolled) | 0: No  1: Yes  8: Not applicable  9: Unknown | | |
| 11 | SEQP | | Prospective evaluation of sequelae | | Sequelae identified prospectively during clinical evaluations performed while under medical care for the illness. | 0: No  1: Yes  8: Not applicable  9: Unknown | | |
| 12 | SEQR | | Retrospective evaluation of Sequelae | | Sequelae identified retrospectively during clinical care for the illness. | 0: No  1: Yes  8: Not applicable  9: Unknown | | |
| 13 | SRAT | | Sequelae rates | | Sequelae rates calculated or can be extrapolated from the data | 0: No  1: Yes  8: Not applicable  9: Unknown | | |
| 14 | SCFR | | Sequelae CFR rates | | Case fatality calculated or can be extrapolated from the data | 0: No  1: Yes  8: Not applicable  9: Unknown | | |
| 15 | DEF | | Sequelae definition | | Sequelae was defined | 0: No  1: Yes  8: Not applicable  9: Unknown | | |
| 16 | DEFL | | Sequelae definition LERG | | Sequelae was defined according to LERG definition (Quality assessment, Table 3) | 0: No  1: Yes  8: Not applicable  9: Unknown | | |
| 17 | SBIAS | | Paired sample | | Information available on proportion of subjects for whom paired and single samples were evaluated during laboratory confirmation | 8888: Not applicable  9999: Unknown | | |
| 18 | SUNC | | Sequelae for unconfirmed cases | | Sequelae determined for unconfirmed cases of leptospirosis | 0: No  1: Yes  8: Not applicable  9: Unknown | | |
| 19 | AGE | | Age rate | | Age specific attack rates calculated or can be extrapolated | 0: No  1: Yes  8: Not applicable  9: Unknown | | |
| 20 | SEX | | Sex rate | | Sex specific attack rates calculated or can be extrapolated | 0: No  1: Yes  8: Not applicable  9: Unknown | | |
| 21 | GRAL | | Sequelae studies general quality assessment evaluation | | Describes the final assessment of quality criteria evaluation of each disease sequelae report. | 0: Low quality of evidence  1: Medium quality of evidence  2: High quality of evidence | | |

**D: Data extraction**

| LERG dictionary for data extraction step | | | | |
| --- | --- | --- | --- | --- |
| **No.** | **Variable name** | **Variable** | **Description** | **Coding** |
| GENERAL VARIABLES | | | | |
| 1 | REFNO | Reference number | Reference number in LERG database | Nos. 1 - 11,462 |
| 2 | PUB | Publication year | Year of publication | 1945-2008 |
| 3 | REF | Reference | Journal or book name, issue and page number.  Note: journals articles from database BA doesn’t have a page number | 9999: Not available |
| 4 | AUT | Author | Last name and initials of all authors  Note: ISI database only provides data for first author | 9999: Not available |
| 5 | TIT | Title | Full title | 9999: Not available |
| 6 | ABST | Abstract text | Full text of abstract  Note: ISI database doesn’t have abstracts | 8888: Not available |
| 7 | QA | Quality assessment | Result of the quality assessment process | 0: Low quality of evidence  1: Medium quality of evidence  2: High quality of evidence |
| 8 | REG | Region | WHO-defined global regions. If study was done in a territory, the WHO region of the respective country was entered. | Region 1: Africa  Region 2: Eastern Mediterrian  Region 3: Europe  Region 4: Americas  Region 5: Southeast Asia  Region 6: Western Pacific |
| 9 | COU | Country | Country name. If study was done in a territory, the respective country name was entered. | 8=Not applicable  9: Unknown |
| 10 | GEO | Geographical setting | Area within country that the study was performed | 8:Not applicable  9: Unknown |
| 11 | PTY | Population type | Study population type, whether urban, rural or mixed. | 1: Urban  2: Rural  3: Mixed  8: Not applicable  9: Unknown |
| SPECIFIC VARIABLES FOR DISEASE INCIDENCE | | | | |
| 12 | DURY | Study duration in years | Study duration in years | 8: Not applicable  9: Unknown |
| 13 | FYR | Initial Year | First year of study on human leptospirosis or *Leptospira* infection | 8888: Not applicable  9999: Unknown |
| 14 | LYR | Final Year | Last year of study on human leptospirosis or *Leptospira* infection | 8888: Not applicable  9999: Unknown |
| 15 | YEARE | Year evaluated | Applicable to reports that have information for more than one. When disaggregated rates are available the reported year must be identified. | 8:Not applicable  9: Unknown |
| 16 | STR | Stratified incidence information | Describe information on cases stratified by year of occurrence. | 8:Not applicable  9: Unknown |
| 17 | POPU | Population used | Estimate of the inhabiting population used by the author for the geographical region under study. | 8:Not applicable  9: Unknown |
| 18 | SCASES | No. of confirmed and suspected cases per year | Number of patients per year with symptomatic illness with our without laboratory confirmation | 8:Not applicable  9: Unknown |
| 25 | CESY | No. of confirmed and probable cases per year | Number of patients with symptomatic illness who had laboratory *confirmed or probable* leptospirosis by any definition estimated in one year period. | 8:Not applicable  9: Unknown |
| 27 | SINC | Incidence of confirmed and suspected cases | Incidence (cases per 100,000 population per year) of leptospirosis confirmed and suspected cases identified by any definition. | 8888: Not applicable  9999: Unknown |
| 28 | INC | Incidence of confirmed and probable cases | Incidence (cases per 100,000 population per year) of leptospirosis *confirmed or probable* cases identified by any definition. | 8888: Not applicable  9999: Unknown |
| 31 | ISME* | Median incidence for confirmed and probable cases | Median incidence (cases per 100,000 population per year) of leptospirosis *confirmed or probable* cases by any definition in >1 year period. For example, when the duration of the study was 5 years and the information was available per year, a median incidence rate was calculated for the whole period. | 8888: Not applicable  9999: Unknown |
| 33 | ISDTS | Incidence by standard tests by sex (males) | Incidence (male cases per 100.000 population per year) of *confirmed or probable* cases by any definition by sex | 8888: Not applicable  9999: Unknown |
| 34 | ISDTA | Incidence by standard tests by age | Incidence (cases per 100.000 population per year) of *confirmed or probable* cases by any definition by age | 8888: Not applicable  9999: Unknown |
| 38 | DEATHYS | Estimated No. of suspected and confirmed deaths in one year period | Number of deaths estimated per year from leptospirosis, whether suspected or confirmed, which were identified within a defined study population. | 8888: Not applicable  9999: Unknown |
| 39 | DEATHY | Estimated No. of confirmed and probable deaths in one year period | Number of deaths estimated per year from *confirmed or probable* leptospirosis cases by any definitions, which were identified within a defined study population. | 8888: Not applicable  9999: Unknown |
| 41 | SMORT* | Mortality rate for suspected and confirmed cases | Incidence of mortality (deaths per 100,000 pop per year) from leptospirosis, whether confirmed or suspected, for the following population bases: national, regional, city, community-based populations. | 8888: Not applicable  9999: Unknown |
| 42 | MORT* | Mortality rate for confirmed and probable cases | Incidence of mortality (deaths per 100,000 pop per year) from *confirmed or probable* leptospirosis cases by any definition, for the following population bases: national, regional, city, community-based populations. | 8888: Not applicable  9999: Unknown |
| 44 | SCFR | Case fatality for suspected and confirmed cases | Prevalence of deaths among patients with confirmed or suspected leptospirosis. | 888: Not applicable  999: Unknown |
| 45 | CFR | Case fatality for confirmed and probable cases | Prevalence of deaths among patients with *confirmed or probable* leptospirosis by any definition. | 888: Not applicable  999: Unknown |
| 47 | OUT | Outbreak | Indicates if the cases were diagnosed during an outbreak. | 0: No  1: Yes  8: Not applicable  9: Unknown |
| 48 | DIADEF | Diagnosis definition | Definition used by the author to confirm diagnosed cases of leptospirosis. | Typed definition  NA: Not available |
|  | Nagesexcas | Number of cases with age and sex distribution | The number of cases for which the authors provided information about both age and sex that could be use to calculate the relative frequency of cases in demographic groups | 0: Not available |
|  | Nagesexdeath | Number of cases with age and sex distribution | The number of deaths for which the authors provided information about both age and sex that could be use to calculate the relative frequency of deaths due to leptospirosis in demographic groups | 0: Not available |
|  | Agecases | Number of cases with age distribution | The number of cases for which the authors provided information about age that could be use to calculate the relative frequency of deaths due to leptospirosis in demographic groups | 0: Not available |
|  | Agedeaths | Number of deaths with age distribution | The number of deaths for which the authors provided information about age that could be use to calculate the relative frequency of deaths due to leptospirosis in demographic groups | 0: Not available |
|  | MC0-9 … MC70+ | Percentage of cases in males within age groups | The percentage of cases in males within the following age groups: 0-9, 10-19, 20-29, 30-39, 40-49, 50-59, 60-69, 70+ | 9: Not applicable |
|  | FC0-9 … FC70+ | Percentage of cases in females within age groups | The percentage of cases in females within the following age groups: 0-9, 10-19, 20-29, 30-39, 40-49, 50-59, 60-69, 70+ | 9: Not applicable |
|  | MD0-9 … MD70+ | Percentage of deaths in males within age groups | The percentage of deaths in males within the following age groups: 0-9, 10-19, 20-29, 30-39, 40-49, 50-59, 60-69, 70+ | 9: Not applicable |
|  | FD0-9 … FD70+ | Percentage of deaths in females within age groups | The percentage of deaths in females within the following age groups: 0-9, 10-19, 20-29, 30-39, 40-49, 50-59, 60-69, 70+ | 9: Not applicable |
|  | C0-9 … C70+ | Percentage of cases in both genders within age groups | The percentage of cases in either gender within the following age groups: 0-9, 10-19, 20-29, 30-39, 40-49, 50-59, 60-69, 70+ | 9: Not applicable |
|  | D0-9 … D70+ | Percentage of deaths in both genders within age groups | The percentage of deaths in either gender within the following age groups: 0-9, 10-19, 20-29, 30-39, 40-49, 50-59, 60-69, 70+ | 9: Not applicable |
| SPECIFIC VARIABLES FOR DISEASE SEQUEALE | | | | |
| 49 | SCASES | No. of confirmed and suspected cases | Number of patients with symptomatic illness who had or not laboratory-*confirmed* leptospirosis by any definition. | 8:Not applicable  9: Unknown |
| 50 | NCASES | No. of confirmed cases | Number of patients with symptomatic illness who had laboratory-*confirmed* leptospirosis by any definition. | 8:Not applicable  9: Unknown |
| 53 | NTOT | No. of LERG confirmed and probable cases | Number of patients with symptomatic illness who had either laboratory-*confirmed* or laboratory-*probable* leptospirosis by LERG definition. | 8:Not applicable  9: Unknown |
| 54 | NSUSP | No. of clinically suspected cases | Number of patients with symptomatic illness who had clinically suspected leptospirosis. | 8:Not applicable  9: Unknown |
| 55 | SCDEATH | No. of confirmed and suspected deaths | Number of deaths from leptospirosis, whether suspected or confirmed by any definition. If both suspected and confirmed cases are reported, the number of deaths among suspected cases will be entered. | 8888: Not applicable  9999: Unknown |
| 56 | SCCFR | Case fatality for confirmed and suspected cases | Prevalence of deaths among patients with leptospirosis, whether confirmed or suspected. | 888: Not applicable  999: Unknown |
| 57 | CDEATH | No. of confirmed deaths | Number of deaths who had laboratory-*confirmed* leptospirosis by any definition. | 8888: Not applicable  9999: Unknown |
| 58 | CCFR | Case fatality for confirmed cases | Prevalence of deaths among patients who had laboratory-*confirmed* leptospirosis by any definition. | 888: Not applicable  999: Unknown |
| 59 | SDEATH | No. of suspected deaths | Number of deaths who had clinically suspected leptospirosis | 8888: Not applicable  9999: Unknown |
| 60 | SCFR | Case fatality for suspected cases | Prevalence of deaths among patients who had clinically suspected leptospirosis. | 888: Not applicable  999: Unknown |
| 61 | LAFI | Leptospirosis AFI of confirmed cases | Number of cases who had laboratory-*confirmed* leptospirosis with acute febrile illness. | 8: Not applicable  9: Unknown |
| 62 | PLAFI | % of AFI between confirmed cases | Proportion of cases who had laboratory-*confirmed* leptospirosis with acute febrile illness. | 8: Not applicable  9: Unknown |
| 63 | SCRI | ARI of confirmed and suspected cases | Number of registered cases with leptospirosis, whether suspected or confirmed, with acute renal injury | 8: Not applicable  9: Unknown |
| 64 | PSCRI | % of ARI in confirmed and suspected cases | Proportion of registered cases with leptospirosis, whether suspected or confirmed, with acute renal injury | 8: Not applicable  9: Unknown |
| 65 | RI | ARI of confirmed cases | Number of registered cases, who had laboratory-*confirmed* leptospirosis, with acute renal injury | 8: Not applicable  9: Unknown |
| 66 | PRI | % of ARI in confirmed cases | Proportion of cases, who had laboratory-*confirmed* leptospirosis, with acute renal injury | 8: Not applicable  9: Unknown |
| 67 | SRI | ARI of suspected cases | Number of registered clinically suspected cases of leptospirosis with acute renal injury | 8: Not applicable  9: Unknown |
| 68 | PSRI | % of ARI in suspected cases | Proportion of registered cases who had clinically suspected leptospirosis with acute renal injury | 8: Not applicable  9: Unknown |
| 69 | SSD | ARI in confirmed and suspected deaths | Number of deaths of leptospirosis, whether suspected or confirmed, registered with acute renal injury | 8: Not applicable  9: Unknown |
| 70 | PSSD | % of ARI of confirmed and suspected leptospirosis deaths | Proportion of deaths of leptospirosis, whether suspected or confirmed, registered between acute renal injury cases | 8: Not applicable  9: Unknown |
| 71 | SCD | ARI of confirmed leptospirosis deaths | Number of deaths who had laboratory-*confirmed* leptospirosis registered with acute renal injury | 8: Not applicable  9: Unknown |
| 72 | PSCD | % of ARI of confirmed leptospirosis deaths | Proportion of deaths who had laboratory-*confirmed* leptospirosis registered with acute renal injury | 8: Not applicable  9: Unknown |
| 73 | SD | ARI of suspected leptospirosis deaths | Number of deaths who had clinically suspected leptospirosis registered with acute renal injury | 8: Not applicable  9: Unknown |
| 74 | PSD | % of ARI of suspected leptospirosis deaths | Proportion of deaths who had clinically suspected leptospirosis registered with acute renal injury | 8: Not applicable  9: Unknown |
| 75 | RIH | ARI definition | Does the study include a definition for acute renal failure? If so, write the definition given in the “case definition” box in the data extraction sheet. | 8: Not applicable  9: Unknown |
| 76 | SCAPI | Acute lung injury of confirmed and suspected cases | Number of registered cases, whether suspected or confirmed, with acute lung injury | 8: Not applicable  9: Unknown |
| 77 | PSCAPI | % of acute lung injury of confirmed and suspected cases | Proportion of registered cases with leptospirosis, whether suspected or confirmed, with acute lung injury | 8: Not applicable  9: Unknown |
| 78 | CAPI | Acute lung injury of confirmed cases | Number of registered cases who had laboratory-*confirmed* leptospirosis with acute lung injury | 8: Not applicable  9: Unknown |
| 79 | PCAPI | % of acute lung injury of confirmed cases | Number of registered cases who had laboratory-*confirmed* leptospirosis with acute lung injury | 8: Not applicable  9: Unknown |
| 80 | SAPI | Acute lung injury of suspected cases | Number of registered cases who had clinically suspected leptospirosis with acute lung injury | 8: Not applicable  9: Unknown |
| 81 | SPAPI | % of acute lung injury of suspected cases | Number of registered cases who had clinically suspected leptospirosis with acute lung injury | 8: Not applicable  9: Unknown |
| 82 | SCAPID | Acute lung injury of confirmed and suspected leptospirosis deaths | Number of deaths of leptospirosis, whether suspected or confirmed, registered with acute lung injury | 8: Not applicable  9: Unknown |
| 83 | PSCAPID | % of acute lung injury of confirmed and suspected leptospirosis deaths | Proportion of deaths of leptospirosis, whether suspected or confirmed, registered with acute lung injury | 8: Not applicable  9: Unknown |
| 84 | CAPID | Acute lung injury deaths between confirmed cases | Number of deaths who had laboratory-*confirmed* leptospirosis registered with acute lung injury. | 8: Not applicable  9: Unknown |
| 85 | PCAPID | % of acute lung injury deaths between confirmed cases | Proportion of deaths who had laboratory-*confirmed* leptospirosis registered with acute lung injury | 8: Not applicable  9: Unknown |
| 86 | SAPID | Acute lung injury of suspected leptospirosis deaths | Number of deaths, who had clinically suspected leptospirosis, registered with acute lung injury | 8: Not applicable  9: Unknown |
| 87 | PSAPID | % of acute lung injury of suspected leptospirosis deaths | Proportion of deaths, who had clinically suspected leptospirosis, registered with acute lung injury | 8: Not applicable  9: Unknown |
| 88 | PDEF | Acute lung injury definition | Does the study include a definition for acute lung injury? If so, write the definition given in the “case definition” box in the data extraction sheet. | 8: Not applicable  9: Unknown |
| 89 | ARID | Renal failure duration | Time of duration of renal failure (days) in patients who had laboratory-*confirmed* leptospirosis | 8: Not applicable  9: Unknown |
| 90 | APID | Lung injury duration | Time of duration of lung injury (days) in patients who had laboratory-*confirmed* leptospirosis | 8: Not applicable  9: Unknown |
| 91 | OUT | Outbreak | Indicates if the cases were diagnosed during an outbreak. | 0: No  1: Yes  8: Not applicable  9: Unknown |
| 92 | AGEPOP | Age population | Indicates the age group in the study | 1: Children  2: Adult  8: Not applicable  9: Unknown |
| 93 | SET | Setting | Indicates if the study was performed in urban, rural or mixed setting. | 1: Urban  2: Rural  3: Mixed  8: Not applicable  9: Unknown |
| 94 | ACT | Active case ascertainment | Active case ascertainment whether community or hospital/provider-based | 0: No  1: Yes  8: Not applicable  9: Unknown |
| 95 | PAS | Passive case ascertainment | Passive case ascertainment was performed | 0: No  1: Yes  8: Not applicable  9: Unknown |
| 96 | ASC | Case ascertainment | Case ascertainment community or hospital based | 1: Community based  2: Hospital based  8: Not applicable  9: Unknown |
| 97 | OTHERS | Other information | Potential relevant information on included in the previous variables | 8: Not applicable  9: Unknown |
|  | NagesexAR | Number of cases of acute renal failure with age and gender information | Number of cases of acute renal failure with information about age and gender that could be used to calculate the relative frequency of this condition based on age and gender | 0: Not available |
|  | NagesexAL | Number of cases of acute lung injury with age and gender information | Number of cases of lung injury with information about age and gender that could be used to calculate the relative frequency of this condition based on age and gender | 0: Not available |
|  | NagesexARAL | Number of cases with both acute renal failure and acute lung injury with age and gender information | Number of deaths in patients with both acute renal failure and acute lung injury with information about age and gender that could be used to calculate the relative frequency of this condition based on age and gender | 0: Not available |
|  | NagesexARD | Number of deaths due to acute renal failure with age and gender information | Number of deaths due to acute renal failure with information about age and gender that could be used to calculate the relative fatality due to this condition based on age and gender | 0: Not available |
|  | NagesexALD | Number of deaths due to acute lung injury with age and gender information | Number of deaths due to lung injury with information about age and gender that could be used to calculate the relative fatality due to this condition based on age and gender | 0: Not available |
|  | NagesexARALD | Number of deaths in patients with both acute renal failure and acute lung injury with age and gender information | Number of deaths in patients with both acute renal failure and acute lung injury with information about age and gender that could be used to calculate the relative fatality due to this condition based on age and gender | 0: Not available |
|  | MAR0-9 … MAR70+ | Number of cases of acute renal injury in males within age groups | Number of cases of acute renal injury in males within the following age groups: 0-9, 10-19, 20-29, 30-39, 40-49, 50-59, 60-69, 70+ |  |
|  | FAR0-9 … FAR70+ | Number of cases of acute renal injury in females within age groups | Number of cases of acute renal injury in females within the following age groups: 0-9, 10-19, 20-29, 30-39, 40-49, 50-59, 60-69, 70+ |  |
|  | MAL0-9 … MAL70+ | Number of cases of acute lung injury in males within age groups | Number of cases of acute lung injury in males within the following age groups: 0-9, 10-19, 20-29, 30-39, 40-49, 50-59, 60-69, 70+ |  |
|  | FAL0-9 … FAL70+ | Number of cases of acute lung injury in females within age groups | Number of cases of acute lung injury in females within the following age groups: 0-9, 10-19, 20-29, 30-39, 40-49, 50-59, 60-69, 70+ |  |
|  | MARAL0-9 … MARAL70+ | Number of cases of both acute renal injury and acute lung injury in males within age groups | Number of cases of acute renal injury and acute lung injury in males within the following age groups: 0-9, 10-19, 20-29, 30-39, 40-49, 50-59, 60-69, 70+ |  |
|  | FARAL0-9 … FARAL70+ | Number of cases of both acute renal injury and acute lung injury in females within age groups | Number of cases of acute renal injury and acute lung injury infe males within the following age groups: 0-9, 10-19, 20-29, 30-39, 40-49, 50-59, 60-69, 70+ |  |
|  | MARD0-9 … MARD70+ | Number of deaths due to acute renal injury in males within age groups | Number of deaths due to acute renal injury in males within the following age groups: 0-9, 10-19, 20-29, 30-39, 40-49, 50-59, 60-69, 70+ |  |
|  | FARD0-9 … FARD70+ | Number of deaths due to acute renal injury in females within age groups | Number of deaths due to acute renal injury in females within the following age groups: 0-9, 10-19, 20-29, 30-39, 40-49, 50-59, 60-69, 70+ |  |
|  | MALD0-9 … MALD70+ | Number of deaths due to acute lung injury in males within age groups | Number of deaths due to acute lung injury in males within the following age groups: 0-9, 10-19, 20-29, 30-39, 40-49, 50-59, 60-69, 70+ |  |
|  | FALD0-9 … FALD70+ | Number of deaths due to acute lung injury in females within age groups | Number of deaths due to acute lung injury in females within the following age groups: 0-9, 10-19, 20-29, 30-39, 40-49, 50-59, 60-69, 70+ |  |
|  | MARALD0-9 … MARALD70+ | Number of deaths due to both acute renal injury and acute lung injury in males within age groups | Number of deaths due to acute renal injury and acute lung injury in males within the following age groups: 0-9, 10-19, 20-29, 30-39, 40-49, 50-59, 60-69, 70+ |  |
|  | FARALD0-9 … FARALD70+ | Number of deaths due to both acute renal injury and acute lung injury in females within age groups | Number of deaths due to acute renal injury and acute lung injury infe males within the following age groups: 0-9, 10-19, 20-29, 30-39, 40-49, 50-59, 60-69, 70+ |  |
|  |  |  |  |  |

* When the duration of the study was different from one year (for example, an outbreak), all the incidence rates and mortality calculations were extrapolated for a year.

**E: Indicator variables screened for regression model.**

| Adolescent Fertility Rate (births per 1,000 women ages 15-19) |
| --- |
| Adolescents Ages 13-15 Who Use Tobacco (Females) |
| Adolescents Ages 13-15 Who Use Tobacco (Males) |
| Agriculture as % of Gross Domestic Product |
| Birth Rate (annual number of births per 1,000 total population) |
| Births Attended by Medically Trained Personnel, Poorest Fifth (%) |
| Births Attended by Medically Trained Personnel, Middle Fifth (%) |
| Births Attended by Medically Trained Personnel, Richest Fifth (%) |
| Births Attended by Skilled Personnel (%) |
| Births per 1,000 Population |
| Births to Women Under Age 20 Attended by Skilled Personnel (%) |
| Change in Forest Area 1990-2000 (1,000 hectares) |
| Child Dependency Ratio |
| Child Mortality Rate (deaths per 1,000 children under age 5), 2005 |
| Children Fully Vaccinated, Poorest Fifth (%) |
| Children Fully Vaccinated, Middle Fifth (%) |
| Children Fully Vaccinated, Richest Fifth (%) |
| Children Stunted, Middle Fifth (%) |
| Children Stunted, Poorest Fifth (%) |
| Children Stunted, Richest Fifth (%) |
| CO2 Emissions per Capita, 1998 (metric tons) |
| CO2 Emissions per Capita, 2002 (metric tons) |
| Contraceptive Use Among Married Women 15-49, Condom (%) |
| Contraceptive Use Among Married Women 15-49, Most Used Method(s) |
| Contraceptive Use Among Married Women 15-49, Pill (%) |
| Contraceptive Use Among Married Women 15-49, Total Traditional Methods (%) |
| Contraceptive Use Among Married Women 15-49, Withdrawal (%) |
| Contraceptive Use Among Married Women Ages 15-19, Modern Method (%) |
| Contraceptive Use Among Married Women Ages 20-24, Modern Method (%) |
| Contraceptive Use Among Married Women, All Methods, Ages 15-49 (%) |
| Current Smokers, Female Students Ages 13-15 (%) |
| Current Smokers, Male Students Ages 13-15 (%) |
| Deaths Due to Non-Communicable Diseases |
| Deaths per 1,000 Population |
| Degrees latitude of geographic centroid |
| Density (population/sq. km.) |
| Economically Active Females, Ages 15-19 (%) 1990 |
| Economically Active Females, Ages 15-19 (%) 2005 |
| Economically Active Males, Ages 15-19 (%) 1990 |
| Economically Active Males, Ages 15-19 (%) 2005 |
| Economically Active, Female, Ages 15+, 1995-2002 (%) |
| Economically Active, Male, Ages 15+, 1995-2002 (%) |
| elderly support ratio (females) |
| elderly support ratio (males) |
| Ever-Married Females Ages 15-19 (%) |
| Ever-Married Males Ages 15-19 (%) |
| Forested area (percent) |
| Geographic centroid within tropics of cancer and capricorn |
| GNI PPP Per Capita, 2005 (US$) |
| Gross Domestic Product (million PPP$) |
| Gross Domestic Product Growth Rate |
| Gross Domestic Product per Capita (PPP$) |
| HIV/AIDS Among Adult Population, Ages 15-49, 2005/2006 (%) |
| HIV-Infected Adults Who Are Women Population, 2003-04 (%) |
| Infant Deaths per 1,000 Live Births |
| Island |
| Labor Force Participation Rate, Ages 15-24, Females |
| Labor Force Participation Rate, Ages 15-24, Males |
| Land area (square km) |
| Life Expectancy at Birth, Total |
| Life Expectancy at Birth, Female |
| Life Expectancy at Birth, Male |
| Lifetime Births per Woman (TFR) |
| Lifetime Chance of Dying from Maternal Causes, 1 in: |
| Literacy Rate, Ages 15-24, 2000-04, Female (%) |
| Literacy Rate, Ages 15-24, 2000-04, Male (%) |
| Literate Women as % of Literate Men, Ages 15-24, 2000-04 |
| Malnourished Women, Poorest Fifth (%) |
| Malnourished Women, Middle Fifth (%) |
| Malnourished Women, Richest Fifth (%) |
| Maternal Deaths per 100,000 Live Births, 2000 |
| Migration (net) per 1,000 Population |
| Mortality Rate, Under 5, Poorest Fifth |
| Mortality Rate, Under 5, Middle Fifth |
| Mortality Rate, Under 5, Richest Fifth |
| Mother's Attitude Toward Recent Births, Not Wanted (%) |
| Mother's Attitude Toward Recent Births, Wanted Later (%) |
| Natural Habitat Remaining (%) |
| Net Migration Rate (net migration per 1,000 population) |
| No. of Threatened and Endangered Species - Animals |
| No. of Threatened and Endangered Species - Plants |
| No. of Vehicles per 1,000 People, 2000 |
| Out-of-School Adolescents, Lower Secondary, Females |
| Out-of-School Adolescents, Lower Secondary, Males |
| Per Capita Public Expenditure on Health (US$) |
| Percent Enrolled in Tertiary Education, by Gender |
| Percent Unemployed, Ages 15-24, by Gender |
| Pop. Using Adequate Sanitation, 1999, Rural (%) |
| Pop. Using Adequate Sanitation, 1999, Total (%)  Pop. With Access to Improved Sanitation (%) |
| Population Age <15 (%) |
| Population Age 65+ (%) |
| Population Ages 15-24 with HIV/AIDS, 2001 (%) |
| Population Living Below US$1 per Day (%) |
| Population Mid-2007 |
| Pregnant Women With 3+ Antenatal Care Visits, Poorest Fifth (%) |
| Pregnant Women With 3+ Antenatal Care Visits, Middle Fifth (%) |
| Pregnant Women With 3+ Antenatal Care Visits, Richest Fifth (%) |
| Primary School Enrollment, 2000, % of School-Age Females Enrolled |
| Projected Pop. Change 2007-2050 (%) |
| Projected Population, 2025 |
| Projected Population, 2050 |
| Rate of Natural Increase (Percent) |
| School-Age Males Enrolled |
| Secondary School Enrollment, Female, 1990 (as % of school-age male enrollment) |
| Secondary School Enrollment, Female, 2000/2004 (as % of school-age enrollment) |
| Secondary School Enrollment, Female, 2000/2004 (as % of school-age male enrollment) |
| Secondary School Enrollment, Male, 2000/2004 (as % of school-age enrollment) |
| Small island (≤65,000 Km2 in area) |
| Small tropical island (small island with geographic centroid within tropics of cancer and capricorn) |
| Source of Contraceptive Supply: Other Private, Modern Methods (%) |
| Source of Contraceptive Supply: Private Medical, Modern Methods (%) |
| Source of Contraceptive Supply: Public, Modern Methods (%) |
| Total Fertility Rate, Middle Fifth |
| Total Fertility Rate, Poorest Fifth |
| Total Fertility Rate, Richest Fifth |
| Underweight Children Age <5 (%) |
| Unmarried Females Who Have Had Sex, Ages 15-19 (%) |
| Unmarried Males Who Have Had Sex, Ages 15-19 (%) |
| Unmet Need for Family Planning (%) |
| Urban Population (%) |
| Women Ages <15, 2005 (%) |
| Women Ages 15-19 Giving Birth in One Year (%) |
| Women Ages 15-19 Giving Birth in One Year, Poorest Fifth (%) |
| Women Ages 15-19 Giving Birth in One Year, Middle Fifth (%) |
| Women Ages 15-19 Giving Birth in One Year, Richest Fifth (%) |
| Women Ages 15-49, 2005 (%) |
| Women Ages 50+, 2005 (%) |
| Women All Ages, 2005 |
| Women as % of Nonfarm Wage Earners, 1990 |
| Women as % of Nonfarm Wage Earners, 2001-02 |
| Women as % of Parliament, 1995 |
| Women as % of Parliament, 2004 |
| Women Giving Birth by Age 18 (%) |
| Women Giving Birth in One Year, Ages 15-19 (%) |
| Women in Managerial or Technical Positions |
| Women Who Have Completed Fifth Grade, Middle Fifth (%) |
| Women Who Have Completed Fifth Grade, Poorest Fifth (%) |
| Women Who Have Completed Fifth Grade, Richest Fifth (%) |
| Women With Comprehensive Knowledge of HIV/AIDS, Ages 15-24 (%) |
| Women With Knowledge About Sexual Transmission of HIV/AIDS, Middle Fifth (%) |
| Women With Knowledge About Sexual Transmission of HIV/AIDS, Poorest Fifth (%) |
| Women With Knowledge About Sexual Transmission of HIV/AIDS, Richest Fifth (%) |
| Youth Ages 10-24 (% of total pop.) 2006 |
| Youth Ages 10-24, 2006 |
| Youth Ages 10-24, 2025 |
| Youth Ages 10-24, 2025 (% of total pop.) 2006 |

Data sources (If available for a given country, WHO or UN sources were preferred.)

WHO Global Health Observatory Database (<http://apps.who.int/ghodata>)

World Bank (<http://data.worldbank.org/indicator/>)

UN Population Division (<http://esa.un.org>)

Population reference bureau datasheet for 2011 ([www.prb.org](http://www.prb.org))

Programme for Water Supply and Sanitation ([www.wssinfo.org](http://www.wssinfo.org))
