# Supplementary material for: Global Morbidity and Mortality of Leptospirosis: A Systematic Review
Source: PLoS Negl Trop Dis. 2015 Sep 17;9(9):e0003898. doi: 10.1371/journal.pntd.0003898 (PMC4574773; doi:10.1371/journal.pntd.0003898)
Supplement: S1 Table — (DOCX) [file pntd.0003898.s004.docx]

**S1 Table: Quality assessment criteria**

| **Criteria** | **Surveillance studies** | **Cohort studies (including RCTs)** |
| --- | --- | --- |
| **Methods for identifying study population** | | |
| Study population | - The study was a population based investigation? - The population base was recently and reliably estimated (i.e. census)? | - Inclusion and exclusion criteria were defined for the cohort? - The cohort was representative of the population to be studied? |
| **Methods for measuring incidence** | | |
| Outcome | - Was laboratory confirmation performed for suspected cases? If so, standard diagnostic methods and criteria used according to LERG recommendations? - Was case ascertainment active or passive? - Was case ascertainment hospital-based or outpatient/community-based? - Did changes occur in the way that case ascertainment was performed or incidence was measured during the study period? | |
| Study period | - The study period was defined? - Surveillance performed for at least a one-year period in order to address seasonal variation in incidence measurements? | - The study period was defined? - The cohort was followed for at least a one-year period in order to address seasonal variation in rate measurements? |
| **Sources of bias** | | |
| Follow-up of suspected cases | - Is there information on proportion of suspected and confirmed cases for which single and paired sera were collected? | |
| Drop outs | - Not applicable | - Proportion of drop outs during follow up? |
| **Data analysis** | | |
| Analytic methods | - Were rates calculated? If not, can they be extrapolated or estimated from the data reported by the author? - Were age and sex specific attack rates determined or can they be calculated from the data? | |
