# Supplementary material for: Global Morbidity and Mortality of Leptospirosis: A Systematic Review
Source: PLoS Negl Trop Dis. 2015 Sep 17;9(9):e0003898. doi: 10.1371/journal.pntd.0003898 (PMC4574773; doi:10.1371/journal.pntd.0003898)
Supplement: S2 Table — (DOCX) [file pntd.0003898.s005.docx]

**S2 Table: Quality assessment checklist**

| **Criteria category** | **Quality** | | |
| --- | --- | --- | --- |
|  | **High:** The study fulfills all of the following criteria | **Medium:** The study does not fulfil criteria for high and low quality and in general has the following characteristics | **Low:** The study fulfills one or more of the following criteria |
| Study population | - Population based study; population base recently and reliably estimated | - Population based study; population base not recently or not reliably estimated | - Not a population based study |
| Measuring incidence | - Laboratory confirmation performed with standard methods and definitions, as defined by LERG - Active case ascertainment whether community or hospital/provider-based - Study period ≥1 year | - Laboratory confirmation performed but standard methods and definitions were not used - Passive hospital/provider-based case ascertainment - Study period <1 year | - Laboratory confirmation not performed. - Case ascertainment not performed as a systematic or continuous process during the study period. |
| Analysis | - Rates calculated or can be extrapolated from the data | - Rates calculated or can be extrapolated from the data | - Rates cannot be calculated or extrapolated from the data |
