# Supplementary material for: Global Morbidity and Mortality of Leptospirosis: A Systematic Review
Source: PLoS Negl Trop Dis. 2015 Sep 17;9(9):e0003898. doi: 10.1371/journal.pntd.0003898 (PMC4574773; doi:10.1371/journal.pntd.0003898)
Supplement: S3 Table — (DOCX) [file pntd.0003898.s006.docx]

S3 Table: Characteristics and findings of high and medium quality leptospirosis morbidity (N=80) and mortality (N=35) studies, according to GBD region

|  |  |  |  |  |  |  |  |  |  | **Confirmed cases or deaths per 100 000 population** | |  |
| --- | --- | --- | --- | --- | --- | --- | --- | --- | --- | --- | --- | --- |
| **Ref #** | **GBD region/ Site Country** | **Publication status** | **Study years** | **Duration (years)** | **Population studied** | **Setting** | **Quality** | **Surveillance** | **Case ascertainment** | **Morbidity** | **Mortality** | **Case fatality (%)** |
| High Income Asia Pacific (0 studies) | | |  |  |  |  |  |  |  |  |  |  |
| Central Asia (0 studies) | |  |  |  |  |  |  |  |  |  |  |  |
| East Asia (1 study) | |  |  |  |  |  |  |  |  |  |  |  |
| [[1](#_ENREF_1)] | Beijing and Fuzhou China | Grey literature | 1991 – 1996 | 6·0 | 13 040 000 | Urban | M | Passive | Hospital | 2·4 | – | – |
| South Asia (1 study) | | |  |  |  |  |  |  |  |  |  |  |
| [[2](#_ENREF_2)] | Kathmandu Nepal | Published | 2001 | 0·5 | 1 500 000 | Urban | M | Active | Hospital | 4·8 | – | – |
| South-East Asia (10 studies) | | |  |  |  |  |  |  |  |  |  |  |
| [[3](#_ENREF_3)] | Mayotte | Grey literature | 2009 – 2010 | 1·0 | 245 479 | Mixed | H | Active | Hospital | 25·7 | – | – |
| [[4](#_ENREF_4)] | Kamphaeng Phet Thailand | Published | 1998 – 2003 | 6·0 | 750 000 | Mixed | M | Active | Hospital | 1·5 | – | – |
| [[5](#_ENREF_5)] | Mahasarakam Province Thailand | Published | 1996 | 0·1 | 940 000 | Mixed | M | Passive | Hospital | 15·2 | – | – |
| [[6](#_ENREF_6)] | Nakornratchasrima Thailand | Published | 1998 | 0·3 | 2 500 000 | Rural | M | Active | Hospital | 7·4 | 0·4 | 4·8 |
| [[7](#_ENREF_7)] | Reunion | Published | 1985 – 1987 | 3·0 | 532 288 | Mixed | M | Passive | Hospital | 15·8 | 0·9 | 5·6 |
| [[8](#_ENREF_8)] | Reunion | Grey literature | 2004 – 2009 | 6·0 | 781 962 | Mixed | H | Active | Hospital | 4·2 | 0·3 | 8·0 |
| [[9](#_ENREF_9)] | Seychelles | Published | 1985 – 1990 | 6·0 | 66 000 | Mixed | M | Active | Hospital | 62·9 | 6·6 | 8·8 |
| [[10](#_ENREF_10)] | Seychelles | Published | 1995 – 1996 | 1·0 | 74 331 | Mixed | H | Active | Hospital | 100·9 | 8·1 | 8·0 |
| [[11](#_ENREF_11)] | Takeo Province Cambodia | Published | 2003 | 0·3 | 854 727 | Rural | M | Active | Hospital | 5·1 | – | – |
| [[12](#_ENREF_12)] | Thailand | Published | 1994 – 2003 | 7·0 | 66 202 532 | Mixed | M | Passive | Hospital | 9·5 | 0·3 | 3·2 |
| Australasia (6 studies) | |  |  |  |  |  |  |  |  |  |  |  |
| [[13](#_ENREF_13)] | Queensland Australia | Published | 1985 – 1996 | 11·0 | 3 114 035 | Mixed | M | Passive | Hospital | 1·9 | – | – |
| [[14](#_ENREF_14)] | Australia | Grey literature | 2004 – 2010 | 7·0 | 22 504 600 | Mixed | H | Active | Hospital | 0·6 | – | – |
| [[15](#_ENREF_15)] | Queensland Australia | Published | 1998 – 1999 | 1·1 | 3 214 284 | Mixed | M | Passive | Hospital | 3·2 | – | – |
| [[16](#_ENREF_16)] | Australia | Published | 1999 – 2003 | 5·0 | 19 881 500 | Mixed | M | Passive | Hospital | 1·1 | – | – |
| [[17](#_ENREF_17)] | Queensland Australia | Published | 1998 – 2004 | 7·0 | 3 941 966 | Mixed | M | Active | Hospital | 3·2 | 0·0 | 0·0 |
| [[18](#_ENREF_18)] | Raiatea Island and Marquises Island French Polynesia | Published | 2004 – 2005 | 1·0 | 19 500 | Mixed | M | Active | Hospital | 169·2 | – | – |
| Caribbean (14 studies) | |  |  |  |  |  |  |  |  |  |  |  |
| [[19](#_ENREF_19)] | Barbados | Published | 1970 – 1974 | 5·0 | 240 000 | Mixed | M | Passive | Community | 11·8 | 2·2 | 16·0 |
| [[20](#_ENREF_20)] | Barbados | Published | 1980 | 1·5 | 225 564 | Mixed | M | Passive | Hospital | 13·3 | 0·4 | 3·3 |
| [[21](#_ENREF_21)] | Barbados | Published | 1979 – 1986 | 8·0 | 254 000 | Mixed | M | Passive | Hospital | 12·2 | – | – |
| [[22](#_ENREF_22)] | Barbados | Published | 1979 – 1991 | 13·0 | 246 678 | Mixed | M | Active | Hospital | 12·4 | 1·7 | 13·8 |
| [[23](#_ENREF_23)] | Ciego de Avila Cuba | Published | 1980 – 1992 | 13·0 | 358 059 | Mixed | M | Passive | Hospital | 23·9 | 0·2 | 0·7 |
| [[24](#_ENREF_24)] | Cuba | Published | 1991 | 1·0 | 10 682 353 | Mixed | M | Passive | Hospital | 8·5 | – | – |
| [[25](#_ENREF_25)] | Ciego de Avila Cuba | Published | 1984 – 1988 | 5·0 | 335 260 | Mixed | M | Passive | Hospital | 17·3 | – | – |
| [[26](#_ENREF_26)] | Pinar del Rio Cuba | Published | 1996 – 1998 | 2·0 | 40 155 | Rural | M | Passive | Hospital | 39·8 | – | – |
| [[27](#_ENREF_27)] | French West Indies | Published | 1989 – 1993 | 5·0 | 450 000 | Mixed | M | Passive | Hospital | 3·0 | 0·5 | 17·6 |
| [[28](#_ENREF_28)] | Guadeloupe Archipelago and Basse Terre French West Indies | Published | 2003 – 2004 | 2·0 | 268 000 | Urban | M | Active | Hospital | 30·8 | – | – |
| [[29](#_ENREF_29)] | Martinique | Published | 1973 – 1975 | 3·0 | 350 000 | Mixed | M | Active | Hospital | 6·3 | – | – |
| [[30](#_ENREF_30)] | Martinique | Published | 1987 – 1992 | 6·0 | 502 646 | Mixed | M | Passive | Hospital | 6·3 | – | – |
| [[31](#_ENREF_31)] | Puerto Rico | Published | 1996 – 1997 | 2·0 | 3 800 000 | Mixed | M | Passive | Hospital | 0·6 | 0·1 | 21·7 |
| [[32](#_ENREF_32)] | Trinidad And Tobago | Published | 1977 – 1982 | 6·6 | 923 545 | Mixed | H | Active | Hospital | 2·6 | 0·2 | 8·2 |
| Central Europe (3 studies) | | |  |  |  |  |  |  |  |  |  |  |
| [[33](#_ENREF_33)] | Bulgaria | Published | 1989 – 2001 | 13·0 | 8 333 333 | Mixed | M | Passive | Hospital | 0·4 | <0·1 | 6·6 |
| [[34](#_ENREF_34)] | Bulgaria | Published | 2005 | 1·0 | 7 818 182 | Mixed | M | Passive | Hospital | 0·6 | 0·1 | 11·6 |
| [[35](#_ENREF_35)] | Slavonski Brod Croatia | Published | 1995 – 2005 | 11·0 | 125 000 | Mixed | M | Passive | Hospital | 3·9 | 0·4 | 8·1 |
| Eastern Europe (5 studies) | | |  |  |  |  |  |  |  |  |  |  |
| [[36](#_ENREF_36)] | Russia | Published | 1971 | 1·0 | 131 154 511 | Mixed | M | Passive | Hospital | 1·8 | – | – |
| [[37](#_ENREF_37)] | Moscow and Surrounding Areas Russia | Published | 1981 – 1993 | 13·0 | 15 150 784 | Mixed | M | Passive | Hospital | 0·4 | – | – |
| [[38](#_ENREF_38)] | Russia | Published | 1995 – 1999 | 5·0 | 147 225 000 | Mixed | M | Passive | Hospital | 2·4 | – | – |
| [[39](#_ENREF_39)] | Russia | Published | 2004 | 0·5 | 143 849 574 | Mixed | M | Passive | Hospital | 0·1 | – | – |
| [[39](#_ENREF_39)] | Krasnodar Russia | Published | 2004 | 0·5 | 5 125 221 | Mixed | M | Passive | Hospital | 1·0 | – | – |
| Western Europe (15 studies) | | |  |  |  |  |  |  |  |  |  |  |
| [[40](#_ENREF_40)] | France | Published | 1974 – 1983 | 10·0 | 54 272 727 | Mixed | M | Passive | Hospital | 0·2 | – | – |
| [[41](#_ENREF_41)] | France | Published | 1986 – 1992 | 7·0 | 56 436 000 | Mixed | M | Passive | Hospital | 0·5 | – | – |
| [[42](#_ENREF_42)] | France | Published | 1970 – 2003 | 34·0 | 55 630 000 | Mixed | M | Passive | Hospital | 0·2 | – | – |
| [[43](#_ENREF_43)] | France | Published | 1999 – 2000 | 0·7 | 45 740 425 | Mixed | M | Active | Hospital | 0·2 | – | – |
| [[44](#_ENREF_44)] | France | Published | 1996 – 2005 | 10·0 | 58 518 395 | Mixed | M | Passive | Hospital | 0·5 | – | – |
| [[45](#_ENREF_45)] | Aquitaine France | Published | 2004 – 2006 | 2·6 | 4 615 384 | Urban | M | Passive | Hospital | 0·5 | – | – |
| [[46](#_ENREF_46)] | Ireland | Published | 1985 – 1996 | 12·0 | 3 527 719 | Mixed | M | Passive | Hospital | 0·3 | <0·1 | 14·3 |
| [[47](#_ENREF_47)] | Ireland | Published | 1990 – 1996 | 7·0 | 166 209 | Mixed | M | Passive | Hospital | 10·4 | – | – |
| [[48](#_ENREF_48)] | Israel | Published | 1970 – 1979 | 10·0 | 4 482 143 | Mixed | M | Passive | Hospital | 0·7 | – | – |
| [[49](#_ENREF_49)] | Lombardy Italy | Published | 1974 – 1987 | 14·0 | 8 671 022 | Mixed | M | Passive | Hospital | 0·8 | 0·1 | 11·9 |
| [[50](#_ENREF_50)] | Netherlands | Published | 1991 – 1995 | 5·0 | 15 239 000 | Mixed | M | Passive | Hospital | 0·2 | 0·0 | 0·0 |
| [[51](#_ENREF_51)] | Netherlands | Published | 2003 – 2007 | 2·0 | 15 863 950 | Mixed | M | Passive | Hospital | 0·2 | – | – |
| [[52](#_ENREF_52)] | Netherlands | Grey literature | 1970 – 2008 | 39·0 | 14 849 000 | Mixed | M | Passive | Hospital | 0·2 | – | – |
| [[53](#_ENREF_53)] | Portugal | Published | 1991 – 1997 | 7·0 | 4 696 727 | Mixed | M | Passive | Hospital | 1·8 | 0·1 | 10·7 |
| [[54](#_ENREF_54)] | Portugal | Published | 1986 – 2003 | 18·0 | 5 842 845 | Mixed | M | Passive | Hospital | 0·9 | 0·1 | 21·7 |
| Andean Latin America (3 studies) | | |  |  |  |  |  |  |  |  |  |  |
| [[55](#_ENREF_55)] | Ecuador | Published | 1984 – 1996 | 13·0 | 1 000 000 | Mixed | M | Passive | Hospital | 1·2 | – | – |
| [[56](#_ENREF_56)] | Rural Iquitos Peru | Published | 2003 – 2004 | 0·8 | 474 000 | Rural | M | Active | Hospital | 33·2 | 0·0 | 0·0 |
| [[56](#_ENREF_56)] | Urban Iquitos Peru | Published | 2003 – 2004 | 0·8 | 400000 | Urban | M | Active | Hospital | 56·3 | 0·7 | 1·6 |
| Central Latin America (2 studies) | | |  |  |  |  |  |  |  |  |  |  |
| [[57](#_ENREF_57)] | Departamento del Atlantico Colombia | Published | 1999 – 2004 | 5·3 | 2 081 038 | Mixed | M | Passive | Hospital | 0·9 | 0·0 | 0·0 |
| [[58](#_ENREF_58)] | Yucatán Mexico | Published | 1998 – 2000 | 3·0 | 16 000 000 | Mixed | M | Active | Community | 0·1 | – | – |
| Southern Latin America (0 studies) | | |  |  |  |  |  |  |  |  |  |  |
| Tropical Latin America (10 studies) | | |  |  |  |  |  |  |  |  |  |  |
| [[59](#_ENREF_59)] | Salvador Brazil | Published | 1996 | 0·8 | 2 091 129 | Urban | M | Active | Hospital | 12·5 | 0·6 | 5·2 |
| [[60](#_ENREF_60)] | Salvador Brazil | Grey literature | 1996 – 2010 | 5·0 | 2 444 508 | Urban | H | Active | Hospital | 11·8 | 0·9 | 7·3 |
| [[61](#_ENREF_61)] | Rio De Janeiro Brazil | Published | 1985 – 1998 | 14·0 | 870 502 | Urban | M | Passive | Hospital | 29·6 | – | – |
| [[62](#_ENREF_62)] | Salvador Brazil | Published | 2000 | 0·8 | 2 211 539 | Urban | M | Active | Hospital | 5·4 | 0·1 | 2·0 |
| [[63](#_ENREF_63)] | Belém do Pará Brazil | Published | 1996 – 1999 | 4·0 | 1 175 181 | Urban | M | Passive | Hospital | 23·8 | – | – |
| [[64](#_ENREF_64)] | Rio Grande Do Sul Brazil | Published | 2001 | 1·0 | 10 187 798 | Mixed | M | Passive | Hospital | 12·5 | – | – |
| [[65](#_ENREF_65)] | São Paulo Brazil | Published | 1969 – 1997 | 28·0 | 36 966 527 | Urban | M | Passive | Hospital | 0·9 | – | – |
| [[66](#_ENREF_66)] | Santa Maria (Rio Grande Do Sul) Brazil | Published | 2001 – 2002 | 1·0 | 526 000 | Mixed | M | Passive | Hospital | 306·3 | 1·1 | 0·4 |
| [[67](#_ENREF_67)] | Rio De Janeiro Brazil | Published | 1997 – 2002 | 6·0 | 5 800 000 | Urban | M | Passive | Hospital | 1·4 | – | – |
| [[68](#_ENREF_68)] | Salvador Brazil | Published | 2001 | 0·5 | 2 400 000 | Urban | M | Active | Hospital | 7·4 | – | – |
| North Africa / Middle East (0 studies) | | |  |  |  |  |  |  |  |  |  |  |
| High Income North America (0 studies) | | |  |  |  |  |  |  |  |  |  |  |
| Oceania (8 studies) | |  |  |  |  |  |  |  |  |  |  |  |
| [[69](#_ENREF_69)] | New Caledonia | Published | 1973 – 1980 | 8·0 | 74 000 | Mixed | M | Passive | Hospital | 6·3 | – | – |
| [[70](#_ENREF_70)] | New Caledonia | Published | 1985 – 1986 | 2·0 | 145 368 | Mixed | M | Active | Hospital | 66·4 | 3·1 | 4·7 |
| [[71](#_ENREF_71)] | Nera and Coulee Basins New Caledonia | Published | 1985 – 1986 | 2·0 | 18 024 | Rural | M | Passive | Hospital | 231·4 | 0·0 | 0·0 |
| [[72](#_ENREF_72)] | New Caledonia | Published | 1989 | 1·0 | 165 000 | Mixed | M | Passive | Hospital | 87·3 | – | – |
| [[73](#_ENREF_73)] | Bourail New Caledonia | Published | 1989 – 1990 | 2·0 | 4 000 | Rural | M | Passive | Hospital | 975·0 | – | – |
| [[74](#_ENREF_74)] | New Caledonia | Grey literature | 2008-2009 | 2·0 | 244 410 | Mixed | H | Active | Hospital | 251·2 | 2·0 | 0·8 |
| [[75](#_ENREF_75)] | Kauai and Hawaii (Big Island) Hawaii United States | Published | 1988 – 1989 | 1·0 | 120 600 | Mixed | M | Active | Hospital | 32·5 | 1·7 | 6·1 |
| [[76](#_ENREF_76)] | Wallis and Futuna | Grey literature | 2004-2009 | 6·0 | 14 944 | Rural | H | Active | Hospital | 344·6 | 1·7 | 0·5 |
| Central Sub-Saharan Africa (0 studies) | | | | | |  |  |  |  |  |  |  |
| East Sub-Saharan Africa (1 study) | | | | |  |  |  |  |  |  |  |  |
| [[77](#_ENREF_77)] | Wonji Ethiopia | Published | 2003 | 0·3 | 52 000 | Urban | M | Active | Hospital | 160·3 | – | – |
| Southern Sub-Saharan Africa (0 studies) | | | | |  |  |  |  |  |  |  |  |
| West Sub-Saharan Africa (1 study) | | |  |  |  |  |  |  |  |  |  |  |
| [[78](#_ENREF_78)] | Cameroon | Published | 1975 – 1976 | 1·3 | 70 000 | Mixed | M | Passive | Hospital | 69·2 | 4·3 | 6·4 |

–, no data

**References and bibliographic details of studies used to estimate global leptospirosis cases and deaths.**

1. World Health Organization. Leptospirosis worldwide, 1999. Weekly Epidemiological Record. 1999; 74: 237-242. PMID: 10437435

2. Murdoch DR, Woods CW, Zimmerman MD, Dull PM, Belbase RH, et al. The etiology of febrile illness in adults presenting to Patan hospital in Kathmandu, Nepal. Am J Trop Med Hyg. 2004; 70: 670-675. PMID: 15211012

3. Lernout T, Picardeau M. Cire Océan Indien, Institut de Veille Sanitaire (InVS), Mayotte, France. Grey literature provided by LERG members. 2010:

4. Myint KSA, Gibbons RV, Murray CK, Rungsimanphaiboon K, Supornpun W, et al. Leptospirosis in Kamphaeng Phet, Thailand. Am J Trop Med Hyg. 2007; 76: 135. PMID: 17255242

5. Sejvar JJ, Tangkanakul W, Ratanasang P, Dowell SF, Sangjun N, et al. An outbreak of leptospirosis, Thailand–the importance of the laboratory. Southeast Asian J Trop Med Public Health. 2005; 36: 289-295. PMID: 15916032

6. Tangkanakul W, Tharmaphornpilas P, Plikaytis BD, Bragg SL, Poonsuksombat D, et al. Risk factors associated with leptospirosis in northeastern Thailand, 1998. Am J Trop Med Hyg. 2000; 63: 204-208. PMID: 11388516

7. Law-Koune J, D.G., Michault A. Human leptospirosis in Reunion Island: epidemiological study during 3 years (1985-1987). Bull Soc Pathol Exot Filiales. 1989; 82: 185-191

8. Magnin P. Service de Pneumologie et Maladies Infectieuses, GHSR - St. Pierre, Réunion, France. Grey literature by LERG members. 2010:

9. Pinn T. Leptospirosis in the Seychelles. The Medical Journal of Australia. 1992; 156: 163. PMID: 1545718

10. Yersin C, Bovet P, Mérien F, Wong T, Panowsky J, et al. Human leptospirosis in the Seychelles (Indian Ocean): a population-based study. Am J Trop Med Hyg. 1998; 59: 933-940. PMID: 9886203

11. Seng H, Sok T, Tangkanakul W, Petkanchanapong W, Kositanont U, et al. Leptospirosis in Takeo Province, Kingdom of Cambodia, 2003. J Med Assoc Thailand. 2007; 90: 546. PMID: 17427534

12. Tangkanakul W, Smits HL, Jatanasen S, Ashford DA. Leptospirosis: an emerging health problem in Thailand. The Southeast Asian journal of tropical medicine and public health. 2005; 36: 281-288. PMID: 15916031

13. Norris MA, Smythe LD, Symonds ML, Dohnt MF, Scott J. Review of leptospirosis notifications in Queensland 1985 to 1996. Commun Dis Intell. 1997; 21: 17. PMID: 9079586

14. Smythe LD. WHO Collaborating Centre for Reference and Research on Leptospirosis, Brisbane, Australia. Grey literature provided by LERG members. 2010.

15. Smythe LD, Dohnt MF, Symonds ML, Barnett L, Moore M, et al. Review of leptospirosis notifications in Queensland and Australia: January 1998-June 1999. Commun Dis Intell. 2000; 24: 153-157. PMID: 10943028

16. Miller M, Roche P, Yohannes K, Spencer J, Bartlett M, et al. Australia's notifiable diseases status, 2003 annual report of the National Notifiable Diseases Surveillance System. Commun Dis Intell. 2005; 29: 1. PMID: 15966675

17. Slack AT, Symonds ML, Dohnt MF, Smythe LD. The epidemiology of leptospirosis and the emergence of Leptospira borgpetersenii serovar Arborea in Queensland, Australia, 1998-2004. Epidemiol Infect. 2006; 134: 1217-1225.10.1017/S0950268806006352 PMID: 16690001

18. Coudert C, Beau F, Berlioz-Arthaud A, Melix G, Devaud F, et al. [Human leptospirosis in French Polynesia. Epidemiological, clinical and bacteriological features]. Med Trop (Mars). 2007; 67: 137-144. PMID: 17691431

19. Damude D, Jones C, White HSC, Myers D. The problem of human leptospirosis in Barbados. Transactions of the Royal Society of Tropical Medicine and Hygiene. 1979; 73: 169-177. PMID: 473305

20. Edwards CNN, Nicholson GDD, Everard CO, Edwards CN Hassell TA EC, Callender J, Nicholson G D. Thrombocytopenia in leptospirosis. Rev Inst Med Trop Sao Paulo. 1982; 31: 827. PMID: 7102918

21. Everard CO, Bennett S, Edwards CN, Nicholson GD, Hassell TA, et al. An investigation of some risk factors for severe leptospirosis on Barbados. J Trop Med Hyg. 1992; 95: 13. PMID: 1740814

22. Everard CO, Edwards CN, Everard J, Carrington DG. A twelve-year study of leptospirosis on Barbados. Eur J Epidemiol. 1995; 11: 311-320. PMID: 7493664

23. Suárez Hernández M, Bustelo Aguila J, Sosa Pérez O, Portilla AL, Pelaez Martínez R, et al. Análisis de la letalidad por leptospirosis en la provincia Ciego de Avila, Cuba 1980-1992. Rev Asoc Guatemalteca Parasitol Med Trop. 1993; 8: 4-8

24. Martinez Sanchez R, C.d.l.P.R., Lopez Acosta C. Algunas consideraciones sobre el comportamiento de la leptospirosis humana en Cuba. Rev Cuba Med Trop. 1993; 45: 32-42. PMID: 7800887

25. Suárez Hernández M, Santisteban MR, Cabrera CJ, del Risco A, Pelaéz MR. A clinico-epidemiological study of leptospirosis in adults in the province of Ciego de Avila. Rev Clin Esp. 1995; 195: 459. PMID: 7667520

26. Cañete Villafranca R, Martinez Sánchez R, Suárez Delgado O, López Piñera O. Comportamiento clinico-epidemiológico de la leptospirosis humana en el municipio Los Palacios, provincia Pinar del Rio, Cuba. Rev Cuba Med Trop. 2002; 54: 15-20. PMID: 15846934

27. Dupont H, Dupont-Perdrizet D, Perie JL, Zehner-Hansen S, Jarrige B, et al. Leptospirosis: prognostic factors associated with mortality. Clin Infect Dis. 1997; 25: 720-724. PMID: 9314467

28. Storck CH, Postic D, Lamaury I, Perez JM. Changes in epidemiology of leptospirosis in 2003--2004, a two El Niño Southern Oscillation period, Guadeloupe archipelago, French West Indies. Epidemiology and infection. 2008; 136: 1407-1415. doi: 10.1017/S0950268807000052 PMID: 18096102

29. Mailloux M, Schneider R, Gervaise G. [Human leptospirosis in Martinique]. B Soc Pathol Exot. 1976; 69: 144. PMID: 1037091

30. L'Homme V, Grolierbois L, Jouannelle J, Elisabeth L. Leptospirose en Martinique de 1987 à 1992 : bilan d'une étude épidémiologique, clinique et biologique. Med Mal Infect. 1996; 26: 94-98.

31. Bruce M, Sanders E, Leake J, Zaidel O, Bragg SL, et al. Leptospirosis among patients presenting with dengue-like illness in Puerto Rico. Acta Trop. 2005; 96: 36-46. PMID: 16083836

32. Everard CO, Fraser-Chanpong G, Everard J. The incidence of severe leptospirosis in Trinidad. Trop Geogr Med. 1987; 39: 126. PMID: 3629704

33. Christova I, Tasseva E, Manev H. Human leptospirosis in Bulgaria, 1989–2001: epidemiological, clinical, and serological features. J Infect Dis. 2003; 35: 869-872. PMID: 14723364

34. Tasseva E, Christova I, Gladnishka T. Epidemiological, clinical and serological features of human leptospirosis in Bulgaria in 2005. European Society of Clinical Microbiology and Infectious Diseases. 2007; pp. Abstract number 1733_1549.

35. Cvitkovic A. Human leptospirosis in Slavonski Brod, 1995-2005. Acta Med Croat. 2007; 61: 349-353. PMID: 18044467

36. Ivanova LM. [Current epidemiology of natural focus infections in the RSFSR]. Medicinskaâ parazitologiâ i parazitarnye bolezni. 1984: 17-21. PMID: 6145090

37. Cherkasskiĭ BL, Shumilov PK, Manenkova GM. [The epidemiologic-epizootiologic characteristics of leptospirosis in the Moscow region]. Medicinskaâ parazitologiâ i parazitarnye bolezni. 1996: 32-36. PMID: 8926932

38. Makeyev S, Maramovich A, Yaroshenko V, Kuznetsov A, Kondakov A, et al. Epidemiological aspects of leptospirosis in the eastern regions of the Russian Federation. Medicinskaâ parazitologiâ i parazitarnye bolezni. 2002: 15-20. PMID: 12557580

39. Russian News Agency. Leptospirosis - Russia: 2004, RFI. ProMEDmail Archive Number 200501190178. 2005.

40. Mailloux M, Raoult D, Chaudet H. Surveillance of icterohemorrhagic leptospirosis in France (1974-1983). Rev Epid San Pub. 1985; 33: 425. PMID: 3914013

41. Baranton G. Human Leptospirosis in France from 1986 to 1992. Med Mal Infect. 1993; 23: 499-503

42. Baranton G, Postic D. Trends in leptospirosis epidemiology in France. Sixty-six years of passive serological surveillance from 1920 to 2003. Int J Infect Dis. 2006; 10: 162-170. PMID: 16298537

43. Nardone A, Campese C, Capek I, Sanitaire IdV. Les facteurs de risques de leptospirose en France métropolitaine : Une étude cas-témoin, juillet 1999-février 2000 (Surveillance). 2002.

44. Janin C, Alsibaï S. Survey on leptospirosis incidence in the Ardennes district, France, 1996-2005. Bulletin Épidémiologique Hebdomadaire. 2008: 164-167

45. Castor C, Servas V. Étude descriptive et prospective de l’incidence de la leptospirose en Aquitaine, 2004-2006. Rev Epid San Pub. 2008; 56: 268

46. Hogan MC, Pate G, McConkey SJ, O'Flanagan D, Mongan C, et al. Leptospirosis in the Republic of Ireland: 1985 to 1996. Commun Dis Rep CDR Rev. 1997; 7: R185-189. PMID: 9394061

47. Pate G, FitzSimon N, Mellotte GJ. Leptospirosis in the South-Eastern Health Board region of the Republic of Ireland: 1990 to 1996. Commun Dis Public Health. 1999; 2: 217-218. PMID: 10491881

48. Shenberg E, Gerichter CB, Lindenbaum I. Leptospirosis in man, Israel, 1970-1979. Am J Epidemiol. 1982; 115: 352-358. PMID: 7064970

49. Gelosa L, Perone A. [Seroepidemiologic study of human leptospirosis in Lombardy]. B I Sieroter Milan. 1989; 68: 127-141. PMID: 2491297

50. Olszyna DP, Jaspars R, Speelman P, van Elzakker E, Korver H, et al. [Leptospirosis in the Netherlands, 1991-1995]. Ned Tijdschr Geneeskd. 1998; 142: 1270-1273. PMID: 9749300

51. Hartskeerl RA, Goris MGA. Meer leptospirose in 2007. Infectieziekten Bulletin. 2008.

52. Hartskeerl RA. National Leptospirosis Reference Centre at KIT Biomedical Research, Netherlands. Grey literature provided by LERG members. 2010:

53. Falcao J, Nogueira P, MatiasDias C, Pimenta Z. Leptospirosis in Portugal: epidemiology from 1991 to 1997. Euro Surveill. 1999; 4: 44. PMID: 12631907

54. Vieira ML, Gama-Simões MJ, Collares-Pereira M. Human leptospirosis in Portugal: a retrospective study of eighteen years. Int J Infect Dis. 2006; 10: 378-386. PMID: 16600656

55. Medina Dávalos M, Borja Cevallos G. Panorama epidemiologist of the leptospirosis in Ecuador. Rev CIEZT. 1998; 3: 17-26

56. Segura ER, Ganoza CA, Campos KJ, Ricaldi JN, Torres S, et al. Clinical spectrum of pulmonary involvement in leptospirosis in a region of endemicity, with quantification of leptospiral burden. Clinical infectious diseases. 2005; 40: 343-351. doi: 10.1086/427110 PMID: 15668855

57. Macías-Herrera JC, Vergara C, Romero-Vivas C, Falconar AKI. Behavior of leptospirosis in Atlantic Department (Colombia)January 1999 ûMarch 2004. Salud UNINORTE. 2005; 20: 18-29

58. Vado-Solis I, Cardenas-Marrufo MF, Jimenez-Delgadillo B, Alzina-López A, Laviada-Molina H, et al. Clinical-epidemiological study of leptospirosis in humans and reservoirs in Yucatan, Mexico. Rev Inst Med Trop Sao Paulo. 2002; 44: 335-340. PMID: 12532218

59. Ko AI, Galvão Reis M, Ribeiro Dourado CM, Johnson WD, Riley LW. Urban epidemic of severe leptospirosis in Brazil. Salvador Leptospirosis Study Group. Lancet. 1999; 354: 820-825. doi: 10.1016/S0140-6736(99)80012-9 PMID: 10485724

60. Ko AI. Centro de Pesquisas Gonçalo Moniz, Fundação Oswaldo Cruz, Ministério da Saúde, Salvador, Brazil. Grey literature provided by LERG members. 2010.

61. Romao AR. Enchentes, deslizamentos e leptospirose no contexto das chuvas intensas: o caso da regiao AP3. 1. Escola Nacional de Saúde Pública Rio de Janeiro, 2001: Escola Nacional de Saude Publica. 2001.

62. Sarkar U, Nascimento SF, Barbosa R, Martins R, Nuevo H, et al. Population-based case-control investigation of risk factors for leptospirosis during an urban epidemic. Am J Trop Med Hyg. 2002; 66: 605-610. PMID: 12201599

63. Duarte ARSM, Couto RCS, Castro EMR, Marin RA, Gomes LS. Ocupacao desordenada do espaco urbano e politica de saude: um estudo sobre Belem-Para. Saude, trabalho e meio ambiente: politicas publicas na Amazonia, Belem. Belem: Universidade Federal do Para. Nucleo de Estudos Amazonicos. 2002; pp. 63-82.

64. Barcellos C, Lammerhirt CB, de Almeida MAB, dos Santos E. [Spatial distribution of leptospirosis in Rio Grande do Sul, Brazil: recovering the ecology of ecological studies]. Cad Saude Publica. 2003; 19: 1283-1292. PMID: 14666210

65. Romero EC, Bernardo CCM, Yasuda PH. Human leptospirosis: a twenty-nine-year serological study in São Paulo, Brazil. Rev Inst Med Trop Sao Paulo. 2003; 45: 245-248. PMID: 14743663

66. Brum L, Kupek E. Record linkage and capture-recapture estimates for underreporting of human leptospirosis in a Brazilian health district. J Infect Dis. 2005; 9: 515-520. PMID: 16410948

67. Tassinari WdS, Pellegrini DDCP, Sa CBP, Reis RB, Ko AI, et al. Demographic census of Rio de Janeiro Brazil. Trop Med Intern Health. 2008; 13: 503-512

68. Maciel EAP, De Carvalho ALF, Nascimento SF, De Matos RB, Gouveia EL, et al. Household transmission of leptospira infection in urban slum communities. PLoS Negl Trop Dis. 2008; 2: e154. doi 10.1371/journal.pntd.0000154 PMID: 18357340

69. Guelain J, Le Gonidec G, Bouchard E, Peghini M. [Leptospiroses in New Caledonia]. Med Trop (Mars). 1983; 43: 137 PMID: 6865708

70. Brethes B, Puech PL, Fraisse A, Dubois P, Domenech J, et al. [Epidemiological study of leptospirosis in New Caledonia]. B Soc Pathol Exot. 1988; 81: 189-197. PMID: 3231845 PMID: 3416406

71. Brethes B, Puech PL, Fraisse A, Dubois P, Domenech J, et al. [Leptospirosis and environment. Study of 2 major foci in New Caledonia]. Rev Epid San Pub. 1988; 36: 436. PMID: 3231845

72. Perrocheau A, Perolat P. Epidemiology of leptospirosis in New Caledonia (South Pacific): a one-year survey. Eur J Epidemiol. 1997; 13: 161-167. PMID: 9084999

73. Bouree P, Benoist L, Perolat P. [Epidemiologic and clinical study of leptospirosis in Bourail (New Caledonia)]. B Soc Pathol Exot. 1999; 92: 51. PMID: 10214523

74. Goarant C. La direction des affaires sanitaires et sociales (DASS) de Nouvelle-Calédonie, New Caledonia, France. Grey literature provided by LERG members. 2010.

75. Sasaki DM, Pang L, Minette HP, Wakida CK, Fujimoto WJ, et al. Active surveillance and risk factors for leptospirosis in Hawaii. Am J Trop Med Hyg. 1993; 48: 35. PMID: 8427386

76. Meynard D, Picardeau M. Agence de Santé des îles Wallis & Futuna /Hhôpital de SIA, Wallis and Futuna, France. Grey literature provided by LERG members. 2010.

77. Yimer E, Koopman S, Messele T, Wolday D, Newayeselassie B, et al. Human leptospirosis in Ethiopia: a pilot study in Wonji. Ethiop J Health Dev. 2004; 18: 48-51

78. Le Bras J, Guyer B, Sulzer C, Mailloux M. [Anademic focus of leptospirosis at Fondem (U.R. of Cameroon)]. B Soc Pathol Exot. 1977; 70: 569-583. PMID: 615682
