# Supplementary material for: Global Morbidity and Mortality of Leptospirosis: A Systematic Review
Source: PLoS Negl Trop Dis. 2015 Sep 17;9(9):e0003898. doi: 10.1371/journal.pntd.0003898 (PMC4574773; doi:10.1371/journal.pntd.0003898)
Supplement: S4 Table — (DOCX) [file pntd.0003898.s007.docx]

S4 Table: Geographic distribution of high and medium quality studies, according to GBD region (A) and WHO sub-region (B)

| **A) GBD region** | **Morbidity studies (N=80)** | **Mortality studies (N=35)** |
| --- | --- | --- |
| High Income Asia Pacific | – | – |
| Central Asia | – | – |
| East Asia | 1 (1 country) | – |
| South Asia | 1 (1 country) | – |
| South-East Asia | 10 (5 countries) | 6 (3 countries) |
| Australasia | 6 (2 countries) | 1 (1 country) |
| Caribbean | 14 (6 countries) | 7 (5 countries) |
| Central Europe | 3 (2 countries) | 3 (2 countries) |
| Eastern Europe | 5 (1 country) | – |
| Western Europe | 15 (6 countries) | 5 (4 countries) |
| Andean Latin America | 3 (2 countries) | 2 (1 country) |
| Central Latin America | 2 (2 countries) | 1 (1 country) |
| Southern Latin America | – | – |
| Tropical Latin America | 10 (1 country) | 4 (1 country) |
| North Africa / Middle East | – | – |
| High Income North America | – | – |
| Oceania | 8 (3 countries) | 5 (3 countries) |
| Central Sub-Saharan Africa | – | – |
| East Sub-Saharan Africa | – | – |
| Southern Sub-Saharan Africa | – | – |
| West Sub-Saharan Africa | 1 (1 country) | 1 (1 country) |

| **B) WHO sub-region**^a^ | **Morbidity studies (N=80)** | **Mortality studies (N=35)** |
| --- | --- | --- |
| AFR-D | 3 (2 countries) | 3 (2 countries) |
| AFR-E | 1 (1 country) | – |
| AFR-Colonies and Territories^b^ | 3 (2 territories) | 2 (1 territory) |
| AMR-A | 4 (1 country) | 1 (1 country) |
| AMR-B | 17 (5 countries) | 9 (4 countries) |
| AMR-D | 3 (2 countries) | 2 (1 country) |
| AMR-Colonies and Territories^c^ | 6 (4 territories) | 3 (3 territories) |
| EMR-B | – | – |
| EMR-D | – | – |
| EUR-A | 16 (7 countries) | 6 (5 countries) |
| EUR-B | 2 (1 country) | 2 (1 country) |
| EUR-C | 5 (1 country) | – |
| SEAR-B | 4 (1 country) | 2 (1 country) |
| SEAR-D | 1 (1 country) | – |
| WPR-A | 5 (1 country) | 1 (1 country) |
| WPR-B | 2 (2 countries) | – |
| WPR-Colonies and Territories^d^ | 8 (3 countries) | 4 (2 countries) |

–, no studies. ^a^ WHO sub-region mortality strata: A, very low child, low adult; B, low child, low adult; C, low child, high adult; D, high child, high adult; E, high child, very high adult. ^b^ Includes non-independent colonies and territories in AFR region: Mayotte, Reunion. ^c^ Includes non-independent colonies, states, and territories in AMR region: American Virgin Islands, Anguilla, Bermuda, British Virgin Islands, Cayman Islands, French Guiana, Guadeloupe, Guam, Hawaii, Martinique, Monseratte, Netherlands Antilles, Puerto Rico, St Pierre and Miquelon, Turks and Caicos. ^d^ Includes non-independent colonies and territories in WPR region: French Polynesia, Hong Kong, Macao, New Caledonia, Taiwan, Wallis and Futuna.
