# Supplementary material for: Global Morbidity and Mortality of Leptospirosis: A Systematic Review
Source: PLoS Negl Trop Dis. 2015 Sep 17;9(9):e0003898. doi: 10.1371/journal.pntd.0003898 (PMC4574773; doi:10.1371/journal.pntd.0003898)
Supplement: S5 Table — (DOCX) [file pntd.0003898.s008.docx]

S5 Table: Studies that reported information on age and gender proportions of cases and deaths from leptospirosis. This table includes studies with information on cases or deaths according to gender and the following age-groups: 0-9, 10-19, 20-29, 30-39, 40-49, 50-59, 60-69, 70+ years.

| **Reference no.** | **Location** | **Cases** | **Deaths** |
| --- | --- | --- | --- |
| [[1](#_ENREF_1)] | Mayotte | 63 | 1 |
| [[2](#_ENREF_2)]* | Réunion | 199 | 16 |
| [[3](#_ENREF_3)] | Seychelles | 75 | – |
| [[4](#_ENREF_4)] | Australia | 825 | – |
| [[5](#_ENREF_5)] | Barbados | 215 | – |
| [[6](#_ENREF_6)] | Ireland | 116 | – |
| [[7](#_ENREF_7)]* | Netherlands | 1037 | 34 |
| [[8](#_ENREF_8)]* | Salvador, Brazil | 1438 | 105 |
| [[9](#_ENREF_9)] | New Caledonia | 1811 | 7 |
| [[10](#_ENREF_10)] | Wallis and Futuna | 232 | 1 |

– , No data reported; *indicates the three studies that met criteria for calculating age and gender specific risk for death from leptospirosis.

**References**

1. Lernout T, Picardeau M. Cire Océan Indien, Institut de Veille Sanitaire (InVS), Mayotte, France. Grey literature provided by LERG members.2010

2. Magnin P. Service de Pneumologie et Maladies Infectieuses, GHSR - St. Pierre, Réunion, France. Grey literature provided by LERG members. 2010.

3. Yersin C, Bovet P, Mérien F, Wong T, Panowsky J, et al. Human leptospirosis in the Seychelles (Indian Ocean): a population-based study. Am J Trop Med Hyg. 1998; 59: 933-940.

4. Smythe LD. WHO Collaborating Centre for Reference and Research on Leptospirosis, Brisbane, Australia. Grey literature provided by LERG members. 2010.

5. Damude D, Jones C, White HSC, Myers D The problem of human leptospirosis in Barbados. Transactions of the Royal Society of Tropical Medicine and Hygiene. 1979; 73: 169-177.

6. Hogan MC, Pate G, McConkey SJ, O'Flanagan D, Mongan C, et al. Leptospirosis in the Republic of Ireland: 1985 to 1996. Commun Dis Rep CDR Rev 1997; 7: R185-189.

7. Hartskeerl RA. National Leptospirosis Reference Centre at KIT Biomedical Research, Netherlands. Grey literature provided by LERG members.2010.

8. Ko AI. Centro de Pesquisas Gonçalo Moniz, Fundação Oswaldo Cruz, Ministério da Saúde, Salvador, Brazil. Grey literature provided by LERG members.2010.

9. Goarant C. La direction des affaires sanitaires et sociales (DASS) de Nouvelle-Calédonie, New Caledonia, France Grey literature provided by LERG members. 2010.

10. Meynard D, Picardeau M. Agence de Santé des îles Wallis & Futuna /Hhôpital de SIA, Wallis and Futuna, France. Grey literature provided by LERG members. 2010.
