# Supplementary material for: Global Morbidity and Mortality of Leptospirosis: A Systematic Review
Source: PLoS Negl Trop Dis. 2015 Sep 17;9(9):e0003898. doi: 10.1371/journal.pntd.0003898 (PMC4574773; doi:10.1371/journal.pntd.0003898)
Supplement: S6 Table — (DOCX) [file pntd.0003898.s009.docx]

S6 Table: Relative risk of leptospirosis cases (N=10 studies) and deaths (N=3 studies) according to age and gender group.

| **Demographic group** | **RR Case (SD)** | **RR Death (SD)** |
| --- | --- | --- |
| Males |  |  |
| 0-9 years | 0·254 (0·201) | 0·465 (0·158) |
| 10-19 years | 1·392 (0·248) | 0·396 (0·391) |
| 20-29 years | 2·380 (0·877) | 1·114 (0·557) |
| 30-39 years | 2·183 (0·777) | 1·173 (0·422) |
| 40-49 years | 1·930 (0·573) | 3·406 (0·440) |
| 50-59 years | 1·875 (0·473) | 3·682 (0·556) |
| 60-69 years | 1·538 (0·439) | 3·138 (0·374) |
| 70+ years | 1·011 (0·568) | 2·371 (1·420) |
| Females |  |  |
| 0-9 years | 0·031 (0·043) | 0·149 (0·103) |
| 10-19 years | 0·246 (0·096) | 0·251 (0·145) |
| 20-29 years | 0·499 (0·380) | 0·136 (0·094) |
| 30-39 years | 0·609 (0·542) | 0·077 (0·053) |
| 40-49 years | 0·538 (0·369) | 0·629 (0·434) |
| 50-59 years | 0·603 (0·500) | 0·604 (0·350) |
| 60-69 years | 0·657 (0·552) | 0·596 (0·503) |
| 70+ years | 0·341 (0·361) | 0·798 (0·353) |
