# Supplementary material for: Global Morbidity and Mortality of Leptospirosis: A Systematic Review
Source: PLoS Negl Trop Dis. 2015 Sep 17;9(9):e0003898. doi: 10.1371/journal.pntd.0003898 (PMC4574773; doi:10.1371/journal.pntd.0003898)
Supplement: S7 Table — (DOCX) [file pntd.0003898.s010.docx]

S7 Table: Studies which reported information on both clinically-suspected and laboratory-confirmed leptospirosis cases and deaths.

| **Ref No.** | **Country** | **Suspected annual morbidity** | **Confirmed annual morbidity** | **Suspected annual mortality** | **Confirmed annual mortality** | **Suspected case-fatality ratio** | **Confirmed case-fatality ratio** |
| --- | --- | --- | --- | --- | --- | --- | --- |
| [[1](#_ENREF_1)] | Nepal | 116·8 | 4·8 | – | – | – | – |
| [[2](#_ENREF_2)] | Thailand | 2·4 | 1·5 | – | – | – | – |
| [[3](#_ENREF_3)] | Thailand | 9·5 | 7·4 | – | 0·4 | – | 4·8 |
| [[4](#_ENREF_4)] | Seychelles | 173·5 | 100·9 | 16·1 | 8·1 | 9·3 | 8·0 |
| [[5](#_ENREF_5)] | Cambodia | 56·2 | 5·1 | – | – | – | – |
| [[6](#_ENREF_6)] | Barbados | 25·4 | 12·4 | 2·9 | 1·7 | 11·4 | 13·8 |
| [[7](#_ENREF_7)] | French West Indies | 41·4 | 30·8 | – | – | – | – |
| [[8](#_ENREF_8)] | Martinique | 36·2 | 6·3 | – | – | – | – |
| [[9](#_ENREF_9)] | Trinidad and Tobago | 28·2 | 2·6 | – | 0·2 | – | 8·1 |
| [[10](#_ENREF_10)] | France | 0·4 | 0·2 | – | – | – | – |
| [[11](#_ENREF_11)] | Peru | 115·8 | 56·3 | 1·8 | 0·7 | 1·6 | 1·2 |
| [[11](#_ENREF_11)] | Peru | 33·4 | 33·2 | 0 | 0 | 0 | 0 |
| [[12](#_ENREF_12)] | Brazil | 28·9 | 11·8 | 3·1 | 0·9 | 10·7 | 7·3 |
| [[13](#_ENREF_13)] | Brazil | 8·5 | 5·4 | – | 0·1 | – | 1·8 |
| [[14](#_ENREF_14)] | Brazil | 7·42 | 10·3 | – | – | – | – |
| [[15](#_ENREF_15)] | New Caledonia | 885·8 | 251·2 | – | 2·0 | – | 0·8 |
| [[16](#_ENREF_16)] | Hawaii | 272·5 | 32·5 | – | – | – | – |
| [[17](#_ENREF_17)] | Ethiopia | 337·7 | 160·3 | – | – | – | – |
| [[18](#_ENREF_18)] | Cameroon | 104·4 | 69·2 | – | 4·3 | 6·4 | – |

– , No data reported;

**References**

1. Murdoch DR, Woods CW, Zimmerman MD, Dull PM, Belbase RH, et al. The etiology of febrile illness in adults presenting to Patan hospital in Kathmandu, Nepal. Am J Trop Med Hyg. 2004; 70: 670-675. PMID: 15211012

2. Myint KSA, Gibbons RV, Murray CK, Rungsimanphaiboon K, Supornpun W, et al. Leptospirosis in Kamphaeng Phet, Thailand. Am J Trop Med Hyg. 2007; 76: 135. PMID: 17255242

3. Tangkanakul W, Tharmaphornpilas P, Plikaytis BD, Bragg SL, Poonsuksombat D, et al. Risk factors associated with leptospirosis in northeastern Thailand, 1998. Am J Trop Med Hyg. 2000; 63: 204-208. PMID: 11388516

4. Yersin C, Bovet P, Mérien F, Wong T, Panowsky J, et al. Human leptospirosis in the Seychelles (Indian Ocean): a population-based study. Am J Trop Med Hyg. 1998; 59: 933-940. PMID: 9886203

5. Seng H, Sok T, Tangkanakul W, Petkanchanapong W, Kositanont U, et al. Leptospirosis in Takeo Province, Kingdom of Cambodia, 2003. J Med Assoc Thailand. 2007; 90: 546. PMID: 17427534

6. Everard CO, Edwards CN, Everard J, Carrington DG A twelve-year study of leptospirosis on Barbados. Eur J Epidemiol. 1995; 11: 311-320. PMID: 7493664

7. Storck CH, Postic D, Lamaury I, Perez JM. Changes in epidemiology of leptospirosis in 2003--2004, a two El Niño Southern Oscillation period, Guadeloupe archipelago, French West Indies. Epidemiology and infection. 2008; 136: 1407-1415. doi: 10.1017/S0950268807000052 PMID: 18096102

8. L'Homme V, Grolierbois L, Jouannelle J, Elisabeth L. Leptospirose en Martinique de 1987 à 1992 : bilan d'une étude épidémiologique, clinique et biologique. Med Mal Infect. 1996; 26: 94-98.

9. Everard CO, Fraser-Chanpong G, Everard J. The incidence of severe leptospirosis in Trinidad. Trop Geogr Med. 1987; 39: 126. PMID: 3629704

10. Nardone A, Campese C, Capek I, Sanitaire IdV. Les facteurs de risques de leptospirose en France métropolitaine : Une étude cas-témoin, juillet 1999-février 2000 (Surveillance). 2002.

11. Segura ER, Ganoza CA, Campos KJ, Ricaldi JN, Torres S, et al. Clinical spectrum of pulmonary involvement in leptospirosis in a region of endemicity, with quantification of leptospiral burden. Clinical infectious diseases. 2005; 40: 343-351. doi: 10.1086/427110 PMID: 15668855

12. Ko AI Centro de Pesquisas Gonçalo Moniz, Fundação Oswaldo Cruz, Ministério da Saúde, Salvador, Brazil. Grey literature provided by LERG members. 2010.

13. Sarkar U, Nascimento SF, Barbosa R, Martins R, Nuevo H, et al. Population-based case-control investigation of risk factors for leptospirosis during an urban epidemic. Am J Trop Med Hyg. 2002; 66: 605-610. PMID: 12201599

14. Maciel EAP, De Carvalho ALF, Nascimento SF, De Matos RB, Gouveia EL, et al. Household transmission of leptospira infection in urban slum communities. PLoS Negl Trop Dis. 2008; 2: e154. doi 10.1371/journal.pntd.0000154 PMID: 18357340

15. Goarant C. La direction des affaires sanitaires et sociales (DASS) de Nouvelle-Calédonie, New Caledonia, France. Grey literature provided by LERG members. 2010.

16. Sasaki DM, Pang L, Minette HP, Wakida CK, Fujimoto WJ, et al. Active surveillance and risk factors for leptospirosis in Hawaii. Am J Trop Med Hyg. 1993; 48: 35. PMID: 8427386

17. Yimer E, Koopman S, Messele T, Wolday D, Newayeselassie B, et al. Human leptospirosis in Ethiopia: a pilot study in Wonji. Ethiop J Health Dev. 2004; 18: 48-51.

18. Le Bras J, Guyer B, Sulzer C, Mailloux M. [Anademic focus of leptospirosis at Fondem (U.R. of Cameroon)]. B Soc Pathol Exot. 1997; 70: 569-583. PMID: 615682
