# Supplementary material for: Global Morbidity and Mortality of Leptospirosis: A Systematic Review
Source: PLoS Negl Trop Dis. 2015 Sep 17;9(9):e0003898. doi: 10.1371/journal.pntd.0003898 (PMC4574773; doi:10.1371/journal.pntd.0003898)
Supplement: S8 Table — (DOCX) [file pntd.0003898.s011.docx]

S8 Table: Reported disease morbidity, mortality, and case fatality, according to GBD region (A) and WHO sub-region (B).

| **A) GBD Region** | **Morbidity* (N=80)** | | **Mortality* (N=35)** | | **Case fatality (N=35)** | |
| --- | --- | --- | --- | --- | --- | --- |
|  | N (%) | Median (IQR) | N (%) | Median (IQR) | N (%) | Median (IQR) |
| High Income Asia Pacific | 0 | – | 0 | – | 0 | – |
| Central Asia | 0 | – | 0 | – | 0 | – |
| East Asia | 1 (1) | 2·40 (NA) | 0 | – | 0 | – |
| South Asia | 1 (1) | 4·80 (NA) | 0 | – | 0 | – |
| South-East Asia | 10 (13) | 12·35 (5·67 – 23·19) | 6 (17) | 0·62 (0·34 – 5·15) | 6 (17) | 6·78 (4·99 – 8·03) |
| Australasia | 6 (8) | 2·55 (1·30 – 3·20) | 1 (3) | 0 (NA) | 1 (3) | 0 (NA) |
| Caribbean | 14 (18) | 11·98 (6·29 – 16·30) | 7 (19) | 0·44 (0·19 – 1·13) | 7 (20) | 13·82 (5·78 – 16·82) |
| Central Europe | 3 (4) | 0·60 (0·51 – 2·25) | 3 (8) | 0·06 (0·04 – 0·23) | 3 (9) | 8·15 (7·37 – 9·89) |
| Eastern Europe | 5 (6) | 1·00 (0·44 – 1·80) | 0 | – | 0 | – |
| Western Europe | 15 (19) | 0·50 (0·21 – 0·74) | 6 (17) | 0·09 (0·05 – 0·09) | 5 (14) | 11·90 (10·70 – 14·29) |
| Andean Latin America | 3 (4) | 33·15 (17·17 – 44·70) | 3 (8) | 0·34 (0.17 – 0·51) | 2 (6) | 0·78 (0·39 – 1·17) |
| Central Latin America | 2 (3) | 0·50 (0·31 – 0·68) | 1 (3) | 0 (NA) | 1 (3) | 0 (NA) |
| Southern Latin America | 0 | – | 0 | – | 0 | – |
| Tropical Latin America | 10 (13) | 12·14 (5·93 – 21·02) | 4 (11) | 0·75 (0·51 – 0·93) | 4 (11) | 3·58 (1·58 – 5·71) |
| North Africa / Middle East | 0 | – | 0 | – | 0 | – |
| High Income North America | 0 | – | 0 | – | 0 | – |
| Oceania | 8 (10) | 159·31 (57·91 – 274·56) | 5 (14) | 1·67 (1·66 – 2·00) | 5 (14) | 0·80 (4·85 – 4·66) |
| Central Sub-Saharan Africa | 0 | – | 0 | – | 0 | – |
| East Sub-Saharan Africa | 1 (1) | 160·30 (NA) | 0 | – | 0 | – |
| Southern Sub-Saharan Africa | 0 | – | 0 | – | 0 | – |
| West Sub-Saharan Africa | 1 (1) | 69·23 (NA) | 1 (3) | 4·29 (NA) | 1 (3) | 6·38 (NA) |

| **B) WHO Sub-region**^a^ | **Morbidity* (N=80)** | | **Mortality* (N=35)** | | **Case fatality (N=35)** | |
| --- | --- | --- | --- | --- | --- | --- |
|  | N (%) | Median (IQR) | N (%) | Median (IQR) | N (%) | Median (IQR) |
| AFR-D | 3 (4) | 69·2 (66·1 – 85·1) | 3 (9) | 6·6 (5·5 – 7·4) | 3 (9) | 8·0 (7·2 – 8·4) |
| AFR-E | 1 (1) | 160·3 (160·3 – 160·3) | – |  | – |  |
| AFR-Colonies and Territories^b^ | 3 (4) | 15·8 (10·0 – 20·8) | 2 (6) | 0·6 (0·5 – 0·8) | 2 (6) | 6·8 (6·2 – 7·4) |
| AMR-A | 4 (5) | 20·6 (15·1 – 27·9) | 1 (3) | 0·2 (0·2 – 0·2) | 1 (3) | 0·7 (0·7 – 0·7) |
| AMR-B | 17 (21) | 11·8 (2·6 – 12·5) | 9 (26) | 0·6 (0·2 – 1·1) | 9 (26) | 5·2 (2·0 – 8·2) |
| AMR-D | 3 (4) | 33·2 (17·2 – 44·8) | 2 (6) | 0·4 (0·2 – 0·5) | 2 (6) | 0·8 (0·4 – 1·2) |
| AMR-Colonies and Territories^c^ | 6 (8) | 6·3 (3·8 – 24·7) | 3 (9) | 0·5 (0·3 – 1·1) | 3 (9) | 17·6 (11·9 – 19·7) |
| EMR-B | – |  | – |  | – |  |
| EMR-D | – |  | – |  | – |  |
| EUR-A | 16 (20) | 0·5 (0·2 – 0·8) | 6 (17) | 0·1 (0·0 – 0·1) | 6 (17) | 11·3 (8·8 – 13·7) |
| EUR-B | 2 (3) | 0·5 (0·5 – 0·6) | 2 (6) | 0·1 (0·0 – 0·1) | 2 (6) | 9·1 (7·9 – 10·4) |
| EUR-C | 5 (6) | 1·0 (0·4 – 1·8) | – |  | – |  |
| SEAR-B | 4 5) | 8·5 (5·9 – 10·9) | 2 (6) | 0·4 (0·3 – 0·4) | 2 (6) | 4·0 (3·6 – 4·4) |
| SEAR-D | 1 (1) | 4·8 (4·8 – 4·8) | – |  | – |  |
| WPR-A | 5 (6) | 1·9 (1·1 – 3·2) | 1 (3) | 0·0 (0·0 – 0·0) | 1 (3) | 0·0 (0·0 – 0·0) |
| WPR-B | 2 (3) | 3·8 (3·1 – 4·4) | – |  | – |  |
| WPR-Colonies and Territories^d^ | 8 (10) | 200·3 (82·1 – 274·6) | 4 (11) | 1·9 (1·3 – 2·3) | 4 (11) | 0·7 (0·4 – 1·8) |

IQR, intraquartile range; SD, standard deviation; –, Data unavailable; NA, not applicable. *Annual morbidity and mortality rates in cases or deaths per 100 000 population determined by reported laboratory-confirmed cases. ^a^ WHO sub-region mortality strata: A, very low child, low adult; B, low child, low adult; C, low child, high adult; D, high child, high adult; E, high child, very high adult. ^b^ Includes non-independent colonies and territories in AFR region: Mayotte, Reunion. ^c^ Includes non-independent colonies, states, and territories in AMR region: American Virgin Islands, Anguilla, Bermuda, British Virgin Islands, Cayman Islands, French Guiana, Guadeloupe, Guam, Hawaii, Martinique, Monseratte, Netherlands Antilles, Puerto Rico, St Pierre and Miquelon, Turks and Caicos. ^d^ Includes non-independent colonies and territories in WPR region: French Polynesia, Hong Kong, Macao, New Caledonia, Taiwan, Wallis and Futuna.
