# Supplementary material for: Global Morbidity and Mortality of Leptospirosis: A Systematic Review
Source: PLoS Negl Trop Dis. 2015 Sep 17;9(9):e0003898. doi: 10.1371/journal.pntd.0003898 (PMC4574773; doi:10.1371/journal.pntd.0003898)
Supplement: S9 Table — (DOCX) [file pntd.0003898.s012.docx]

**S9 Table: Model parameters for leptospirosis morbidity and mortality estimation.**

| Demographic group (*d*) | Tropical island | Urbanization  (% of population) | Distance from the equator (degrees latitude of the country centroid) | Life expectancy at birth (years) |
| --- | --- | --- | --- | --- |
|  | ß Coefficient (standard error) | | | |
| Morbidity |  |  |  |  |
| Females 0-9 | 1·5071 (0·6863) ** | -0·0226 (0·0125) * | -0·0458 (0·0215) ** | 0·0026 (0·0141) |
| Females 10-19 | 1·3152 (0·7046) * | -0·0262 (0·0129) * | -0·0503 (0·0221) ** | 0·0354 (0·0145) ** |
| Females 20-29 | 1·2501 (0·7123) * | -0·0274 (0·0130) ** | -0·0518 (0·0224) ** | 0·0465 (0·0147) *** |
| Females 30-39 | 1·2317 (0·7146) * | -0·0278 (0·0130) ** | -0·0522 (0·0224) ** | 0·0497 (0·0147) *** |
| Females 40-49 | 1·2431 (0·7132) * | -0·0276 (0·0130) ** | -0·0520 (0·0224) ** | 0·0477 (0·0147) *** |
| Females 50-59 | 1·2327 (0·7145) * | -0·0277 (0·0130) ** | -0·0522 (0·0224) ** | 0·0495 (0·0147) *** |
| Females 60-69 | 1·2247 (0·7155) * | -0·0279 (0·0131) ** | -0·0524 (0·0225) ** | 0·0509 (0·0147) *** |
| Females 70+ | 1·2851 (0·7081) * | -0·0278 (0·0129) ** | -0·0510 (0·0222) ** | 0·0406 (0·0146) *** |
| Males 0-9 | 1·3121 (0·7050) * | -0·0263 (0·0129) * | -0·0504 (0·0221) ** | 0·0359 (0·0145) ** |
| Males 10-19 | 1·1556 (0·7248) | -0·0292 (0·0132) ** | -0·0540 (0·0228) ** | 0·0627 (0·0149) **** |
| Males 20-29 | 1·1063 (0·7319) | -0·0301 (0·0134) ** | -0·0552 (0·0230) ** | 0·0712 (0·0151) **** |
| Males 30-39 | 1·1142 (0·7307) | -0·0300 (0·0133) ** | -0·0550 (0·0229) ** | 0·0698 (0·0150) **** |
| Males 40-49 | 1·1256 (0·7290) | -0·0297 (0·0133) ** | -0·0547 (0·0133) ** | 0·0679 (0·0150) **** |
| Males 50-59 | 1·1282 (0·7287) | -0·0297 (0·0133) ** | -0·0547 (0·0229) ** | 0·0674 (0·0150) **** |
| Males 60-69 | 1·1465 (0·7261) | -0·0294 (0·0133) ** | -0·0542 (0·0228) ** | 0·0643 (0·0150) **** |
| Males 70+ | 1·1851 (0·7207) | -0·0290 (0·0132) ** | -0·0533 (0·0226) ** | 0·0577 (0·0148) **** |
| Mortality |  |  |  |  |
| Females 0-9 | 1·5118 (0·6862) ** | -0·0228 (0·0125) * | -0·0487 (0·0215) ** | -0·0111 (0·0141) |
| Females 10-19 | 1·4635 (0·6897) ** | -0·0237 (0·0126) * | -0·0498 (0·0217) ** | -0·0029 (0·0142) |
| Females 20-29 | 1·5199 (0·6857) ** | -0·0227 (0·0125) * | -0·0485 (0·0215) ** | -0·0125 (0·0141) |
| Females 30-39 | 1·5723 (0·6824) ** | -0·0217 (0·0125) * | -0·0473 (0·0214) ** | -0·0215 (0·0141) |
| Females 40-49 | 1·3790 (0·6969) * | -0·0253 (0·0127) * | -0·0518 (0·0219) ** | 0·0116 (0·0143) |
| Females 50-59 | 1·3827 (0·6966) * | -0·0252 (0·0127) * | -0·0517 (0·0219) ** | 0·0110 (0·0143) |
| Females 60-69 | 1·3839 (0·6965) * | -0·0252 (0·0127) * | -0·0517 (0·0219) ** | 0·0108 (0·0143) |
| Females 70+ | 1·3571 (0·6990) * | -0·0257 (0·0128) * | -0·0523 (0·0219) ** | 0·0154 (0·0144) |
| Males 0-9 | 1·4067 (0·6944) * | -0·0248 (0·0127) * | -0·0511 (0·0218) ** | 0·0069 (0·0143) |
| Males 10-19 | 1·4216 (0·6931) ** | -0·0245 (0·0127) * | -0·0508 (0·0218) ** | 0·0044 (0·0143) |
| Males 20-29 | 1·3264 (0·7021) * | -0·0263 (0·0128) ** | -0·0530 (0·0220) ** | 0·0206 (0·0145) |
| Males 30-39 | 1·3217 (0·7026) * | -0·0263 (0·0128) ** | -0·0531 (0·0221) ** | 0·0214 (0·0145) |
| Males 40-49 | 1·2236 (0·7136) * | -0·0282 (0·0130) ** | -0·0554 (0·0224) ** | 0·0382 (0·0147) ** |
| Males 50-59 | 1·2164 (0·7144) * | -0·0283 (0·0130) ** | -0·0556 (0·0224) ** | 0·0395 (0·0147) ** |
| Males 60-69 | 1·2311 (0·7127) * | -0·0280 (0·0130) ** | -0·0552 (0·0224) ** | 0·0370 (0·0147) ** |
| Males 70+ | 1·2569 (0·7096) * | -0·0275 (0·0130) ** | -0·0547 (0·0223) ** | 0·0325 (0·0146) ** |

The model takes the form of $\log\left( \left\{ EI,EM \right\}_{cd} \right)\approx0+\beta_{1c}\left( X_{1} \right)+\beta_{2c}\left( X_{2} \right)+\beta_{3c}\left( X_{3} \right)+\beta_{4c}\left( X_{4} \right)$ where ${EI}_{cd}$and ${EM}_{cd}$ are the estimated demographic-specific morbidity and mortality, respectively, in one of 16 age and gender groups *d* in country *c*. Stars indicate statistical significance of each regressor. *, p<0·05. **, p<0·01. ***, p<0·001. ****, p<0.0001.
