# Supplementary material for: Global Morbidity and Mortality of Leptospirosis: A Systematic Review
Source: PLoS Negl Trop Dis. 2015 Sep 17;9(9):e0003898. doi: 10.1371/journal.pntd.0003898 (PMC4574773; doi:10.1371/journal.pntd.0003898)
Supplement: S10 Table — (DOCX) [file pntd.0003898.s013.docx]

**S10 Table: Equations.**

Equations are listed in the order they are referenced by manuscript Fig 2.

Equation 1: Calculation of mean under-reporting ratio for cases and deaths

$$\left\{ \bar{URI},\bar{URM} \right\}=\sum_{i=1}^{n} w_{i}\left( \frac{\left\{ SI,SM \right\}_{i}}{\left\{ LI,LM \right\}_{i}} \right)$$

Where for each study $i\equiv j$ with information on both clinically suspected and laboratory-confirmed cases (*i* = 19) and deaths (*i* = 4):

- $\left\{ SI,SM \right\}_{i}$ = clinically suspected morbidity or mortality, with or without laboratory confirmation (sum of all suspected cases or deaths $N$ divided by study population)
- $\left\{ LI,LM \right\}_{i}$ = laboratory-confirmed morbidity or mortality (laboratory-confirmed cases or deaths divided by study population)
- $w_{i}$ = $\frac{N_{i}}{\sum_{j=1}^{n} N_{j}}$

Equation 2: Calculation of relative risk for membership in each of 16 demographic groups among all cases or deaths, based on *i* studies with appropriately stratified data (*i*=10 studies for cases, and *i*=3 studies for deaths)

$$\left\{ \bar{RRI},\bar{RRM} \right\}_{d}=\sum_{i=1}^{n} w_{i}\left( \frac{\left\{ DI,DM \right\}_{di}}{\left\{ DI,DM \right\}_{i}} \right)$$

Where for each demographic *d*, and study $i\equiv j$:

- $\left\{ DI,DM \right\}_{di}$ = demographic-specific morbidity or mortality (demographic-specific cases or deaths divided by demographic-specific population)
- $\left\{ DI,DM \right\}_{i}$ = total morbidity DI or mortality DM (sum of all demographic-specified cases or deaths $N$, divided by total study population)
- $w_{i}$ = $\frac{N_{i}}{\sum_{j=1}^{n} N_{j}}$

Equation 3: Disaggregation of mean crude reported morbidity $\bar{I}_{c}$ or estimated crude mortality $\bar{M}_{c}$ into demographic-specific estimates ${EI}_{cd}$ and ${EM}_{cd}$*.*

$$\left\{ EI,EM \right\}_{cd}=\left\{ \bar{I},\bar{M} \right\}_{c}\times\left\{ \bar{RRI},\bar{RRM} \right\}_{d}\times\frac{\left\{ \bar{I},\bar{M} \right\}_{c}}{\left\{ FI,FM \right\}_{c}}$$

Where for each country *c* and demographic *d*:

- $\bar{I}_{c}$ = mean of crude all-ages morbidity or mortality reported by studies in each country *c*, weighted by study size.
- $\bar{M}_{c}$ = Estimated mean crude reported mortality, using mean crude reported morbidity and global mean case fatality (Equation 7).
- $\left\{ FI,FM \right\}_{c}$ = Unadjusted fractional crude morbidity or mortality, $\frac{\sum_{d=1}^{n} \left( \left\{ \bar{I},\bar{M} \right\}_{c}\times\left\{ \bar{RRI},\bar{RRM} \right\}_{d}\times P_{cd} \right)}{\sum_{d=1}^{n} P_{cd}}$
- $P_{cd}$= demographic-specific population in each country*.*

Equation 4: Multiple linear regression model to predict the log-transformation of demographic-specific morbidity and mortality.

$$\log\left( \left\{ EI,EM \right\}_{cd} \right)\approx0+\beta_{1d}\left( X_{1c} \right)+\beta_{2d}\left( X_{2c} \right)+\beta_{3d}\left( X_{3c} \right)+\beta_{4d}\left( X_{4c} \right)+\varepsilon$$

Where for each demographic *d* and country *c*:

- $X_{1}$ = Tropical island
- $X_{2}$ = Urbanization of population (percent)
- $X_{3}$ = Distance from the equator (degrees latitude of country’s geographic centroid)
- $X_{4}$ = Life expectancy at birth (years)

Equation 5: Transformation of raw model predictions from the log scale to calculate predicted demographic-specific morbidity $\hat{I}_{cd}$ and mortality $\hat{M}_{cd}$ , and respective standard errors ${\hat{I}SE}_{cd}$ and ${\hat{M}SE}_{cd}$.

$$\left\{ \hat{I},\hat{M} \right\}_{cd}= exp\left( \left\{ \tilde{I},\tilde{M} \right\}_{cd}+\frac{{\left\{ \tilde{I}SE,\tilde{M}SE \right\}_{cd}}^{2}}{2} \right)$$

$$\left\{ \hat{I}SE,\hat{M}SE \right\}_{cd}=\sqrt{\left( exp\left( {\left\{ \tilde{I}SE,\tilde{M}SE \right\}_{cd}}^{2} \right)-1 \right)\times exp\left( 2\times\left\{ \tilde{I},\tilde{M} \right\}_{cd}+{\left\{ \tilde{I}SE,\tilde{M}SE \right\}_{cd}}^{2} \right)}$$

Where for each demographic *d* and country *c*:

- $\left\{ \tilde{I},\tilde{M} \right\}_{cd}$ = Predicted value for log of morbidity or mortality from Equation 4
- $\left\{ \tilde{I}SE,\tilde{M}SE \right\}_{cd}$ = Standard error of the prediction for log morbidiy or mortality

Equation 6: Generate a stochastic estimate of the morbidity and mortality of leptospirosis, including under-reporting ratio, using a Monte Carlo simulation model.

For each country *c* and demographic *d*,

1) For j=1,…1000, select $\left\{ XI,XM \right\}_{cdj}$ independent log-normally distributed samples with mean $\left\{ \hat{I},\hat{M} \right\}_{cd}$ and standard error $\left\{ \hat{I}SE,\hat{M}SE \right\}_{cd}$ (Equation 5).

2) For j=1,…1000, select $\left\{ YI,YM \right\}_{cdj}$ independent normally distributed samples with mean $\left\{ \bar{URI},\bar{URM} \right\}$ and standard error of that mean (Equation 1).

3) Multiply $\left\{ XI,XM \right\}_{cdj}\times\left\{ YI,YM \right\}_{cdj}$ to obtain 1000 samples for morbidity and mortality in each country and demographic group

4) To obtain final predictions for morbidity $I_{cd}^{*}$ and mortality $M_{cd}^{*}$, calculate mean and 95% uncertainty limits of $\left\{ XI,XM \right\}_{cdj}\left\{ YI,YM \right\}_{cdj}$

5) Aggregate country, regional, and world estimates for $I^{*}$ and $M^{*}$ were calculated using published population data.

Equation 7: Estimation of mean crude reported mortality $\bar{M}_{c}$, using mean crude reported morbidity $\bar{I}_{c}$ and global mean case fatality ratio $\bar{F}$.

$\bar{M}_{c}=\bar{I}_{c}\times\bar{F}$
