# Supplementary material for: Global Morbidity and Mortality of Leptospirosis: A Systematic Review
Source: PLoS Negl Trop Dis. 2015 Sep 17;9(9):e0003898. doi: 10.1371/journal.pntd.0003898 (PMC4574773; doi:10.1371/journal.pntd.0003898)
Supplement: S11 Table — (DOCX) [file pntd.0003898.s014.docx]

**S11 Table: Estimated annual leptospirosis morbidity and mortality by WHO sub-region.**

| Region | Morbidity^a^ | Cases | Mortality^a^ | Deaths |
| --- | --- | --- | --- | --- |
|  | Estimate (95% Confidence interval of the prediction) | | | |
| **All WHO Sub-regions**^b^ | **14·77 (4·38 – 25·03)** | **1 030 000 (434 000 – 1 750 000)** | **0·84 (0·34 – 1·37)** | **58 900 (23 800 – 95 900)** |
| Africa D^c^ | 9·69 (3·70 – 15·97) | 38 400 (14 600 – 63 200) | 0·79 (0·27 – 1·30) | 3100 (1100 – 5100) |
| Africa E | 22·15 (8·29 – 37·56) | 99 500 (37 200 – 169 000) | 1·68 (0·69 – 2·70) | 7600 (3100 – 12 100) |
| Americas A^d^ | 5·34 (1·85 – 9·46) | 19 800 (5900 – 35 200) | 0·26 (0·11 – 0·44) | 1000 (400 – 1700) |
| Americas B^d^ | 15·11 (5·34 – 25·35) | 73 900 (26 100 – 124 000) | 0·70 (0·28 – 1·17) | 3400 (1400 – 5900) |
| Americas D | 22·33 (7·51 – 37·87) | 18 700 (6300 – 31 700) | 1·08 (0·37 – 1·80) | 900 (300 – 1500) |
| Eastern Mediterranean B | 5·82 (2·11 – 9·69) | 9900 (3600 – 16 400) | 0·25 (0·09 – 0·43) | 400 (200 – 700) |
| Eastern Mediterranean D | 9·21 (3·17 – 15·72) | 40 500 (14 000 – 69 000) | 0·55 (0·22 – 0·89) | 2400 (1000 – 3900) |
| Europe A | 3·90 (1·36 – 6·54) | 16 900 (5900 – 28 400) | 0·18 (0·06 – 0·29) | 800 (300 – 1200) |
| Europe B | 4·49 (1·61 – 7·39) | 10 400 (3700 – 17 100) | 0·22 (0·09 – 0·36) | 500 (200 – 800) |
| Europe C | 1·55 (0·52 – 2·69) | 3600 (1200 – 6200) | 0·10 (0·04 – 0·17) | 200 (100 – 400) |
| South-East Asia B | 56·06 (19·72 – 99·06) | 181 000 (64 000 – 319 000) | 3·16 (1·32 – 0·01) | 10 100 (4200 – 17 400) |
| South-East Asia D | 19·06 (6·53 – 34·36) | 283 000 (97 000 – 510 000) | 1·09 (0·37 – 1·84) | 16 100 (5600 – 27 200) |
| Western Pacific A | 6·95 (2·43 – 12·40) | 11 000 (3800 – 20 000) | 0·32 (0·12 – 0·55) | 500 (200 – 900) |
| Western Pacific B^e^ | 14·36 (5·09 – 24·49) | 241 000 (85 400 – 411 000) | 0·68 (0·27 – 1·15) | 11 400 (4600 – 19 300) |

^a^ Annual morbidity and mortality from leptospirosis is shown as cases or deaths per 100 000 population. ^b^ WHO sub-region mortality strata: A, very low child, low adult; B, low child, low adult; C, low child, high adult; D, high child, high adult; E, high child, very high adult. ^c^ Includes non-independent colonies and territories in AFR region: Mayotte, Reunion. ^d^ Includes non-independent colonies, states, and territories in AMR region: American Virgin Islands, Anguilla, Bermuda, British Virgin Islands, Cayman Islands, French Guiana, Guadeloupe, Guam, Hawaii, Martinique, Monseratte, Netherlands Antilles, Puerto Rico, St Pierre and Miquelon, Turks and Caicos. ^e^ Includes non-independent colonies and territories in WPR region: French Polynesia, Hong Kong, Macao, New Caledonia, Taiwan, Wallis and Futuna
