# Supplementary material for: Global Morbidity and Mortality of Leptospirosis: A Systematic Review
Source: PLoS Negl Trop Dis. 2015 Sep 17;9(9):e0003898. doi: 10.1371/journal.pntd.0003898 (PMC4574773; doi:10.1371/journal.pntd.0003898)
Supplement: S12 Table — (DOCX) [file pntd.0003898.s015.docx]

**S12 Table: Estimated leptospirosis morbidity and mortality by country, grouped according to WHO sub-region.**

| **Country** | **Morbidity^a^ (95% CI)** | **Mortality^a^ (95% CI)** |
| --- | --- | --- |
| **Africa region, stratum D (AFR D)** | | |
| Algeria | 7·19 (2·64 – 13·02) | 0·33 (0·12 – 0·56) |
| Angola | 3·58 (1·26 – 6·15) | 0·43 (0·15 – 0·73) |
| Benin | 13·26 (4·73 – 23·61) | 0·96 (0·31 – 1·68) |
| Burkina Faso | 12·47 (4·52 – 22·60) | 1·09 (0·36 – 1·86) |
| Cameroon | 9·39 (3·23 – 16·56) | 0·82 (0·30 – 1·46) |
| Cape Verde | 44·01 (14·62 – 79·55) | 2·18 (0·76 – 4·00) |
| Chad | 7·25 (2·63 – 12·31) | 0·81 (0·28 – 1·31) |
| Comoros | 74·31 (26·97 – 138·97) | 5·43 (1·83 – 9·79) |
| Equatorial Guinea | 18·02 (6·07 – 33·84) | 1·59 (0·53 – 2·88) |
| Gabon | 9·63 (3·02 – 20·20) | 0·69 (0·23 – 1·34) |
| Gambia | 8·07 (2·83 – 13·85) | 0·58 (0·21 – 0·99) |
| Ghana | 17·73 (6·45 – 32·22) | 1·07 (0·37 – 1·94) |
| Guinea | 15·36 (5·55 – 26·67) | 1·22 (0·42 – 2·10) |
| Guinea-Bissau | 9·67 (3·50 – 16·71) | 0·97 (0·34 – 1·62) |
| Liberia | 8·10 (2·79 – 14·64) | 0·70 (0·23 – 1·21) |
| Madagascar | 12·29 (4·40 – 20·80) | 0·78 (0·27 – 1·33) |
| Mali | 6·60 (2·25 – 11·19) | 0·64 (0·22 – 1·08) |
| Mauritania | 8·54 (2·91 – 14·94) | 0·59 (0·21 – 1·01) |
| Mauritius | 81·14 (27·42 – 154·71) | 4·37 (1·54 – 7·94) |
| Niger | 11·93 (3·91 – 20·85) | 1·04 (0·38 – 1·78) |
| Nigeria | 6·72 (2·32 – 11·56) | 0·69 (0·24 – 1·14) |
| Sao Tome | 58·86 (20·59 – 107·14) | 4·21 (1·33 – 7·65) |
| Senegal | 11·73 (4·10 – 20·59) | 0·76 (0·25 – 1·29) |
| Seychelles | 126·64 (43·61 – 234·03) | 7·01 (2·28 – 13·16) |
| Sierra Leone | 9·57 (3·30 – 16·12) | 0·94 (0·32 – 1·56) |
| Togo | 17·97 (5·88 – 31·42) | 1·18 (0·39 – 2·03) |
| Non-independent colonies, states, and territories | |  |
| *Mayotte* | 199·19 (64·06 – 386·05) | 64·06 (386·05 – 7·38) |
| *Reunion* | 31·79 (10·60 – 63·70) | 10·60 (63·70 – 1·39) |
| **Africa region, stratum E (AFR E)** | | |
| Botswana | 5·52 (1·89 – 9·58) | 0·35 (0·15 – 0·58) |
| Burundi | 33·40 (11·30 – 61·92) | 2·87 (1·09 – 5·18) |
| Central African Republic | 9·87 (3·37 – 16·94) | 1·05 (0·43 – 1·75) |
| Congo | 11·46 (4·15 – 22·36) | 0·99 (0·40 – 1·75) |
| Cote d'Ivoire | 11·13 (3·91 – 19·76) | 0·90 (0·36 – 1·55) |
| Democratic Republic of the Congo | 16·75 (5·65 – 31·65) | 1·63 (0·63 – 2·82) |
| Eritrea | 32·18 (10·84 – 61·14) | 1·48 (0·54 – 2·76) |
| Ethiopia | 34·99 (11·72 – 66·84) | 2·29 (0·90 – 4·12) |
| Kenya | 39·46 (13·04 – 79·16) | 2·89 (1·04 – 5·25) |
| Lesotho | 4·24 (1·51 – 7·70) | 0·44 (0·17 – 0·75) |
| Malawi | 18·17 (6·35 – 32·86) | 1·46 (0·58 – 2·50) |
| Mozambique | 7·54 (2·77 – 12·58) | 0·69 (0·29 – 1·15) |
| Namibia | 12·65 (4·62 – 21·72) | 0·71 (0·29 – 1·19) |
| Rwanda | 50·27 (15·60 – 108·97) | 3·09 (1·14 – 5·80) |
| South Africa | 2·83 (1·07 – 5·03) | 0·26 (0·10 – 0·44) |
| Swaziland | 5·50 (1·99 – 9·93) | 0·54 (0·22 – 0·91) |
| Uganda | 35·67 (11·57 – 70·67) | 2·79 (1·05 – 5·13) |
| United Republic of Tanzania | 20·89 (7·27 – 38·34) | 1·69 (0·64 – 2·90) |
| Zambia | 6·12 (2·25 – 10·48) | 0·64 (0·26 – 1·04) |
| Zimbabwe | 3·65 (1·31 – 6·13) | 0·47 (0·19 – 0·77) |
| **Americas region, stratum A (AMR A)** | | |
| Canada | 1·89 (0·47 – 3·78) | 0·09 (0·03 – 0·18) |
| Cuba | 36·43 (10·71 – 70·85) | 2·09 (0·83 – 3·75) |
| USA | 3·81 (1·12 – 6·98) | 0·19 (0·07 – 0·34) |
| Non-independent colonies, states, and territories | |  |
| *Bermuda* | 4·07 (1·34 – 7·82) | 0·20 (0·07 – 0·37) |
| *British Virgin Islands* | 123·19 (41·68 – 228·55) | 5·88 (2·24 – 10·68) |
| *Cayman Islands* | 26·43 (8·35 – 50·46) | 1·40 (0·48 – 2·90) |
| *French Guiana* | 34·33 (10·77 – 76·06) | 1·22 (0·40 – 2·52) |
| *Guam* | 48·21 (14·90 – 94·83) | 2·14 (0·77 – 3·93) |
| *Hawii* | 37·43 (12·58 – 68·35) | 2·06 (0·75 – 3·72) |
| *Martinique* | 49·14 (17·87 – 90·95) | 2·41 (0·86 – 4·42) |
| *Netherlands Antilles* | 46·09 (15·30 – 86·08) | 2·40 (0·93 – 4·48) |
| *Puerto Rico* | 36·11 (11·60 – 68·43) | 1·70 (0·65 – 3·31) |
| *St Pierre Miqueleon* | 2·33 (0·72 – 4·53) | 0·12 (0·04 – 0·23) |
| *American Virgin Islands* | 123·19 (41·68 – 228·55) | 5·88 (2·24 – 10·68) |
| *Total (non-independent territories)* | 39·13 (13·71 – 67·20) | 1·90 (0·76 – 3·25) |
| **Americas region, stratum B (AMR B)** | | |
| Antigua and Barbuda | 137·27 (43·33 – 267·49) | 6·68 (2·59 – 12·23) |
| Argentina | 3·34 (1·14 – 6·23) | 0·16 (0·06 – 0·28) |
| Bahamas | 23·36 (7·27 – 46·23) | 1·23 (0·44 – 2·32) |
| Barbados | 146·92 (51·59 – 276·84) | 8·12 (3·19 – 15·26) |
| Belize | 19·02 (6·80 – 34·59) | 0·81 (0·30 – 1·39) |
| Brazil | 13·77 (4·72 – 27·09) | 0·65 (0·24 – 1·24) |
| Chile | 5·37 (1·87 – 9·56) | 0·24 (0·09 – 0·44) |
| Colombia | 27·93 (8·68 – 58·05) | 1·22 (0·40 – 2·51) |
| Costa Rica | 38·06 (12·92 – 74·16) | 1·48 (0·54 – 2·76) |
| Dominica | 42·40 (14·75 – 79·03) | 2·45 (0·97 – 4·31) |
| Dominican Republic | 38·60 (13·26 – 72·12) | 2·05 (0·82 – 3·72) |
| El Salvador | 15·40 (5·49 – 27·77) | 0·70 (0·26 – 1·24) |
| Grenada | 131·53 (46·00 – 243·50) | 7·02 (2·69 – 12·83) |
| Guyana | 55·33 (17·46 – 108·36) | 3·24 (1·18 – 6·54) |
| Honduras | 18·61 (6·28 – 33·19) | 0·83 (0·33 – 1·45) |
| Jamaica | 57·68 (19·64 – 100·48) | 3·18 (1·25 – 5·44) |
| Mexico | 8·61 (3·08 – 15·50) | 0·37 (0·14 – 0·67) |
| Panama | 29·49 (9·47 – 56·95) | 1·24 (0·44 – 2·37) |
| Paraguay | 12·50 (4·10 – 22·03) | 0·52 (0·20 – 0·88) |
| Saint Kitts and Nevis | 132·71 (43·10 – 246·48) | 7·00 (2·57 – 13·05) |
| Saint Lucia | 193·16 (63·53 – 373·98) | 8·65 (3·34 – 16·51) |
| Saint Vincent and the Grenadines | 107·87 (34·85 – 203·93) | 5·87 (2·19 – 10·15) |
| Suriname | 26·64 (8·68 – 54·19) | 1·37 (0·51 – 2·66) |
| Trinidad and Tobago | 300·48 (95·79 – 607·75) | 16·00 (6·07 – 32·15) |
| Uruguay | 3·08 (1·03 – 5·60) | 0·16 (0·06 – 0·28) |
| Venezuela, Bolivarian Republic of | 15·48 (4·58 – 33·41) | 0·69 (0·25 – 1·32) |
| Non-independent colonies, states, and territories | |  |
| *Anguilla* | 28·84 (10·05 – 58·10) | 1·41 (0·50 – 2·94) |
| *Guadelope* | 36·19 (13·12 – 75·13) | 1·71 (0·60 – 3·41) |
| *Monseratte* | 220·88 (71·54 – 462·80) | 10·18 (3·47 – 20·18) |
| *Turks and Caicos^d^* | 23·36 (7·27 – 46·23) | 1·23 (0·44 – 2·32) |
| **Americas region, stratum D (AMR D)** | | |
| Bolivia | 8·94 (3·14 – 15·93) | 0·48 (0·17 – 0·83) |
| Ecuador | 35·69 (11·92 – 71·02) | 1·62 (0·50 – 3·12) |
| Guatemala | 16·77 (5·96 – 29·96) | 0·76 (0·26 – 1·32) |
| Haiti | 32·47 (10·79 – 59·80) | 2·30 (0·80 – 4·10) |
| Nicaragua | 23·45 (8·00 – 44·34) | 0·91 (0·30 – 1·64) |
| Peru | 19·63 (6·38 – 38·68) | 0·79 (0·25 – 1·53) |
| **Eastern Mediterranean region, stratum B (EMR B)** | | |
| Bahrain | 4·52 (1·37 – 9·18) | 0·20 (0·06 – 0·42) |
| Cyprus | 9·45 (3·31 – 16·19) | 0·39 (0·13 – 0·69) |
| Iran, Islamic Republic of | 5·47 (2·02 – 9·70) | 0·24 (0·08 – 0·43) |
| Jordan | 3·64 (1·28 – 6·63) | 0·16 (0·06 – 0·29) |
| Kuwait | 4·86 (1·56 – 9·68) | 0·19 (0·05 – 0·37) |
| Lebanon | 2·93 (0·92 – 5·37) | 0·15 (0·05 – 0·26) |
| Libyan Arab Jamahiriya | 6·63 (2·11 – 12·33) | 0·29 (0·10 – 0·52) |
| Occupied Palestine | 3·11 (1·07 – 5·66) | 0·13 (0·05 – 0·23) |
| Oman | 10·65 (3·85 – 19·64) | 0·44 (0·14 – 0·79) |
| Qatar | 6·44 (1·96 – 13·83) | 0·26 (0·08 – 0·56) |
| Saudi Arabia | 5·68 (1·95 – 10·41) | 0·27 (0·09 – 0·48) |
| Syrian Arab Republic | 6·56 (2·19 – 12·13) | 0·26 (0·09 – 0·46) |
| Tunisia | 6·41 (2·28 – 11·53) | 0·27 (0·09 – 0·49) |
| United Arab Emirates | 11·37 (3·44 – 22·33) | 0·40 (0·12 – 0·75) |
| **Eastern Mediterranean region, stratum D (EMR D)** | | |
| Afghanistan | 2·81 (0·95 – 4·96) | 0·37 (0·15 – 0·65) |
| Djibouti | 5·52 (1·90 – 10·30) | 0·41 (0·16 – 0·75) |
| Egypt | 11·65 (4·25 – 20·62) | 0·56 (0·22 – 1·00) |
| Iraq | 2·71 (0·95 – 4·79) | 0·17 (0·07 – 0·29) |
| Morocco | 7·19 (2·55 – 12·90) | 0·33 (0·13 – 0·57) |
| Pakistan | 8·32 (2·86 – 15·14) | 0·50 (0·20 – 0·86) |
| Somalia | 11·39 (4·12 – 20·25) | 1·20 (0·49 – 2·01) |
| Sudan | 11·31 (4·09 – 19·92) | 0·84 (0·35 – 1·40) |
| Yemen | 21·78 (6·83 – 40·94) | 1·11 (0·42 – 1·95) |
| **Europe region, stratum A (EUR A)** | | |
| Andorra | 3·54 (1·13 – 6·51) | 0·15 (0·05 – 0·30) |
| Azores | 6·55 (2·21 – 11·60) | 0·35 (0·12 – 0·64) |
| Austria | 4·53 (1·57 – 8·27) | 0·21 (0·07 – 0·38) |
| Belgium | 1·59 (0·52 – 3·49) | 0·08 (0·02 – 0·15) |
| Croatia | 5·34 (1·71 – 9·73) | 0·27 (0·09 – 0·49) |
| Czech Republic | 2·78 (0·90 – 5·31) | 0·14 (0·04 – 0·26) |
| Denmark | 2·42 (0·76 – 4·83) | 0·12 (0·04 – 0·22) |
| Finland | 2·31 (0·72 – 4·75) | 0·11 (0·03 – 0·24) |
| France | 3·67 (1·28 – 6·85) | 0·16 (0·05 – 0·29) |
| Germany | 3·04 (1·00 – 5·81) | 0·15 (0·05 – 0·30) |
| Greece | 5·58 (1·99 – 9·79) | 0·26 (0·09 – 0·45) |
| Guernsey | 13·57 (4·04 – 27·43) | 0·56 (0·18 – 1·25) |
| Iceland | 1·17 (0·35 – 2·46) | 0·05 (0·01 – 0·10) |
| Ireland | 4·79 (1·67 – 9·16) | 0·18 (0·06 – 0·35) |
| Isle of Man | 4·94 (1·53 – 9·62) | 0·23 (0·07 – 0·47) |
| Israel | 4·72 (1·55 – 8·87) | 0·19 (0·06 – 0·34) |
| Italy | 5·94 (2·01 – 10·86) | 0·26 (0·09 – 0·47) |
| Jersey | 13·18 (4·02 – 26·43) | 0·54 (0·15 – 1·10) |
| Lichtenstein | 31·44 (9·19 – 71·10) | 1·19 (0·33 – 2·79) |
| Luxembourg | 2·51 (0·83 – 4·87) | 0·11 (0·04 – 0·22) |
| Malta | 3·82 (1·26 – 7·18) | 0·18 (0·05 – 0·34) |
| Monaco | 2·38 (0·79 – 4·55) | 0·12 (0·04 – 0·24) |
| Netherlands | 3·50 (1·18 – 6·69) | 0·16 (0·05 – 0·33) |
| Norway | 1·74 (0·54 – 3·79) | 0·08 (0·02 – 0·16) |
| Portugal | 8·50 (3·13 – 15·41) | 0·38 (0·12 – 0·68) |
| San Marino | 3·76 (1·21 – 6·88) | 0·16 (0·05 – 0·30) |
| Slovenia | 7·68 (2·51 – 14·17) | 0·35 (0·11 – 0·65) |
| Spain | 5·10 (1·85 – 9·10) | 0·22 (0·07 – 0·39) |
| Sweden | 1·56 (0·46 – 3·17) | 0·07 (0·02 – 0·15) |
| Switzerland | 4·25 (1·44 – 7·66) | 0·19 (0·06 – 0·36) |
| United Kingdom | 2·24 (0·72 – 4·27) | 0·10 (0·03 – 0·20) |
| Non-independent colonies, states, and territories | |  |
| *Faroe islands* | 5·42 (1·69 – 11·83) | 0·23 (0·07 – 0·53) |
| *Greenland* | 0·62 (0·16 – 1·41) | 0·04 (0·01 – 0·11) |
| **Europe region, stratum B (EUR B)** | | |
| Albania | 6·52 (2·22 – 12·20) | 0·31 (0·12 – 0·56) |
| Armenia | 3·51 (1·20 – 6·28) | 0·20 (0·08 – 0·36) |
| Azerbaijan | 3·98 (1·37 – 6·95) | 0·22 (0·09 – 0·39) |
| Bosnia and Herzegovina | 7·31 (2·41 – 13·43) | 0·36 (0·14 – 0·68) |
| Bulgaria | 3·18 (1·07 – 5·72) | 0·19 (0·07 – 0·33) |
| Georgia | 5·13 (1·88 – 9·02) | 0·28 (0·11 – 0·48) |
| Kosovo | 6·94 (2·24 – 13·19) | 0·32 (0·12 – 0·61) |
| Kyrgyzstan | 6·28 (2·26 – 12·00) | 0·34 (0·13 – 0·62) |
| Montenegro | 4·09 (1·47 – 7·32) | 0·21 (0·08 – 0·37) |
| Poland | 3·48 (1·08 – 6·95) | 0·17 (0·06 – 0·31) |
| Romania | 4·52 (1·49 – 8·21) | 0·24 (0·09 – 0·43) |
| Serbia | 4·65 (1·55 – 8·48) | 0·25 (0·10 – 0·44) |
| Slovakia | 4·46 (1·57 – 8·73) | 0·22 (0·08 – 0·39) |
| Tajikistan | 9·22 (2·82 – 18·19) | 0·43 (0·16 – 0·80) |
| The Former Yugoslav Republic of Macedonia | 4·29 (1·47 – 7·87) | 0·22 (0·08 – 0·39) |
| Turkey | 3·42 (1·19 – 6·12) | 0·16 (0·06 – 0·28) |
| Turkmenistan | 3·73 (1·32 – 6·86) | 0·23 (0·09 – 0·39) |
| Uzbekistan | 7·05 (2·45 – 13·18) | 0·34 (0·13 – 0·60) |
| **Europe region, stratum C (EUR C)** | | |
| Belarus | 1·59 (0·52 – 3·19) | 0·10 (0·03 – 0·19) |
| Estonia | 1·76 (0·59 – 3·60) | 0·09 (0·03 – 0·19) |
| Hungary | 3·14 (0·98 – 5·73) | 0·17 (0·06 – 0·30) |
| Kazakhstan | 2·29 (0·81 – 4·33) | 0·15 (0·06 – 0·27) |
| Latvia | 1·65 (0·53 – 3·30) | 0·10 (0·03 – 0·20) |
| Lithuania | 1·90 (0·61 – 3·80) | 0·11 (0·04 – 0·21) |
| Republic of Moldova | 6·65 (2·18 – 12·79) | 0·32 (0·11 – 0·60) |
| Russian Federation | 1·08 (0·31 – 2·34) | 0·07 (0·02 – 0·15) |
| Ukraine | 1·90 (0·67 – 3·58) | 0·12 (0·05 – 0·22) |
| **South East Asia region, stratum B (SEAR B)** | | |
| Indonesia | 39·20 (12·76 – 77·96) | 2·15 (0·81 – 0·01) |
| Sri Lanka | 300·60 (96·54 – 604·23) | 17·98 (6·19 – 0·47) |
| Thailand | 39·37 (14·16 – 77·07) | 2·06 (0·72 – 0·02) |
| Timor-Leste | 114·14 (37·57 – 214·25) | 7·56 (2·67 – 3·21) |
| **South East Asia region, stratum D (SEAR D)** | | |
| Bangladesh | 19·23 (6·58 – 35·79) | 1·00 (0·30 – 1·82) |
| Bhutan | 11·16 (4·06 – 19·93) | 0·64 (0·21 – 1·09) |
| Democratic People’s Republic of Korea | 3·37 (1·17 – 6·01) | 0·20 (0·07 – 0·36) |
| India | 19·69 (6·81 – 36·81) | 1·12 (0·38 – 1·95) |
| Maldives | 273·57 (85·47 – 536·69) | 11·04 (3·63 – 21·21) |
| Myanmar | 10·81 (3·71 – 18·72) | 0·90 (0·32 – 1·51) |
| Nepal | 16·70 (5·63 – 32·21) | 0·91 (0·30 – 1·70) |
| **Western Pacific region, stratum A (WPR A)** | | |
| Australia | 10·51 (3·42 – 19·20) | 0·45 (0·16 – 0·79) |
| Brunei Darussalam | 32·44 (9·60 – 63·48) | 1·33 (0·45 – 2·79) |
| Japan | 5·46 (1·84 – 10·09) | 0·25 (0·10 – 0·47) |
| New Zealand | 3·48 (1·11 – 6·45) | 0·15 (0·06 – 0·28) |
| Singapore | 31·03 (8·15 – 69·54) | 1·47 (0·43 – 3·72) |
| **Western Pacific region, stratum B (WPR B)** | | |
| Cambodia | 33·65 (10·68 – 63·81) | 1·83 (0·65 – 3·35) |
| China | 10·54 (3·52 – 18·39) | 0·50 (0·19 – 0·89) |
| Fiji | 54·38 (18·35 – 101·12) | 3·08 (1·25 – 5·50) |
| Kiribati | 106·25 (36·04 – 194·67) | 6·23 (2·28 – 10·96) |
| Lao People’s Democratic Republic | 19·11 (6·61 – 34·07) | 1·09 (0·44 – 1·91) |
| Malaysia | 36·98 (11·36 – 73·20) | 1·68 (0·64 – 3·42) |
| Marshall Islands | 25·71 (9·23 – 47·42) | 2·23 (0·90 – 4·15) |
| Micronesia, Federated States of | 245·09 (77·30 – 490·19) | 12·42 (4·59 – 22·37) |
| Mongolia | 2·65 (0·87 – 5·07) | 0·14 (0·05 – 0·25) |
| Nauru | 19·90 (5·48 – 44·02) | 1·93 (0·65 – 3·98) |
| Palau | 64·06 (20·93 – 120·03) | 3·80 (1·38 – 7·30) |
| Papua New Guinea | 195·22 (61·38 – 370·88) | 12·48 (4·68 – 24·09) |
| Philippines | 14·98 (4·94 – 26·91) | 0·74 (0·26 – 1·31) |
| Republic of Korea | 5·02 (1·69 – 8·77) | 0·22 (0·08 – 0·39) |
| Samoa | 136·69 (47·42 – 274·47) | 7·65 (3·08 – 14·22) |
| Solomon Islands | 262·93 (84·11 – 518·76) | 12·11 (4·37 – 22·80) |
| Tonga | 116·07 (38·15 – 225·64) | 5·92 (2·17 – 11·28) |
| Tuvalu | 74·23 (24·60 – 131·78) | 5·03 (1·86 – 8·72) |
| Vanuatu | 121·15 (41·99 – 233·46) | 6·41 (2·45 – 11·79) |
| Viet Nam | 49·69 (16·47 – 98·92) | 2·09 (0·79 – 4·15) |
| Non-independent colonies, states, and territories |  |  |
| *French Polynesia* | 97·49 (32·20 – 179·82) | 4·55 (1·67 – 8·01) |
| *Hong Kong* | 10·37 (3·28 – 20·19) | 0·42 (0·15 – 0·83) |
| *Macao* | 9·72 (3·02 – 19·03) | 0·37 (0·13 – 0·71) |
| *New Caledonia* | 68·53 (22·89 – 124·93) | 2·95 (1·13 – 5·36) |
| *Taiwan* | 13·14 (4·60 – 24·44) | 0·54 (0·20 – 0·97) |
| *Wallis and Funtura* | 745·65 (209·01 – 1754·34) | 27·20 (8·41 – 61·99) |

Estimates unavailable for the following territories due to missing data: Azores, Falkland Islands, Gibraltar, Svalbard and Jan Mayen Islands. ^a^Morbidity and mortality are reported in cases or deaths per 100,000 population. ^c^Total includes non-independent colonies, states, and territories. ^d^Turks and Caicos estimate based on population data from Bermuda.
