# Supplementary material for: Global Morbidity and Mortality of Leptospirosis: A Systematic Review
Source: PLoS Negl Trop Dis. 2015 Sep 17;9(9):e0003898. doi: 10.1371/journal.pntd.0003898 (PMC4574773; doi:10.1371/journal.pntd.0003898)
Supplement: S13 Table — (DOCX) [file pntd.0003898.s016.docx]

**S13 Table: Estimated age group and gender-specific leptospirosis morbidity and mortality, according to WHO sub-region.**

| Demographic | Morbidity^a^ (95% CI) | | Cases (95% CI) | Mortality^a^ (95% CI) | Deaths (95% CI) |
| --- | --- | --- | --- | --- | --- |
| **All WHO sub-regions** |  | |  |  |  |
| Females |  | |  |  |  |
| 0 – 9 | 1·06 (0·41 – 1·97) | | 6400 (2500 – 12 000) | 0·22 (0·10– 0·37) | 1400 (600 – 2300) |
| 10 – 19 | 5·05 (1·82 – 8·95) | | 29 700 (10 700 – 52 700) | 0·32 (0·13 – 0·55) | 1900 (800 – 3200) |
| 20 – 29 | 8·52 (3·36 – 15·41) | | 48 700 (19 200 – 88 000) | 0·17 (0·07 – 0·29) | 1000 (400 – 1600) |
| 30 – 39 | 9·56 (3·72 – 17·36) | | 46 900 (18 200 – 85 200) | 0·09 (0·04 – 0·15) | 500 (200 – 80) |
| 40 – 49 | 7·89 (3·06 – 14·67) | | 34 100 (13 200 – 63 400) | 0·58 (0·24 – 0·99) | 2500 (1000 – 4300) |
| 50 – 59 | 8·13 (3·15 – 14·58) | | 27 100 (10 500 – 48 700) | 0·53 (0·22 – 0·93) | 1800 (800 – 3100) |
| 60 – 69 | 8·08 (3·02 – 14·72) | | 17 300 (6460 – 31 500) | 0·49 (0·20 – 0·84) | 1100 (400 – 1800) |
| 70 and older | 4·49 (1·67 – 8·29) | | 9000 (3400 – 16 700) | 0·52 (0·21 – 0·89) | 1100 (400 – 1800) |
| Males |  | |  |  |  |
| 0 – 9 | 6·76 (2·68 – 13·03) | | 44 100 (17 500 – 85 000) | 0·59 (0·24 – 0·97) | 3900 (1600 – 6400) |
| 10 – 19 | 24·73 (9·62 – 44·90) | | 155 000 (60 500 – 282 000) | 0·48 (0·2 – 0·81) | 3000 (1200 – 5100) |
| 20 – 29 | 35·27 (13·79 – 63·89) | | 211 000 (82 400 – 382 000) | 1·13 (0·45 – 2·00) | 6700 (2700 – 11 900) |
| 30 – 39 | 31·65 (11·93 – 59·71) | | 160 000 (60 300 – 302 000) | 1·10 (0·45 – 1·88) | 5600 (2300 – 9500) |
| 40 – 49 | 25·93 (10·13 – 47·41) | | 114 000 (44 300 – 208 000) | 2·82 (1·15 – 4·94) | 12 300 (5000 – 21 600) |
| 50 – 59 | 23·56 (8·69 – 43·96) | | 77 300 (28 500 – 144 000) | 2·89 (1·22 – 4·95) | 9500 (4000 – 16 300) |
| 60 – 69 | 18·19 (6·59 – 32·43) | | 36 200 (13 100 – 64 500) | 2·31 (0·90 – 3·86) | 4600 (1800 – 7700) |
| 70 and older | 9·97 (3·88 – 18·68) | | 14 800 (5800 – 27 800) | 1·57 (0·64 – 2·66) | 2300 (1000 – 4000) |
| **Africa region, stratum D (AFR D)** |  | |  |  |  |
| Females |  | |  |  |  |
| 0 – 9 | 0·86 (0·31 – 1·49) | | 493 (177 – 853) | 0·28 (0·10 – 0·48) | 160 (56 – 278) |
| 10 – 19 | 4·03 (1·45 – 6·96) | | 1779 (639 – 3074) | 0·40 (0·14 – 0·70) | 178 (60 – 307) |
| 20 – 29 | 6·81 (2·50 – 12·02) | | 2352 (864 – 4155) | 0·25 (0·08 – 0·44) | 86 (28 – 151) |
| 30 – 39 | 8·00 (2·99 – 13·93) | | 1934 (723 – 3367) | 0·16 (0·05 – 0·27) | 39 (13 – 66) |
| 40 – 49 | 7·35 (2·60 – 13·02) | | 1189 (421 – 2106) | 0·82 (0·28 – 1·44) | 132 (46 – 232) |
| 50 – 59 | 8·01 (2·79 – 13·96) | | 869 (302 – 1515) | 0·81 (0·28 – 1·41) | 88 (30 – 153) |
| 60 – 69 | 8·59 (3·21 – 14·98) | | 562 (210 – 981) | 0·81 (0·29 – 1·37) | 53 (19 – 90) |
| 70 and older | 5·21 (1·87 – 9·40) | | 209 (75 – 376) | 0·94 (0·33 – 1·63) | 38 (13 – 65) |
| Males |  | |  |  |  |
| 0 – 9 | 4·13 (1·41 – 7·24) | | 2432 (828 – 4262) | 0·65 (0·22 – 1·18) | 385 (131 – 697) |
| 10 – 19 | 15·05 (5·74 – 26·32) | | 6792 (2589 – 11877) | 0·57 (0·19 – 1·02) | 257 (87 – 459) |
| 20 – 29 | 22·73 (7·77 – 39·99) | | 7902 (2701 – 13906) | 1·18 (0·41 – 2·00) | 410 (143 – 695) |
| 30 – 39 | 21·81 (7·60 – 38·99) | | 5294 (1844 – 9465) | 1·24 (0·42 – 2·15) | 301 (101 – 523) |
| 40 – 49 | 20·10 (6·92 – 34·59) | | 3192 (1099 – 5491) | 2·89 (0·98 – 5·04) | 459 (156 – 800) |
| 50 – 59 | 19·62 (6·61 – 34·36) | | 2005 (675 – 3511) | 3·04 (1·06 – 5·07) | 311 (108 – 518) |
| 60 – 69 | 16·81 (5·74 – 29·35) | | 977 (334 – 1706) | 2·91 (1·02 – 5·00) | 169 (59 – 291) |
| 70 and older | 12·08 (4·25 – 21·35) | | 386 (136 – 683) | 2·46 (0·85 – 4·39) | 79 (27 – 141) |
| **Africa region, stratum E (AFR E)** |  | |  |  |  |
| Females |  | |  |  |  |
| 0 – 9 | 1·72 (0·55 – 3·23) | | 1159 (372 – 2173) | 0·58 (0·22 – 1·03) | 387 (148 – 694) |
| 10 – 19 | 9·00 (3·21 – 17·40) | | 4680 (1671 – 9053) | 0·86 (0·31 – 1·62) | 445 (161 – 841) |
| 20 – 29 | 15·55 (5·34 – 29·92) | | 6177 (2122 – 11886) | 0·52 (0·18 – 0·97) | 206 (73 – 386) |
| 30 – 39 | 17·97 (6·37 – 36·80) | | 4650 (1649 – 9521) | 0·32 (0·13 – 0·59) | 83 (33 – 154) |
| 40 – 49 | 15·86 (5·09 – 31·56) | | 2656 (853 – 5285) | 1·67 (0·61 – 3·30) | 280 (103 – 552) |
| 50 – 59 | 16·77 (5·61 – 32·38) | | 2038 (682 – 3937) | 1·53 (0·60 – 2·85) | 186 (73 – 346) |
| 60 – 69 | 17·67 (6·15 – 34·91) | | 1355 (471 – 2676) | 1·56 (0·56 – 3·01) | 119 (43 – 231) |
| 70 and older | 10·54 (3·30 – 22·35) | | 507 (159 – 1075) | 1·90 (0·69 – 3·75) | 91 (33 – 181) |
| Males |  | |  |  |  |
| 0 – 9 | 9·50 (3·18 – 19·58) | | 6467 (2166 – 13332) | 1·44 (0·55 – 2·73) | 979 (373 – 1859) |
| 10 – 19 | 36·74 (11·62 – 74·51) | | 19214 (6078 – 38964) | 1·22 (0·46 – 2·27) | 640 (241 – 1188) |
| 20 – 29 | 54·83 (18·25 – 108·81) | | 21756 (7241 – 43174) | 2·75 (1·05 – 5·51) | 1091 (415 – 2187) |
| 30 – 39 | 51·65 (16·41 – 100·83) | | 13565 (4310 – 26482) | 2·80 (1·10 – 5·59) | 735 (288 – 1469) |
| 40 – 49 | 46·38 (15·00 – 97·38) | | 7447 (2408 – 15635) | 6·59 (2·32 – 11·96) | 1058 (372 – 1920) |
| 50 – 59 | 43·29 (14·13 – 86·65) | | 4572 (1493 – 9153) | 6·67 (2·41 – 12·72) | 704 (254 – 1344) |
| 60 – 69 | 36·09 (12·15 – 75·00) | | 2344 (789 – 4872) | 5·92 (2·03 – 11·45) | 384 (132 – 743) |
| 70 and older | 25·78 (8·72 – 52·48) | | 928 (314 – 1889) | 4·83 (1·66 – 9·17) | 174 (60 – 330) |
| **Americas region, stratum A (AMR A)** |  | |  |  |  |
| Females |  | |  |  |  |
| 0 – 9 | 0·16 (0·04 – 0·34) | | 37 (9 – 80) | 0·04 (0·01 – 0·07) | 8 (3 – 17) |
| 10 – 19 | 1·31 (0·34 – 2·69) | | 320 (83 – 659) | 0·06 (0·02 – 0·13) | 15 (5 – 31) |
| 20 – 29 | 2·62 (0·66 – 5·77) | | 663 (166 – 1460) | 0·03 (0·01 – 0·07) | 8 (3 – 17) |
| 30 – 39 | 3·37 (0·82 – 7·55) | | 825 (200 – 1850) | 0·02 (0·01 – 0·04) | 5 (2 – 10) |
| 40 – 49 | 2·97 (0·83 – 6·30) | | 791 (221 – 1675) | 0·16 (0·06 – 0·33) | 42 (15 – 89) |
| 50 – 59 | 3·05 (0·83 – 6·63) | | 770 (210 – 1672) | 0·13 (0·05 – 0·29) | 33 (12 – 74) |
| 60 – 69 | 3·39 (0·89 – 7·65) | | 606 (159 – 1365) | 0·13 (0·04 – 0·31) | 23 (8 – 55) |
| 70 and older | 1·72 (0·42 – 3·71) | | 339 (84 – 732) | 0·19 (0·06 – 0·38) | 37 (13 – 75) |
| Males |  | |  |  |  |
| 0 – 9 | 1·34 (0·31 – 2·88) | | 337 (78 – 725) | 0·10 (0·03 – 0·24) | 26 (8 – 59) |
| 10 – 19 | 7·35 (1·85 – 16·17) | | 1895 (477 – 4168) | 0·09 (0·03 – 0·20) | 24 (8 – 50) |
| 20 – 29 | 12·35 (3·24 – 27·60) | | 3261 (856 – 7288) | 0·27 (0·08 – 0·55) | 70 (22 – 147) |
| 30 – 39 | 11·56 (3·25 – 25·14) | | 2886 (811 – 6275) | 0·29 (0·10 – 0·62) | 71 (24 – 154) |
| 40 – 49 | 10·46 (2·91 – 23·48) | | 2752 (766 – 6177) | 0·81 (0·29 – 1·71) | 214 (75 – 450) |
| 50 – 59 | 9·37 (2·28 – 20·97) | | 2266 (551 – 5072) | 0·83 (0·27 – 1·75) | 200 (66 – 423) |
| 60 – 69 | 8·08 (1·93 – 17·56) | | 1325 (317 – 2879) | 0·75 (0·25 – 1·63) | 124 (40 – 267) |
| 70 and older | 5·28 (1·47 – 11·63) | | 740 (205 – 1629) | 0·58 (0·20 – 1·26) | 81 (28 – 176) |
| **Americas region, stratum B (AMR B)** |  | |  |  |  |
| Females |  | |  |  |  |
| 0 – 9 | 0·46 (0·16 – 0·90) | | 197 (66 – 384) | 0·12 (0·04 – 0·23) | 49 (17 – 97) |
| 10 – 19 | 3·77 (1·19 – 7·84) | | 1646 (521 – 3428) | 0·19 (0·07 – 0·38) | 84 (29 – 166) |
| 20 – 29 | 7·59 (2·36 – 15·74) | | 3194 (992 – 6623) | 0·11 (0·04 – 0·22) | 46 (16 – 94) |
| 30 – 39 | 9·24 (2·82 – 20·61) | | 3471 (1060 – 7742) | 0·06 (0·02 – 0·12) | 22 (8 – 44) |
| 40 – 49 | 8·38 (2·61 – 18·89) | | 2622 (815 – 5910) | 0·49 (0·16 – 0·99) | 152 (51 – 311) |
| 50 – 59 | 9·17 (2·76 – 19·98) | | 2122 (637 – 4623) | 0·46 (0·16 – 0·96) | 106 (38 – 222) |
| 60 – 69 | 9·83 (3·28 – 22·31) | | 1407 (470 – 3194) | 0·44 (0·15 – 0·90) | 63 (21 – 129) |
| 70 and older | 4·86 (1·52 – 10·16) | | 633 (198 – 1324) | 0·56 (0·19 – 1·15) | 73 (25 – 150) |
| Males |  | |  |  |  |
| 0 – 9 | 3·93 (1·28 – 7·88) | | 1737 (564 – 3486) | 0·36 (0·13 – 0·73) | 161 (59 – 322) |
| 10 – 19 | 21·45 (6·63 – 43·37) | | 9632 (2975 – 19472) | 0·31 (0·11 – 0·64) | 137 (50 – 288) |
| 20 – 29 | 37·36 (12·53 – 79·27) | | 15706 (5266 – 33324) | 0·87 (0·32 – 1·80) | 368 (136 – 758) |
| 30 – 39 | 33·24 (10·49 – 67·33) | | 12121 (3826 – 24548) | 0·87 (0·30 – 1·71) | 316 (108 – 623) |
| 40 – 49 | 30·11 (9·53 – 66·73) | | 8910 (2820 – 19745) | 2·68 (0·93 – 5·75) | 793 (274 – 1702) |
| 50 – 59 | 28·67 (9·05 – 59·88) | | 6153 (1943 – 12851) | 2·85 (1·06 – 6·07) | 611 (227 – 1302) |
| 60 – 69 | 22·87 (6·93 – 49·30) | | 2913 (883 – 6280) | 2·35 (0·79 – 4·97) | 299 (100 – 633) |
| 70 and older | 14·58 (4·71 – 30·17) | | 1391 (450 – 2877) | 1·73 (0·58 – 3·60) | 165 (55 – 344) |
| **Americas region, stratum D (AMR D)** |  | |  |  |  |
| Females |  | |  |  |  |
| 0 – 9 | 0·88 (0·28 – 1·68) | | 82 (26 – 157) | 0·23 (0·07 – 0·48) | 22 (6 – 45) |
| 10 – 19 | 6·15 (1·83 – 12·10) | | 541 (161 – 1064) | 0·38 (0·11 – 0·78) | 33 (10 – 69) |
| 20 – 29 | 12·55 (3·80 – 25·84) | | 931 (282 – 1915) | 0·22 (0·07 – 0·45) | 16 (5 – 33) |
| 30 – 39 | 14·59 (4·60 – 29·82) | | 837 (264 – 1711) | 0·12 (0·04 – 0·25) | 7 (2 – 15) |
| 40 – 49 | 13·24 (3·88 – 26·38) | | 563 (165 – 1122) | 0·86 (0·26 – 1·77) | 37 (11 – 75) |
| 50 – 59 | 14·99 (4·54 – 30·41) | | 451 (137 – 916) | 0·83 (0·24 – 1·64) | 25 (7 – 50) |
| 60 – 69 | 16·07 (4·83 – 35·49) | | 303 (91 – 670) | 0·82 (0·24 – 1·69) | 15 (4 – 32) |
| 70 and older | 8·34 (2·49 – 17·99) | | 134 (40 – 289) | 1·06 (0·31 – 2·20) | 17 (5 – 35) |
| Males |  | |  |  |  |
| 0 – 9 | 6·39 (2·03 – 13·10) | | 622 (197 – 1276) | 0·65 (0·19 – 1·27) | 63 (19 – 124) |
| 10 – 19 | 33·59 (10·42 – 70·54) | | 3034 (941 – 6372) | 0·57 (0·18 – 1·16) | 52 (16 – 105) |
| 20 – 29 | 56·56 (16·88 – 118·19) | | 4181 (1248 – 8739) | 1·49 (0·47 – 3·07) | 110 (35 – 227) |
| 30 – 39 | 52·64 (16·46 – 120·28) | | 2903 (907 – 6632) | 1·51 (0·44 – 3·23) | 83 (24 – 178) |
| 40 – 49 | 45·50 (14·16 – 98·11) | | 1837 (572 – 3962) | 4·36 (1·42 – 9·11) | 176 (57 – 368) |
| 50 – 59 | 45·69 (13·25 – 101·94) | | 1297 (376 – 2894) | 4·73 (1·34 – 10·24) | 134 (38 – 291) |
| 60 – 69 | 36·90 (10·36 – 77·89) | | 640 (180 – 1352) | 4·06 (1·22 – 8·69) | 70 (21 – 151) |
| 70 and older | 25·13 (6·99 – 56·10) | | 330 (92 – 736) | 3·07 (0·97 – 6·90) | 40 (13 – 91) |
| **Eastern Mediterranean region, stratum B (EMR B)** | | |  |  |  |
| Females |  | |  |  |  |
| 0 – 9 | 0·20 (0·07 – 0·37) | | 32 (11 – 58) | 0·05 (0·02 – 0·09) | 7 (2 – 14) |
| 10 – 19 | 1·48 (0·49 – 2·71) | | 220 (72 – 403) | 0·08 (0·03 – 0·14) | 11 (4 – 21) |
| 20 – 29 | 2·91 (0·97 – 5·58) | | 512 (171 – 981) | 0·04 (0·01 – 0·08) | 8 (2 – 14) |
| 30 – 39 | 3·50 (1·22 – 6·54) | | 450 (157 – 839) | 0·02 (0·01 – 0·05) | 3 (1 – 6) |
| 40 – 49 | 3·10 (1·00 – 5·95) | | 274 (88 – 527) | 0·18 (0·06 – 0·33) | 16 (5 – 29) |
| 50 – 59 | 3·43 (1·15 – 6·62) | | 193 (64 – 372) | 0·18 (0·06 – 0·33) | 10 (3 – 18) |
| 60 – 69 | 3·68 (1·20 – 7·11) | | 111 (36 – 215) | 0·17 (0·06 – 0·31) | 5 (2 – 9) |
| 70 and older | 1·97 (0·67 – 3·70) | | 46 (16 – 87) | 0·23 (0·07 – 0·41) | 5 (2 – 10) |
| Males |  | |  |  |  |
| 0 – 9 | 1·51 (0·52 – 2·79) | | 250 (86 – 461) | 0·14 (0·05 – 0·25) | 23 (8 – 41) |
| 10 – 19 | 7·81 (2·79 – 14·46) | | 1213 (433 – 2247) | 0·12 (0·04 – 0·22) | 19 (6 – 34) |
| 20 – 29 | 13·17 (4·58 – 24·79) | | 2489 (865 – 4686) | 0·32 (0·10 – 0·63) | 61 (19 – 119) |
| 30 – 39 | 12·21 (4·18 – 22·97) | | 1882 (644 – 3539) | 0·34 (0·11 – 0·62) | 53 (17 – 95) |
| 40 – 49 | 10·79 (3·64 – 19·43) | | 1142 (385 – 2058) | 0·94 (0·32 – 1·70) | 99 (34 – 180) |
| 50 – 59 | 10·47 (3·67 – 19·70) | | 674 (236 – 1269) | 1·03 (0·31 – 1·93) | 66 (20 – 124) |
| 60 – 69 | 8·51 (2·97 – 16·66) | | 261 (91 – 510) | 0·87 (0·28 – 1·66) | 27 (9 – 51) |
| 70 and older | 5·62 (1·86 – 11·06) | | 127 (42 – 250) | 0·65 (0·22 – 1·25) | 15 (5 – 28) |
| **Eastern Mediterranean region, stratum D (EMR D)** | | |  |  |  |
| Females |  | |  |  |  |
| 0 – 9 | 0·52 (0·18 – 0·95) | | 283 (96 – 520) | 0·15 (0·06 – 0·26) | 82 (31 – 144) |
| 10 – 19 | 3·08 (1·02 – 5·96) | | 1448 (480 – 2799) | 0·23 (0·09 – 0·41) | 108 (41 – 193) |
| 20 – 29 | 5·63 (1·97 – 10·74) | | 2228 (779 – 4253) | 0·13 (0·05 – 0·24) | 52 (19 – 94) |
| 30 – 39 | 6·69 (2·33 – 12·91) | | 1862 (649 – 3594) | 0·08 (0·03 – 0·15) | 23 (8 – 41) |
| 40 – 49 | 5·93 (2·09 – 11·19) | | 1183 (418 – 2230) | 0·48 (0·17 – 0·87) | 96 (35 – 174) |
| 50 – 59 | 6·57 (2·40 – 11·97) | | 895 (328 – 1630) | 0·47 (0·17 – 0·85) | 64 (23 – 115) |
| 60 – 69 | 7·04 (2·52 – 13·32) | | 588 (210 – 1114) | 0·45 (0·16 – 0·80) | 38 (14 – 67) |
| 70 and older | 3·96 (1·29 – 7·65) | | 216 (71 – 418) | 0·57 (0·21 – 1·04) | 31 (11 – 57) |
| Males |  | |  |  |  |
| 0 – 9 | 3·13 (1·05 – 5·77) | | 1793 (604 – 3309) | 0·39 (0·15 – 0·72) | 225 (88 – 410) |
| 10 – 19 | 14·04 (4·79 – 26·62) | | 6916 (2358 – 13112) | 0·33 (0·12 – 0·58) | 163 (61 – 286) |
| 20 – 29 | 22·52 (7·62 – 42·80) | | 9279 (3139 – 17635) | 0·80 (0·29 – 1·47) | 328 (120 – 604) |
| 30 – 39 | 20·42 (6·82 – 37·75) | | 5828 (1947 – 10773) | 0·83 (0·33 – 1·57) | 236 (93 – 448) |
| 40 – 49 | 18·44 (6·38 – 36·40) | | 3728 (1290 – 7359) | 2·08 (0·80 – 3·98) | 420 (162 – 804) |
| 50 – 59 | 18·00 (5·70 – 34·17) | | 2521 (798 – 4785) | 2·21 (0·80 – 4·13) | 310 (111 – 579) |
| 60 – 69 | 15·06 (4·72 – 28·73) | | 1225 (384 – 2337) | 1·93 (0·71 – 3·62) | 157 (58 – 295) |
| 70 and older | 10·32 (3·28 – 20·06) | | 504 (160 – 979) | 1·51 (0·55 – 2·91) | 74 (27 – 142) |
| **Europe region, stratum A (EUR A)** |  | |  |  |  |
| Females |  | |  |  |  |
| 0 – 9 | 0·09 (0·03 – 0·18) | | 21 (7 – 39) | 0·02 (0·01 – 0·03) | 4 (1 – 7) |
| 10 – 19 | 0·83 (0·29 – 1·52) | | 192 (67 – 350) | 0·03 (0·01 – 0·06) | 8 (3 – 14) |
| 20 – 29 | 1·77 (0·57 – 3·33) | | 464 (150 – 873) | 0·02 (0·01 – 0·03) | 5 (1 – 8) |
| 30 – 39 | 2·28 (0·79 – 4·22) | | 676 (233 – 1252) | 0·01 (0·00 – 0·02) | 3 (1 – 5) |
| 40 – 49 | 1·95 (0·62 – 3·62) | | 643 (204 – 1192) | 0·09 (0·03 – 0·16) | 29 (10 – 53) |
| 50 – 59 | 2·18 (0·73 – 4·08) | | 624 (208 – 1168) | 0·08 (0·03 – 0·15) | 24 (8 – 43) |
| 60 – 69 | 2·38 (0·78 – 4·34) | | 577 (189 – 1050) | 0·08 (0·03 – 0·14) | 20 (6 – 35) |
| 70 and older | 1·21 (0·41 – 2·26) | | 408 (138 – 760) | 0·11 (0·04 – 0·21) | 38 (13 – 69) |
| Males |  | |  |  |  |
| 0 – 9 | 0·87 (0·31 – 1·57) | | 206 (74 – 371) | 0·06 (0·02 – 0·11) | 15 (5 – 26) |
| 10 – 19 | 5·17 (1·74 – 9·63) | | 1254 (423 – 2338) | 0·05 (0·02 – 0·09) | 13 (4 – 23) |
| 20 – 29 | 9·25 (3·27 – 17·53) | | 2529 (894 – 4793) | 0·16 (0·05 – 0·28) | 43 (15 – 77) |
| 30 – 39 | 8·70 (2·87 – 15·90) | | 2649 (873 – 4841) | 0·17 (0·06 – 0·30) | 52 (17 – 92) |
| 40 – 49 | 7·51 (2·76 – 13·90) | | 2504 (920 – 4633) | 0·52 (0·17 – 0·93) | 172 (56 – 312) |
| 50 – 59 | 7·16 (2·55 – 13·30) | | 2006 (715 – 3725) | 0·55 (0·19 – 1·01) | 155 (53 – 283) |
| 60 – 69 | 5·82 (2·01 – 10·95) | | 1314 (454 – 2474) | 0·46 (0·15 – 0·84) | 105 (34 – 189) |
| 70 and older | 3·80 (1·27 – 6·96) | | 864 (288 – 1581) | 0·35 (0·12 – 0·64) | 80 (27 – 146) |
| **Europe region, stratum B (EUR B)** |  | |  |  |  |
| Females |  | |  |  |  |
| 0 – 9 | 0·18 (0·06 – 0·33) | | 30 (10 – 55) | 0·04 (0·02 – 0·08) | 7 (3 – 13) |
| 10 – 19 | 1·25 (0·41 – 2·39) | | 228 (75 – 435) | 0·07 (0·02 – 0·13) | 13 (4 – 23) |
| 20 – 29 | 2·39 (0·88 – 4·41) | | 471 (173 – 869) | 0·04 (0·01 – 0·07) | 7 (3 – 14) |
| 30 – 39 | 2·77 (0·94 – 5·28) | | 487 (165 – 928) | 0·02 (0·01 – 0·04) | 4 (1 – 7) |
| 40 – 49 | 2·47 (0·83 – 4·49) | | 367 (123 – 667) | 0·15 (0·06 – 0·26) | 22 (9 – 39) |
| 50 – 59 | 2·66 (0·92 – 5·22) | | 348 (121 – 683) | 0·14 (0·05 – 0·24) | 18 (7 – 32) |
| 60 – 69 | 2·76 (0·93 – 5·14) | | 226 (76 – 421) | 0·13 (0·05 – 0·23) | 10 (4 – 19) |
| 70 and older | 1·49 (0·53 – 2·87) | | 134 (47 – 258) | 0·17 (0·06 – 0·32) | 16 (6 – 29) |
| Males |  | |  |  |  |
| 0 – 9 | 1·31 (0·46 – 2·54) | | 229 (80 – 442) | 0·12 (0·05 – 0·23) | 22 (8 – 40) |
| 10 – 19 | 6·63 (2·26 – 12·80) | | 1259 (429 – 2432) | 0·11 (0·04 – 0·21) | 20 (8 – 41) |
| 20 – 29 | 10·45 (3·65 – 19·88) | | 2114 (738 – 4023) | 0·27 (0·09 – 0·49) | 55 (19 – 98) |
| 30 – 39 | 9·31 (2·97 – 17·79) | | 1644 (524 – 3141) | 0·27 (0·10 – 0·50) | 47 (18 – 88) |
| 40 – 49 | 8·22 (2·65 – 15·44) | | 1189 (384 – 2235) | 0·74 (0·29 – 1·36) | 107 (41 – 197) |
| 50 – 59 | 7·77 (2·57 – 14·27) | | 956 (316 – 1755) | 0·76 (0·30 – 1·41) | 94 (37 – 174) |
| 60 – 69 | 6·31 (2·26 – 11·52) | | 435 (156 – 795) | 0·62 (0·25 – 1·12) | 43 (17 – 78) |
| 70 and older | 4·30 (1·47 – 7·82) | | 249 (85 – 454) | 0·49 (0·18 – 0·87) | 28 (11 – 50) |
| **Europe region, stratum C (EUR C)** |  | |  |  |  |
| Females |  | |  |  |  |
| 0 – 9 | 0·08 (0·02 – 0·21) | | 10 (3 – 25) | 0·02 (0·01 – 0·05) | 2 (1 – 6) |
| 10 – 19 | 0·50 (0·13 – 1·18) | | 62 (17 – 147) | 0·03 (0·01 – 0·07) | 4 (1– 9) |
| 20 – 29 | 0·85 (0·23 – 2·16) | | 164 (45 – 418) | 0·02 (0·01 – 0·04) | 3 (1– 9) |
| 30 – 39 | 1·02 (0·27 – 2·34) | | 176 (47 – 406) | 0·01 (0·00 – 0·03) | 2 (1 – 5) |
| 40 – 49 | 0·94 (0·25 – 2·42) | | 160 (42 – 412) | 0·06 (0·02 – 0·15) | 11 (3– 26) |
| 50 – 59 | 1·02 (0·30 – 2·67) | | 189 (55 – 496) | 0·06 (0·02 – 0·15) | 11 (3– 29) |
| 60 – 69 | 1·12 (0·28 – 2·80) | | 130 (33 – 326) | 0·06 (0·02 – 0·15) | 7 (2 – 17) |
| 70 and older | 0·59 (0·17 – 1·49) | | 95 (27 – 238) | 0·07 (0·02 – 0·18) | 12 (3– 29) |
| Males |  | |  |  |  |
| 0 – 9 | 0·49 (0·14 – 1·14) | | 62 (18 – 143) | 0·05 (0·02 – 0·13) | 6 (2 – 16) |
| 10 – 19 | 2·14 (0·62 – 4·86) | | 279 (81 – 632) | 0·04 (0·01 – 0·11) | 6 (2 – 14) |
| 20 – 29 | 3·24 (0·86 – 7·55) | | 642 (171 – 1494) | 0·11 (0·03 – 0·28) | 21 (6 – 56) |
| 30 – 39 | 3·03 (0·90 – 7·31) | | 510 (152 – 1229) | 0·11 (0·03 – 0·27) | 18 (6 – 45) |
| 40 – 49 | 2·75 (0·80 – 6·86) | | 424 (123 – 1061) | 0·27 (0·08 – 0·69) | 42 (12 – 107) |
| 50 – 59 | 2·60 (0·73 – 6·44) | | 389 (109 – 967) | 0·28 (0·08 – 0·67) | 43 (13 – 100) |
| 60 – 69 | 2·27 (0·67 – 5·40) | | 177 (52 – 420) | 0·25 (0·08 – 0·66) | 20 (6 – 51) |
| 70 and older | 1·60 (0·42 – 4·03) | | 113 (30 – 285) | 0·20 (0·05 – 0·51) | 14 (4 – 36) |
| **South East Asia region, stratum B (SEAR B)** | |  |  |  |  |
| Females |  | |  |  |  |
| 0 – 9 | 1·91 (0·54 – 4·60) | | 516 (145 – 1243) | 0·53 (0·15 – 1·28) | 143 (41 – 346) |
| 10 – 19 | 14·23 (3·46 – 37·08) | | 3809 (926 – 9927) | 0·82 (0·26 – 1·81) | 218 (70 – 484) |
| 20 – 29 | 28·13 (6·69 – 73·10) | | 7739 (1840 – 20109) | 0·47 (0·15 – 1·15) | 129 (40 – 316) |
| 30 – 39 | 32·74 (9·51 – 77·34) | | 8388 (2438 – 19815) | 0·25 (0·07 – 0·59) | 65 (18 – 152) |
| 40 – 49 | 31·44 (7·85 – 75·38) | | 6958 (1737 – 16683) | 2·10 (0·64 – 4·98) | 465 (142 – 1102) |
| 50 – 59 | 36·18 (10·24 – 87·32) | | 5742 (1626 – 13859) | 2·12 (0·58 – 5·15) | 336 (92 – 817) |
| 60 – 69 | 40·87 (11·15 – 104·32) | | 3983 (1087 – 10168) | 2·17 (0·59 – 5·00) | 211 (57 – 487) |
| 70 and older | 20·97 (5·90 – 49·77) | | 1545 (435 – 3669) | 2·72 (0·79 – 6·62) | 201 (59 – 488) |
| Males |  | |  |  |  |
| 0 – 9 | 15·21 (4·22 – 38·13) | | 4276 (1186 – 10717) | 1·61 (0·48 – 3·63) | 452 (134 – 1019) |
| 10 – 19 | 74·89 (17·42 – 178·07) | | 20761 (4830 – 49366) | 1·29 (0·34 – 3·00) | 357 (94 – 831) |
| 20 – 29 | 131·23 (34·21 – 333·58) | | 36725 (9573 – 93354) | 3·72 (1·05 – 9·30) | 1042 (293 – 2603) |
| 30 – 39 | 110·63 (28·60 – 263·16) | | 27899 (7212 – 66361) | 3·68 (1·15 – 8·39) | 927 (289 – 2115) |
| 40 – 49 | 109·93 (26·23 – 286·09) | | 23726 (5661 – 61745) | 10·62 (3·00 – 24·55) | 2292 (647 – 5299) |
| 50 – 59 | 110·21 (28·60 – 255·16) | | 17072 (4430 – 39525) | 12·64 (3·34 – 30·27) | 1958 (518 – 4689) |
| 60 – 69 | 95·52 (27·04 – 238·90) | | 8109 (2295 – 20282) | 10·70 (3·20 – 25·85) | 908 (272 – 2195) |
| 70 and older | 60·73 (14·90 – 153·25) | | 3406 (836 – 8595) | 8·25 (2·54 – 19·66) | 463 (142 – 1103) |
| **South East Asia region, stratum D (SEAR D)** |  | |  |  |  |
| Females |  | |  |  |  |
| 0 – 9 | 0·82 (0·22 – 1·87) | | 1195 (324 – 2745) | 0·23 (0·06 – 0·51) | 332 (92 – 743) |
| 10 – 19 | 5·69 (1·60 – 13·24) | | 8157 (2287 – 18974) | 0·36 (0·10 – 0·80) | 516 (149 – 1153) |
| 20 – 29 | 10·68 (2·77 – 25·53) | | 13734 (3566 – 32829) | 0·21 (0·05 – 0·46) | 266 (67 – 587) |
| 30 – 39 | 12·41 (3·37 – 29·59) | | 12986 (3530 – 30968) | 0·12 (0·03 – 0·28) | 128 (35 – 297) |
| 40 – 49 | 11·28 (2·94 – 26·85) | | 9120 (2374 – 21717) | 0·83 (0·23 – 2·02) | 670 (185 – 1634) |
| 50 – 59 | 12·75 (3·49 – 31·03) | | 7658 (2093 – 18635) | 0·81 (0·20 – 1·89) | 487 (122 – 1138) |
| 60 – 69 | 13·60 (2·88 – 32·05) | | 4713 (998 – 11103) | 0·77 (0·21 – 1·84) | 268 (72 – 636) |
| 70 and older | 7·20 (2·11 – 16·62) | | 1716 (503 – 3961) | 1·01 (0·26 – 2·38) | 240 (62 – 567) |
| Males |  | |  |  |  |
| 0 – 9 | 5·69 (1·63 – 13·03) | | 8982 (2571 – 20570) | 0·65 (0·18 – 1·46) | 1020 (288 – 2305) |
| 10 – 19 | 27·33 (7·88 – 66·14) | | 42411 (12234 – 102630) | 0·55 (0·17 – 1·24) | 849 (260 – 1921) |
| 20 – 29 | 44·37 (11·40 – 106·24) | | 61291 (15751 – 146758) | 1·43 (0·36 – 3·34) | 1979 (492 – 4619) |
| 30 – 39 | 40·33 (10·90 – 93·21) | | 44792 (12103 – 103533) | 1·47 (0·43 – 3·28) | 1628 (475 – 3645) |
| 40 – 49 | 35·33 (9·84 – 88·46) | | 30076 (8375 – 75313) | 3·92 (0·99 – 9·68) | 3338 (839 – 8243) |
| 50 – 59 | 35·95 (9·95 – 84·58) | | 22329 (6181 – 52525) | 4·23 (1·11 – 9·89) | 2630 (687 – 6142) |
| 60 – 69 | 29·82 (8·08 – 72·10) | | 9785 (2652 – 23658) | 3·70 (0·87 – 8·74) | 1215 (287 – 2868) |
| 70 and older | 20·16 (5·33 – 47·04) | | 3993 (1056 – 9314) | 2·76 (0·74 – 6·12) | 546 (146 – 1211) |
| **Western Pacific region, stratum A (WPR A)** |  | |  |  |  |
| Females |  | |  |  |  |
| 0 – 9 | 0·15 (0·05 – 0·33) | | 11 (4 – 24) | 0·03 (0·01 – 0·06) | 2 (1 – 4) |
| 10 – 19 | 1·49 (0·42 – 3·29) | | 117 (33 – 258) | 0·06 (0·01 – 0·12) | 4 (1 – 10) |
| 20 – 29 | 3·10 (0·93 – 6·74) | | 278 (83 – 605) | 0·03 (0·01 – 0·06) | 3 (1 – 5) |
| 30 – 39 | 3·70 (1·10 – 7·96) | | 412 (123 – 887) | 0·01 (0·00 – 0·03) | 2 (0 – 3) |
| 40 – 49 | 3·49 (0·96 – 7·83) | | 371 (102 – 832) | 0·15 (0·04 – 0·34) | 16 (4 – 36) |
| 50 – 59 | 3·87 (1·17 – 8·56) | | 399 (121 – 884) | 0·14 (0·04 – 0·32) | 15 (4 – 33) |
| 60 – 69 | 3·92 (1·13 – 9·22) | | 426 (123 – 999) | 0·12 (0·03 – 0·28) | 13 (4 – 30) |
| 70 and older | 1·84 (0·45 – 4·58) | | 255 (63 – 635) | 0·16 (0·04 – 0·36) | 23 (6 – 50) |
| Males |  | |  |  |  |
| 0 – 9 | 1·51 (0·44 – 3·29) | | 114 (33 – 248) | 0·11 (0·03 – 0·23) | 8 (2 – 17) |
| 10 – 19 | 9·86 (2·98 – 21·73) | | 815 (247 – 1796) | 0·09 (0·03 – 0·20) | 8 (2 – 17) |
| 20 – 29 | 17·47 (4·56 – 37·84) | | 1641 (429 – 3555) | 0·28 (0·08 – 0·63) | 26 (7 – 60) |
| 30 – 39 | 15·11 (4·53 – 33·84) | | 1728 (518 – 3870) | 0·27 (0·08 – 0·63) | 31 (9 – 72) |
| 40 – 49 | 13·83 (3·64 – 32·12) | | 1478 (390 – 3435) | 0·95 (0·27 – 2·18) | 101 (29 – 233) |
| 50 – 59 | 13·50 (3·93 – 30·45) | | 1379 (401 – 3110) | 1·03 (0·28 – 2·46) | 105 (28 – 251) |
| 60 – 69 | 9·87 (2·86 – 24·45) | | 1007 (292 – 2495) | 0·78 (0·21 – 1·77) | 79 (21 – 181) |
| 70 and older | 5·79 (1·64 – 13·03) | | 554 (157 – 1247) | 0·54 (0·16 – 1·21) | 52 (15 – 116) |
| **Western Pacific region, stratum B (WPR B)** |  | |  |  |  |
| Females |  | |  |  |  |
| 0 – 9 | 0·51 (0·16 – 1·04) | | 544 (172 – 1104) | 0·13 (0·05 – 0·26) | 140 (52 – 281) |
| 10 – 19 | 3·71 (1·17 – 7·86) | | 4510 (1417 – 9565) | 0·20 (0·07 – 0·41) | 245 (89 – 494) |
| 20 – 29 | 6·95 (2·05 – 13·74) | | 9328 (2746 – 18440) | 0·10 (0·03 – 0·21) | 133 (45 – 276) |
| 30 – 39 | 8·28 (2·42 – 18·58) | | 10463 (3054 – 23494) | 0·05 (0·02 – 0·11) | 67 (23 – 141) |
| 40 – 49 | 6·91 (2·27 – 14·47) | | 8962 (2941 – 18770) | 0·41 (0·14 – 0·87) | 529 (188 – 1135) |
| 50 – 59 | 7·60 (2·43 – 16·00) | | 7080 (2268 – 14910) | 0·38 (0·12 – 0·78) | 354 (113 – 730) |
| 60 – 69 | 8·03 (2·61 – 16·74) | | 4432 (1442 – 9242) | 0·35 (0·12 – 0·76) | 192 (68 – 422) |
| 70 and older | 4·18 (1·23 – 9·13) | | 1952 (575 – 4265) | 0·46 (0·15 – 0·93) | 217 (70 – 436) |
| Males |  | |  |  |  |
| 0 – 9 | 3·99 (1·28 – 7·91) | | 4987 (1601 – 9889) | 0·38 (0·14 – 0·74) | 475 (172 – 931) |
| 10 – 19 | 21·32 (6·57 – 43·98) | | 29703 (9161 – 61280) | 0·30 (0·10 – 0·61) | 423 (138 – 857) |
| 20 – 29 | 33·54 (10·40 – 69·81) | | 48248 (14955 – 100430) | 0·77 (0·28 – 1·48) | 1107 (399 – 2122) |
| 30 – 39 | 30·22 (9·45 – 61·67) | | 39761 (12433 – 81127) | 0·79 (0·26 – 1·63) | 1041 (346 – 2142) |
| 40 – 49 | 24·78 (7·16 – 53·44) | | 33299 (9620 – 71814) | 2·13 (0·73 – 4·33) | 2857 (978 – 5813) |
| 50 – 59 | 23·89 (6·99 – 52·34) | | 22735 (6647 – 49794) | 2·21 (0·70 – 4·63) | 2101 (665 – 4401) |
| 60 – 69 | 18·32 (5·20 – 40·10) | | 10192 (2894 – 22313) | 1·82 (0·59 – 3·77) | 1015 (328 – 2097) |
| 70 and older | 11·88 (3·73 – 25·70) | | 4684 (1471 – 10135) | 1·38 (0·45 – 3·10) | 543 (176 – 1221) |

^a^Morbidity and mortality are reported in cases or deaths per 100,000 population.
